# Does the Quantum Layer Help? A Leakage-Aware Benchmark of Hybrid Quantum–Classical Models for Brain Tumor MRI Classification

**DOI:** 10.3390/diagnostics16213485

**Published:** 2026-10-24

**Authors:** Tuan-Linh Duong, Pande Wikantyasa Maswita, Tony Sumaryada, Sitti Yani, Thanh-Tai Duong, Hiba Omer, Nissren Tamam, Abdelmoneim Sulieman

**Affiliations:** 1 The Laboratory Center, Hanoi University of Public Health, Hanoi 100000, Vietnam; dtl1@huph.edu.vn; 2 Department of Physics, Faculty of Mathematics and Natural Sciences, IPB University, Bogor 16680, Indonesia; pandeww99@gmail.com (P.W.M.); tsumaryada@apps.ipb.ac.id (T.S.); sittiyani@apps.ipb.ac.id (S.Y.); 3 Department of Medical Physics, Faculty of Medicine, Nguyen Tat Thanh University, 298-300A Nguyen Tat Thanh Street, Ho Chi Minh City 700000, Vietnam; 4 Department of Radiological Sciences, College of Applied Medical Sciences, Imam Abdulrahman Bin Faisal University, P.O. Box 1982, Dammam 34212, Saudi Arabia; hbomer@iau.edu.sa; 5 Department of Physics, College of Sciences, Princess Nourah bint Abdulrahman University, P.O. Box 84428, Riyadh 11671, Saudi Arabia; nissrentamam@gmail.com; 6 Department of Radiological Sciences, College of Applied Medical Sciences, King Saud bin Abdulaziz University for Health Sciences, P.O. Box 6664, Al Ahsa 31982, Saudi Arabia; abdelmoneim_a@yahoo.com; 7 Imaging Physics Section, Research and Innovation Department, King Faisal Specialist Hospital & Research Center, P.O. Box 3354, Riyadh 11211, Saudi Arabia

**Keywords:** quantum machine learning, variational quantum circuit, hybrid quantum–classical neural network, brain tumor, magnetic resonance imaging, data leakage, deep learning, benchmarking

## Abstract

**Background/Objectives:** Hybrid quantum–classical neural networks are reported to classify brain tumors on magnetic resonance imaging (MRI) accurately, but these reports rarely control for data leakage or test whether the quantum circuit contributes. We examined both issues. **Methods:** We audited version 2 of the Kaggle Brain Tumor MRI Dataset (7200 images) with mirror- and rotation-invariant duplicate detection and by matching every image to its source collections, which recovered patient identities for 4846 tumor images. A variational quantum circuit (VQC) was compared with size-matched, deeper and Fourier-surrogate classical controls using frozen features, end-to-end fine-tuning and training from scratch, all under patient-grouped five-fold cross-validation. **Results:** Of the 1600 test images, 1269 (79%) had a copy in the training partition, and 915 of the 917 figshare-derived test images shared a patient with it. On frozen features, a six-qubit VQC head was 6.0 percentage points (pp) less accurate than its classical control (95% confidence interval −6.8 to −5.2) and less accurate than a classical surrogate of its own function class. With fine-tuning, it stayed within about 1 pp of its control but was 0.7 pp less accurate (−1.2 to −0.1) after convergence-based training; a hybrid network trained from scratch was 8.3 pp less accurate than its control. Patient-grouped evaluation lowered accuracy by 3–8 pp relative to our earlier duplicate-grouped evaluation, and 76 glioma test images from another source remained poorly recognized. **Conclusions:** The quantum layer added cost but no accuracy, and leakage and provenance mattered more than the classifier head.

## 1. Introduction

Brain tumors are a heterogeneous group of neoplasms whose diagnosis and grading combine imaging with histological and molecular criteria [1], and primary brain and other central nervous system tumors are diagnosed in tens of thousands of patients each year in the United States alone [2]. Magnetic resonance imaging (MRI) is the main imaging modality, and convolutional neural networks (CNNs) trained on public MRI collections routinely report 95–99% accuracy for distinguishing glioma, meningioma, pituitary tumor and scans without a tumor [3,4,5,6]. Much of this literature relies on one public dataset distributed through Kaggle, which merges three earlier collections [3,7,8,9].

In parallel, quantum machine learning has been proposed as a way to learn from data in exponentially large Hilbert spaces [10,11]. On noisy intermediate-scale quantum hardware [12], most proposals use variational quantum circuits (VQCs), that is, parameterized circuits trained by classical gradient descent. For images, VQCs are usually embedded in classical networks, as “dressed” circuits between classical layers [13], as quanvolutional filters [14] or as quantum convolutional networks [15], and such hybrid models have been applied to medical images [16,17,18]. For brain tumor MRI in particular, several hybrid quantum–classical models have been reported to match or exceed classical CNNs with fewer trainable parameters [19,20,21,22], and studies published in 2025–2026 report 94–98.6% accuracy for hybrid models on brain MRI tasks [23,24,25,26,27,28,29].

Two issues make such claims hard to interpret. The first is the comparison. A hybrid network contains a classical encoder, a classical projection into the circuit and a classical read-out, so its overall accuracy says little about the quantum layer itself. A large benchmark of quantum classifiers found that out-of-the-box classical models usually outperformed them and that removing entanglement often did not hurt [30], and it has been argued that understanding what near-term quantum models learn is a better research goal than chasing quantum advantage [31]. The informative control for a hybrid network is the same network with the circuit replaced by a classical layer of the same width, trained under the same protocol. The second issue is the data. Public medical imaging datasets often contain duplicated patients or images across training and test partitions and non-clinical cues that correlate with the label; both inflate test accuracy and hide shortcut learning [32,33,34,35,36,37,38]. For the Kaggle brain tumor dataset, duplicate images across partitions have been reported in its first release [39,40,41]. A second release in February 2026 was announced as de-duplicated and class-balanced [9], although candidate near-duplicate pairs across its partitions were subsequently reported [42]. When a test set is leaky, differences of a few percentage points between a quantum and a classical head can reflect which model memorizes duplicates better rather than which generalizes better.

Here we address both issues in a controlled benchmark that extends our earlier four-qubit quantum convolutional network for the same task [22]. Our contributions are as follows:

- **A dataset audit.** We audit version 2 of the Kaggle Brain Tumor MRI Dataset for exact, near-duplicate, mirrored, rotated and augmented copies across partitions, non-semantic shortcuts, heterogeneity of acquisition source and candidate label errors. By matching every image to the original figshare collection [3] and to the SARTAJ collection [7], we recover the patient identity of 4846 tumor images and verify the source and label of every tumor image, and we release leakage-aware splits in which duplicate groups and patients never cross partitions.
- **Matched controls in three regimes.** We compare a six-qubit VQC head with a size-matched classical control, a deeper nonlinear classical control, a classical Fourier surrogate whose function class contains that of the circuit, a bottleneck-only head, a linear probe, a multilayer perceptron and an untrained circuit on frozen features from four ImageNet-pretrained backbones; with end-to-end fine-tuning of the backbone; and in a hybrid network trained from scratch. We also compare a quantum-kernel support vector machine with a classical kernel.
- **Ablations, robustness and shortcut controls.** We vary the number of qubits, circuit depth, entanglement and data re-uploading, measure data efficiency from 1% to 100% of the training data and evaluate trained circuits under depolarizing noise and finite measurement shots. We test the conclusions on head-masked and intensity-standardized images, with the lesion occluded, within a single acquisition source and across sources.
- **Leakage-aware statistics and error decomposition.** We use patient-grouped cross-validation, image-level paired tests, a cluster bootstrap over patient and duplicate groups and an equivalence margin, and we estimate the effect of leakage causally, by training the same models under duplicate-grouped and patient-grouped protocols, and break the test error down by acquisition source.

Section 2 describes the dataset, the audit, the models and the statistical analysis. Section 3 reports the results, and Section 4 discusses their implications for quantum machine learning in medical imaging and the limitations of the study.

## 2. Materials and Methods

### 2.1. Dataset

We used version 2 of the Brain Tumor MRI Dataset curated by Nickparvar and distributed through Kaggle under a CC BY 4.0 license (released on 13 February 2026) [9]. The release was downloaded as the Kaggle archive of the dataset masoudnickparvar/brain-tumor-mri-dataset (version 2) on 28 September 2026; the dataset carries a single, version-independent DOI (10.34740/KAGGLE/DSV/2645886), so we release a manifest of the 7200 file names, file sizes and pixel checksums with which a local copy can be verified (Data Availability Statement). According to the curator, it combines the figshare collection of Cheng et al. [3], the SARTAJ collection [7] and the Br35H collection [8]; the no-tumor images come from Br35H, and glioma images from SARTAJ were replaced by figshare images because of labeling problems. Version 2 contains 7200 two-dimensional MRI slices in JPEG format, 1800 in each of four classes (glioma, meningioma, pituitary tumor and no tumor), distributed as a training partition of 5600 images (1400 per class) and a test partition of 1600 images (400 per class). The curator states that duplicate images were removed and that the overlap between the training and test partitions was eliminated in this version, which replaces the first release (7023 images; 5712 training and 1311 test images) used by most earlier studies. The images vary in size, orientation, pulse sequence and color mode, and no patient identifiers are provided. The figshare collection, in contrast, provides a patient identifier, the tumor type and a manual tumor mask for each of its 3064 T1-weighted contrast-enhanced slices from 233 patients (512 × 512 pixels; slice thickness 6 mm, gap 1 mm) [3]; we downloaded it from figshare (article 1512427) for the provenance analysis below.

### 2.2. Dataset Audit

*Duplicates.* All images were converted to 8-bit grayscale. Exact duplicates were identified by the message-digest algorithm 5 (MD5) checksum of the decoded pixel array. Near-duplicates were identified by a 64-bit perceptual hash (pHash) [43] with a Hamming distance of at most 8 bits, and every candidate pair was confirmed by a normalized cross-correlation (NCC) of at least 0.95 between 64 × 64-pixel thumbnails. Because pHash is not invariant to reflections and rotations, the revised audit hashed every image after each of the eight rotations and reflections of the square and accepted a pair when the smallest Hamming distance over the eight transformations was at most 8 bits and the NCC between the correspondingly transformed thumbnails was at least 0.95. Both thresholds were fixed before the analysis (an eighth of the hash length and a correlation close to that of re-encoded copies); their influence was examined over Hamming distances of 4–14 bits and NCC thresholds of 0.90–0.98 with and without the transformations. To estimate the precision of the detector, a stratified random sample of 130 accepted pairs (40 each of near, mirrored and rotated pairs, half of them close to a threshold and 10 exact pairs) and 40 rejected candidates (NCC 0.90–0.95, or Hamming distance 9–12 with NCC ≥ 0.95) was inspected side by side and classified as the same image (possibly re-encoded, re-windowed, rescaled, mirrored or rotated), another slice of the same examination or an unrelated examination (sheets in Figure S1). Images connected by duplicate pairs were merged into duplicate groups (connected components); groups of ten or more images were inspected visually to detect chains that merge unrelated examinations.

*Augmented copies.* The 203 file names containing “aug” (all meningioma; 100 training and 103 test images) indicate augmented images. Each was matched to its most similar non-augmented image with a perceptual hash that is invariant to the eight rotations and reflections of the square, and it joined that image’s duplicate group when the Hamming distance was at most 10 bits; 202 of the 203 augmented images were linked in this way, and the remaining one was treated as a unique image.

*Provenance and patient identity.* Every image was compared with the 3064 figshare slices, each min–max scaled to 8 bits as described by the figshare curators, by the NCC of 64 × 64-pixel thumbnails under the eight rotations and reflections; an image was assigned to the figshare slice with the highest NCC when it was at least 0.95, and it then inherited that slice’s patient identifier, tumor type and tumor mask. The figshare collection (article 1512427, version 8) and the SARTAJ collection [7] (3264 images in four classes, in training and testing folders) were downloaded on 13 October 2026, and every image was matched to the SARTAJ images in the same way, so that the source of the tumor images not derived from figshare could be identified. The JPEG encoding of every file (quantization tables, chroma subsampling and color mode) was recorded as a fingerprint of the processing pipeline that produced it.

*Shortcut probes.* To measure how much label information is carried by features unrelated to tumor morphology, we trained gradient-boosting classifiers (scikit-learn defaults) on the official training partition and tested them on the official test partition and repeated every probe under the patient-grouped cross-validation described below. The inputs were (i) image width, height and aspect ratio, a 512 × 512 indicator and two color-mode indicators; (ii) the same plus the JPEG file size; (iii) six global intensity statistics (mean, standard deviation, 99th percentile, fraction of dark pixels, mean border intensity and entropy); and (iv) the 64 pixel values of an 8 × 8 thumbnail; (iii) and (iv) were also computed on the standardized images described in Section 2.9. Each probe was also trained to separate tumor from no-tumor images.

*Acquisition source.* For each class and partition, we tabulated native matrix sizes. The figshare images are 512 × 512 pixels, so other matrix sizes indicate other sources; source membership was then verified by the figshare matching and the encoder fingerprints.

*Candidate label errors.* Out-of-fold class probabilities of multilayer-perceptron heads on four frozen backbones (Section 2.5, grouped cross-validation) were averaged and screened with confident learning [44,45]; images whose given label received less than 10% probability while another class received at least 90% were flagged for expert review. For figshare-derived images, the given label was also compared with the tumor type recorded in the figshare collection, which was assigned by the original investigators together with a manual tumor delineation [3].

### 2.3. Evaluation Protocols

**Figure 1:**
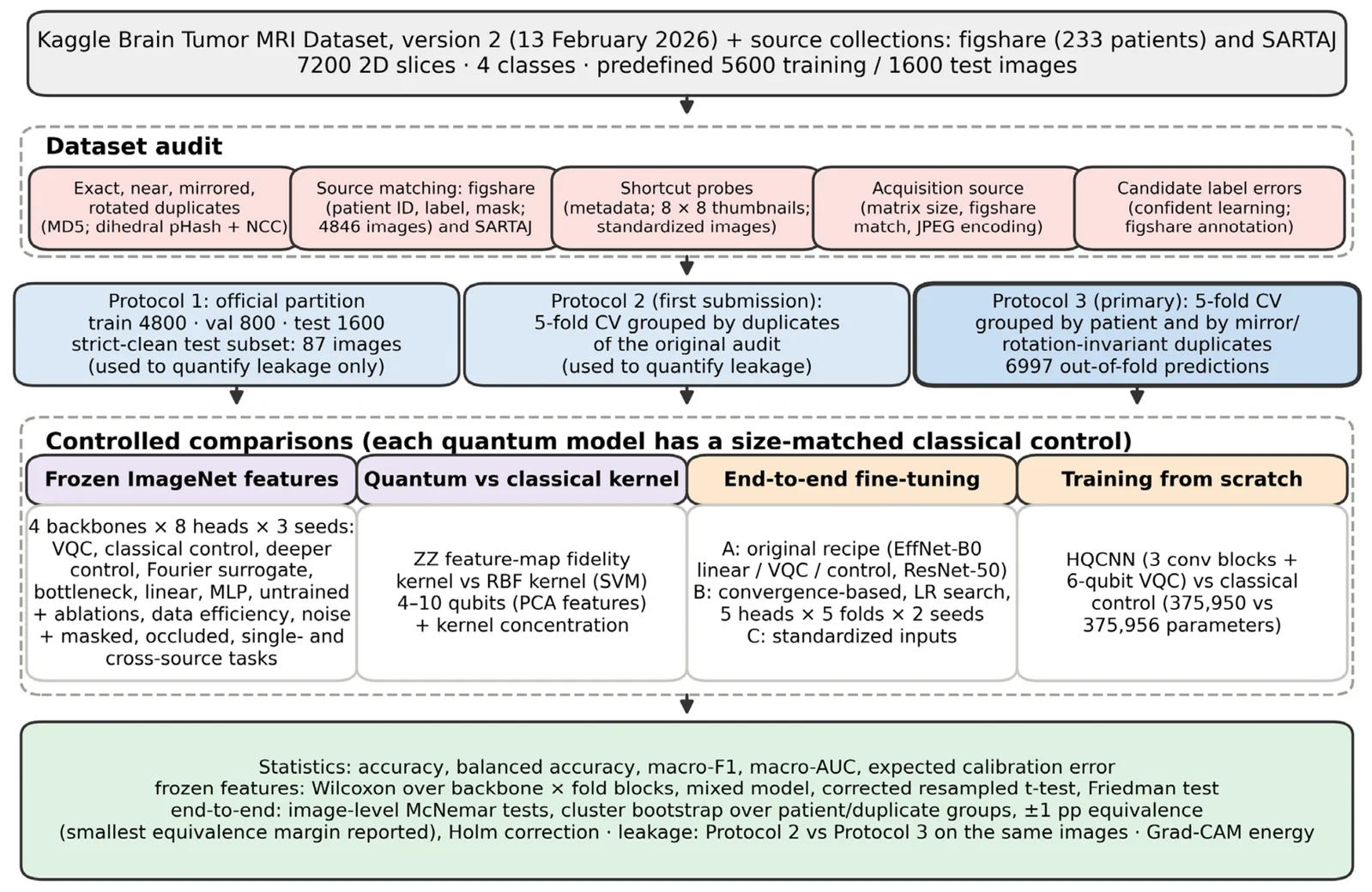
Study design. The dataset was audited for duplicates, augmented copies, shortcuts, acquisition sources and candidate label errors. Patients were recovered from the figshare collection, and three evaluation protocols were defined; Protocol 3 is the primary one. In every regime, each quantum model was compared with a classical control of the same width trained under the same protocol. ECE, expected calibration error; HQCNN, hybrid quantum–classical convolutional neural network; MD5, message-digest algorithm 5; NCC, normalized cross-correlation; pHash, perceptual hash; RBF, radial basis function; VQC, variational quantum circuit.

Three protocols were used (Figure 1). *Protocol 1 (official partition)* keeps the predefined test partition of 1600 images and holds out a validation subset of 800 training images (one seventh, stratified by class and never sharing a duplicate group with the remaining 4800 training images) for learning-rate selection, early stopping and checkpoint selection. Because the revised audit showed that most official test images are linked to the training partition (Section 3.1), Protocol 1 is retained only to quantify leakage and source shift; its *strict-clean subset* consists of the 87 test images that share neither a duplicate group (revised definition) nor a patient with any training image, and the *clean subset* of the first submission (832 images) is kept for comparison. *Protocol 2 (grouped cross-validation, as submitted)* pools both partitions and assigns all non-augmented images to five folds by stratified group *k*-fold splitting, so that a duplicate group of the original audit never spans two folds; augmented images are assigned to the fold of their source image and are used for training only. *Protocol 3 (patient-grouped cross-validation, primary)* uses the same procedure with groups formed by merging the revised duplicate groups with all images of the same figshare patient (754 groups, largest 84 images), so that no figshare patient and no detected mirrored, rotated or re-encoded copy spans two folds. Tumor images from other sources and no-tumor images carry no patient identifiers, so other slices of the same examination may still fall into different folds (Section 4.5). Inside each training fold, a one-seventh group-aware subset serves as the validation set. Each of the 6997 non-augmented images is thus predicted exactly once in each cross-validation protocol; under Protocol 3, it is predicted by a model that has seen neither the image, nor any detected copy of it, nor, for figshare-derived images, any other image of the same patient. All results below refer to Protocol 3 unless stated otherwise; Protocol 2 results of the first submission are reported in Table S7 and used to estimate the effect of leakage.

### 2.4. Variational Quantum Circuit

**Figure 2:**
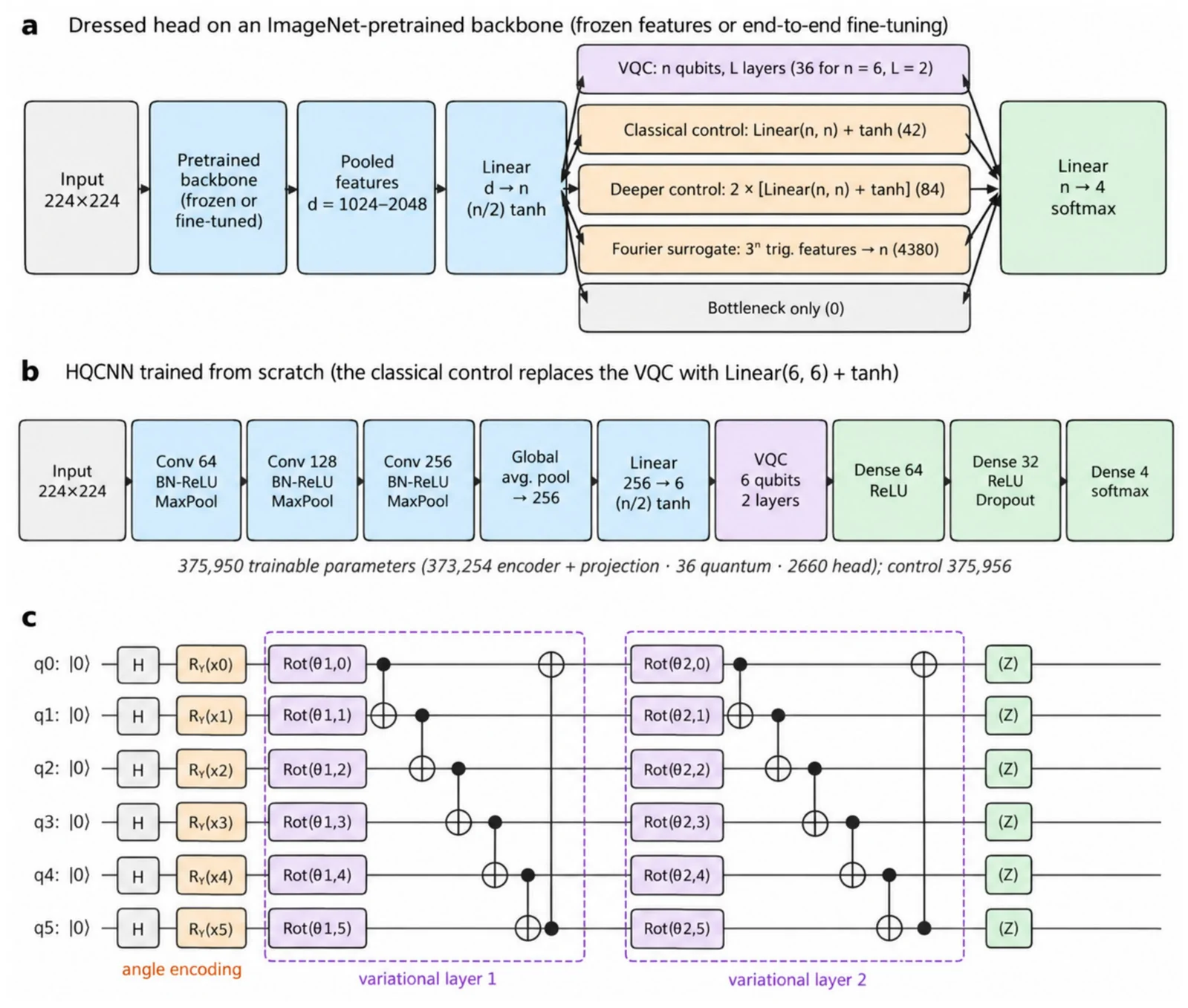
Models. (**a**) Dressed head on ImageNet-pretrained features: a linear map produces *n* angles, which pass through the VQC, a matched classical layer (Linear(*n*, *n*) + tanh) or nothing (bottleneck only) before a linear read-out. The backbone was frozen (Section 2.5) or fine-tuned end to end (Section 2.7). (**b**) Hybrid quantum–classical CNN (HQCNN) trained from scratch (Section 2.8). (**c**) The six-qubit, two-layer circuit: Hadamard and R_Y_ angle encoding, two layers of general single-qubit rotations (Rot) followed by a ring of CNOT gates and measurement of ⟨*Z*⟩ on every qubit. The deeper control and the Fourier surrogate (Section 2.4) replace the middle layer in panel (**a**).

All quantum layers share one design (Figure 2c): a hardware-efficient ansatz [46,47] used as the middle layer of a dressed quantum circuit [13]. A classical linear layer maps a *d*-dimensional feature vector **f** to *n* rotation angles,

(1) $$x=\frac{\pi }{2}tanh(Wf+b)\in {\left(-\pi /2,\pi /2\right)}^{n},$$

which are encoded into *n* qubits by a Hadamard gate followed by R_Y_(*x_i_*) on each qubit. The encoded state is processed by *L* variational layers, each applying a general single-qubit rotation Rot(*α*, *β*, *γ*) = R_Z_(*γ*)R_Y_(*β*)R_Z_(*α*) with three trainable angles to every qubit, followed by a ring of controlled-NOT (CNOT) gates in which qubit *i* controls qubit *i* + 1 (modulo *n*):

(2) $$|\psi (x;\theta )\rangle =U_{ent}R(\theta^{\left(L\right)})\cdots U_{ent}R(\theta^{\left(1\right)}){⨂}_{i=1}^{n}R_{Y}(x_{i})H|0\rangle .$$

The circuit outputs the expectation values *z_i_* = ⟨ψ(**x**; *θ*)|*Z_i_*|ψ(**x**; *θ*)⟩ ∈ [−1, 1] of the Pauli-*Z* operator on each qubit, and a classical linear layer maps them to the four class logits. With *n* = 6 and *L* = 2, the circuit has 36 trainable parameters. Three variants removed the CNOT ring (no entanglement), re-applied the encoding R_Y_(*x_i_*) before every variational layer (data re-uploading [48]) or froze the circuit parameters at their random initialization (untrained VQC). The matched *classical control* replaces the circuit by Linear(*n*, *n*) followed by tanh, which has the same input and output width, a similar number of parameters (42 versus 36 for *n* = 6) and a bounded output, and the *bottleneck-only* head removes the middle layer altogether. Because matching width and parameter count does not match expressive capacity, two further classical middles were added. The *deeper control* stacks two Linear(*n*, *n*) + tanh layers (84 parameters). The *Fourier surrogate* exploits the fact that, when every angle is encoded once, each output *z_i_* of the circuit is a trigonometric polynomial with frequencies in {−1, 0, 1} in each angle [49], that is, a linear combination of the 3^n products ∏_g_i(x_i)_ with *g_i_* ∈ {1, cos *x_i_*, sin *x_i_*}; a linear layer on these 729 features (4380 parameters for *n* = 6) can therefore represent every function computed by circuits of this design, which makes it a classical surrogate in the sense of Schreiber et al. [50].

Circuits were simulated exactly with a batched state-vector simulator written in PyTorch (version 2.14.0), so that the circuit could be trained jointly with the classical layers on a graphics processing unit (GPU; version 2.14.0). Noisy circuits were simulated with density matrices, applying a single-qubit depolarizing channel ρ → (1 − *p*)ρ + (*p*/3)(*X*ρ*X* + *Y*ρ*Y* + *Z*ρ*Z*), so that *p* is the probability of a Pauli error [51], to every qubit after each gate layer (encoding, rotation layer and CNOT ring); the channel thus acts once per qubit and layer, after the CNOT ring as well as after the single-qubit layers. Finite-shot estimates of ⟨*Z_i_*⟩ were obtained by sampling *S* measurement outcomes from the exact outcome probabilities. For orientation, reported average error rates of current processors are about 10^−5^–10^−3^ for single-qubit gates, 2 × 10^−3^–6 × 10^−3^ for two-qubit gates and 2 × 10^−3^–4 × 10^−2^ for readout [52,53]; our uniform channel with *p* = 0.001–0.01 per qubit and layer therefore spans the range of current hardware, but it omits crosstalk, readout error, limited connectivity and gate decomposition, and the simulation timings say nothing about execution time on a quantum processor. Expectation values agreed with PennyLane’s default.qubit and default.mixed simulators [54] to within 10^−6^ for noise-free and noisy circuits (self-tests are included in the released code).

### 2.5. Frozen-Feature Experiments

Four ImageNet-pretrained backbones [55] from the timm library [56] were used as fixed feature extractors: EfficientNet-B0 [57], DenseNet-121 [58], ResNet-50 [59] and MobileNetV2 [60]. Images were converted to grayscale and replicated to three channels, resized to 256 × 256 pixels, center-cropped to 224 × 224 pixels and normalized with the ImageNet statistics. The globally pooled penultimate activations (*d* = 1280, 1024, 2048 and 1280, respectively) were standardized with the mean and standard deviation of each training partition. Eight heads were trained on these features (Figure 2a): the six-qubit, two-layer VQC head; the matched classical control; the deeper control; the Fourier surrogate; the bottleneck-only head; a linear probe; a multilayer perceptron (MLP) with 256 hidden units, rectified linear unit (ReLU) activation and dropout of 0.3; and the untrained VQC head. Every head was trained with Adam [61] (weight decay 10^−4^, batch size 128, at most 80 epochs); the learning rate was halved after five consecutive epochs without improvement of the validation loss, training stopped after ten epochs without improvement, and the checkpoint with the lowest validation loss was evaluated. The initial learning rate was selected for each run from {10^−3^, 3 × 10^−3^, 10^−2^} on the validation loss. The main comparison used all four backbones under Protocol 3 with three seeds (0–2) per fold (one for the untrained VQC), giving 20 backbone–fold blocks with three runs each.

Ablations used EfficientNet-B0 features and Protocol 3. We varied the bottleneck width *n* ∈ {2, 4, 6, 8, 10} (the number of qubits for the VQC head, each with its classical control and bottleneck-only counterpart), the number of variational layers *L* ∈ {1, 2, 4, 6} and the no-entanglement and re-uploading variants. For data efficiency, the four trainable reference heads (VQC head, classical control, linear probe and MLP) were trained on 1%, 2%, 5%, 10%, 25% or 50% of each training fold, subsampled within each class by whole patient and duplicate groups, with the validation set unchanged. Noise robustness was assessed by evaluating the trained VQC heads (two to six qubits, one to six layers; all trained without noise) on each test fold with depolarizing probabilities *p* ∈ {0.001, 0.003, 0.01, 0.03, 0.1}, with *S* ∈ {16, 64, 256, 1024, 4096} shots and with *p* = 0.01 combined with 1024 shots.

### 2.6. Quantum and Classical Kernels

We compared a quantum-kernel support vector machine (SVM) with a classical one on the same inputs under Protocol 3. EfficientNet-B0 features were standardized, reduced to n ∈ {4, 6, 8, 10} principal components fitted on each training partition and min–max scaled to [0, 1] with the training range. For the quantum kernel, each vector was multiplied by *πγ* and encoded with the ZZ feature map of Havlíček et al. [62], that is, two repetitions of a layer of Hadamard gates followed by a diagonal unitary with single-qubit phases *x_i_* and pairwise phases (*π* − *x_i_*)(*π* − *x_j_*) on all qubit pairs. The kernel was the state fidelity *k*(**x**, **x**′) = |⟨ψ(**x**)|ψ(**x**′)⟩|^2^, computed exactly from state vectors [63]. The classical kernel was the radial basis function (RBF) kernel on the same *n* components. For each kernel, the bandwidth *γ* (quantum, the factor γ above: {0.03, 0.1, 0.3, 1}; RBF: {0.1, 1, 10, 30, 100}, divided by *n* times the variance of the scaled training features) and the regularization constant *C* ∈ {1, 10, 100} were selected on validation accuracy. As a diagnostic of exponential kernel concentration [64], we report the variance of the off-diagonal entries of the training kernel matrix, for the selected bandwidth and, on 2000 randomly chosen training images per fold, for every bandwidth.

### 2.7. End-to-End Fine-Tuning

Six ImageNet-pretrained backbones from timm were fine-tuned end to end with a linear classification head: EfficientNet-B0, ResNet-50, DenseNet-121, MobileNetV2, ConvNeXt-Tiny [65] and ViT-Small/16 [66]. EfficientNet-B0 was also fine-tuned with the dressed six-qubit VQC head (*n* = 6, *L* = 2) and with its matched classical control, so that the backbone could adapt to the quantum layer. In the first submission (Protocols 1 and 2), training used AdamW [67] (weight decay 0.05) with a one-cycle learning-rate schedule [68] (10% warm-up; peak learning rate 10^−3^ for the head and 3 × 10^−4^ for the backbone), batch size 32, label smoothing of 0.05 [69] and mixed precision, with the circuit always simulated in 32-bit precision. Training ran for at most 30 epochs and stopped after six epochs without improvement of the validation loss. Training images were augmented with random resized crops (scale 0.8–1.0, aspect ratio 0.9–1.1), horizontal flips, rotations of up to 10° and brightness and contrast jitter of 0.15. All images were converted to grayscale, replicated to three channels and resized to 224 × 224 pixels. Each model was trained once per cross-validation fold (seed 0) and three times on the official partition (seeds 0–2), giving 80 training runs.

The revision repeated the end-to-end experiments under Protocol 3 in three blocks. In block A, the recipe above was applied unchanged to EfficientNet-B0 with the linear, VQC and classical-control heads, ResNet-50, the HQCNN and its control (five folds, seed 0), so that the comparison with the submitted Protocol 2 runs isolates the effect of the splits. In block B, the five EfficientNet-B0 heads (linear, VQC, classical control, deeper control and Fourier surrogate) were trained with a convergence-based recipe: a two-epoch linear warm-up, halving of the learning rate after three epochs without improvement of the validation loss, early stopping after ten such epochs and a maximum of 80 epochs. For each head, the peak head learning rate was selected from {3 × 10^−4^, 10^−3^, 3 × 10^−3^} (backbone, 0.3 times the head rate) on the inner validation set of the first fold, and the selected rate was used for five folds and two seeds (ten runs per head). In block C, the linear, VQC and classical-control heads were trained with the block-A recipe on the standardized images of Section 2.9 (five folds).

### 2.8. Hybrid Network Trained from Scratch

To test the configuration used in most earlier work, including ours [22], we also trained a hybrid quantum–classical CNN (HQCNN) from random initialization (Figure 2b). Its encoder has three blocks of 3 × 3 convolution (64, 128 and 256 channels), batch normalization, ReLU and 2 × 2 max pooling, followed by global average pooling and a linear projection to six angles as in Equation (1). The six-qubit, two-layer VQC is followed by a classical head with dense layers of 64 and 32 units (ReLU; dropout of 0.5 before the output layer) and a four-unit output. The HQCNN has 375,950 trainable parameters, 36 of them quantum; its classical control, with Linear(6, 6) + tanh in place of the circuit, has 375,956. Both were trained with the settings of Section 2.7, except that all parameters used a peak learning rate of 10^−3^.

### 2.9. Metrics and Statistical Analysis

We report accuracy, balanced accuracy (the unweighted mean of the four class recalls), the macro-averaged F1 score (the unweighted mean of the four one-versus-rest F1 scores), the macro-averaged one-versus-rest area under the receiver operating characteristic curve (AUC; the unweighted mean of the four one-versus-rest AUCs computed from the predicted class probabilities) and the expected calibration error (ECE) with 15 equal-width confidence bins on the maximum predicted probability, weighted by the number of images per bin [70]. Class-specific sensitivity, specificity, F1 score and AUC, with confusion matrices, are reported in the Supplementary Materials. For the frozen-feature heads, each backbone–fold combination is a block (4 × 5 = 20 blocks), and accuracies were averaged over the three seeds within each block. Paired differences between heads were tested with the Wilcoxon signed-rank test [71], with bootstrap 95% confidence intervals (CIs) of the mean difference [72], and all heads were compared with the Friedman test [73,74]. Because blocks share images and overlapping training sets, we also fitted a linear mixed model to the 60 run-level differences with random intercepts for backbone and for fold within backbone, applied the variance-corrected resampled *t*-test for repeated cross-validation to each backbone [75,76] and computed an image-level cluster bootstrap that resamples patient and duplicate groups.

For the end-to-end models, five folds are too few for fold-level tests to be informative (the smallest attainable two-sided Wilcoxon *p*-value is 0.0625), so inference was made at the image level. Out-of-fold predictions were pooled over the five folds, and two models were compared with the exact McNemar test [77,78]. Because images in the same patient or duplicate group are not independent, 95% CIs of accuracies and of paired differences were obtained with a cluster bootstrap that resamples these groups (5000 resamples; 2000 for class-specific metrics and for the subsets of the leakage analysis) [79]. Two models were declared equivalent when the 90% CI of their paired difference lay entirely within ±1 percentage point (pp), which corresponds to two one-sided tests at *α* = 0.05 [80]. The margin of 1 pp was chosen because it is smaller than the spread between the six fine-tuned backbones in the first submission (1.2 pp) and corresponds to about 70 of 6997 images; because any margin is a convention [81], we also report the smallest margin at which equivalence would be declared (the largest absolute bound of the 90% CI). The VQC-versus-classical-control contrast was the primary comparison in each regime and was not adjusted; secondary contrasts were adjusted with the Holm procedure [82]. Image-level tests treat the trained models as fixed and do not capture run-to-run training variability, so fold-level and seed-level differences are reported alongside them; for block B, accuracies and their CIs were averaged over the two seeds of each image, McNemar tests compared the two-seed ensembles (averaged class probabilities) and the ten fold–seed differences were also analyzed with the corrected resampled *t*-test and a mixed model with a random intercept per fold.

*Leakage and source effects.* The effect of leakage was estimated by training identical models on identical images under Protocol 2 (duplicate groups of the original audit) and Protocol 3 (patients and revised duplicate groups) and comparing the predictions for each image; the difference is reported for images that had a detected copy, or only another image of the same patient, in a Protocol 2 training fold, and for unlinked images, with a cluster-bootstrap CI. For each model trained under Protocol 1, we compared accuracy on the test images outside the clean subset (“leaked”) with accuracy on the clean images, computed a class-standardized difference (the unweighted mean over classes of the within-class differences) with a cluster-bootstrap CI (2000 resamples) and repeated it after excluding test images from an acquisition source that is absent from the training partition of their class (Section 3.1). For reference, the same analysis was applied to one-nearest-neighbor classifiers on frozen features (cosine similarity), which can only succeed by retrieving similar training images.

*Shortcut and source controls.* Three experiments tested whether accuracy reflects tumor morphology. First, every image was standardized: a head mask was obtained by Gaussian smoothing, a permissive Otsu threshold, selection of the largest connected component, morphological closing and hole filling; the background was set to zero, and the image was cropped to the mask, padded to a square, rescaled to the 1st–99th intensity percentile inside the mask and resized to 256 × 256 pixels (examples in Figure S5). This removes the background, borders, overlays, matrix size and intensity scale, although not the skull, because reliable skull stripping is not available for two-dimensional slices in three planes; frozen-feature heads and fine-tuned models (block C) were trained and tested on the standardized images under Protocol 3. Second, the tumor of every figshare-derived test image was occluded by filling its manual mask, dilated by 8 pixels, with the median intensity of the remaining head, and the heads trained on unoccluded images were evaluated on the occluded images of their test fold. Because the occluded region preserves the location, size and shape of the lesion, a logistic regression on the manual mask alone, downsampled to 16 × 16 pixels, was trained and tested on the same folds. Third, two source-controlled tasks were defined: a three-class tumor task restricted to figshare-derived images (one source; patient-grouped five-fold cross-validation) and a cross-source task in which heads trained on all figshare-derived tumor images (with a patient-grouped validation set) were tested on the tumor images from other sources, excluding any image linked to a figshare image by the duplicate graph.

*Explanations.* Gradient-weighted class activation maps (Grad-CAM) [83] were computed for the predicted class at the last convolutional layer of five models (ResNet-50, EfficientNet-B0 with the linear, classical-control and VQC heads and the HQCNN) trained under Protocol 3 (block A) for all test images of the first fold. We report the share of the Grad-CAM energy that falls inside the head mask and, for figshare-derived images, inside the manual tumor mask, together with the area of the mask; 16 test images (four per class, chosen at random before any prediction was inspected) are shown in Section 3.9.

### 2.10. Implementation

All code was written in Python with PyTorch [84], timm and scikit-learn; PennyLane (version 0.45.1) was used only to verify the simulator. The frozen-feature, kernel, probe and noise experiments were run on a CPU (two cores of an Intel Xeon processor at 2.1 GHz; PyTorch 2.14.0, timm 1.0.30, scikit-learn 1.8.0). The end-to-end models were trained on one NVIDIA GeForce RTX 3090 GPU (24 GB; driver 580.126.09, CUDA 13.0, cuDNN 9.24, Python 3.12.12, PyTorch 2.14.0, timm 1.0.30; host CPU Intel Xeon E5-2686 v4), and these versions were recorded for every run; the runs of the first submission used the same recipe. Training times include data loading and augmentation. Latency was measured as the median of 50 single-image forward passes (batch size 1) after 20 warm-up passes, with the input tensor already on the GPU, so it excludes JPEG decoding, resizing and the transfer to the GPU but includes the simulation of the circuit. All circuits were simulated on classical hardware; no experiment was run on a quantum processor. Generative artificial intelligence (Claude, Anthropic; Opus model family) was used to write and debug analysis code and to run the statistical analyses under the authors’ direction; the authors specified every analysis, checked the code and the results and release the complete code (Data Availability Statement).

## 3. Results

### 3.1. Dataset Audit

**Figure 3:**
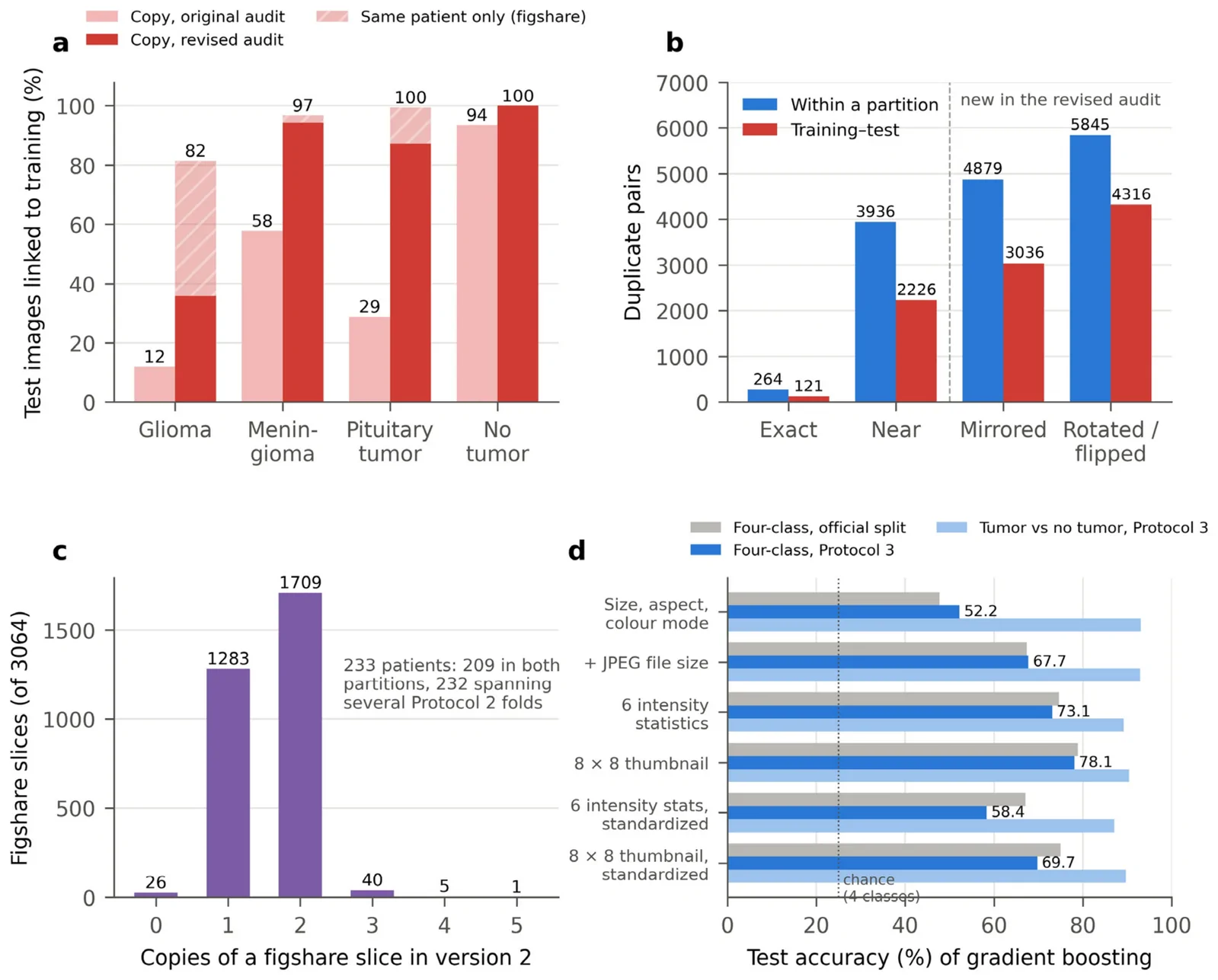
Audit of the Brain Tumor MRI Dataset, version 2. (**a**) Test images that share a duplicate group with a training image, by class, for the original and the mirror- and rotation-invariant detector, and test images that share only a patient with the training partition. (**b**) Duplicate pairs by type (exact, near, mirrored, rotated) within and across partitions. (**c**) Number of copies of each figshare slice in version 2, and figshare patients by partition. (**d**) Test accuracy of gradient-boosting classifiers trained on features that contain no tumor morphology, under the official split and under Protocol 3, for the original and the standardized images.

The audit contradicted the description of version 2 as free of duplicates and of training–test overlap (Table 1, Figure 3). With the detector of the first submission, duplicate detection found 385 exact and 6162 near-duplicate pairs; 2347 pairs crossed the training–test boundary, 10 had conflicting labels (six of them across partitions), and 757 test images (665 non-augmented images, 114 of them pixel-identical to a training image and 92 augmented images) shared a duplicate group with a training image. The mirror- and rotation-invariant detector of the revision found 18,076 further pairs: 7915 mirrored and 10,161 rotated or flipped copies, for 24,623 pairs in total, 9699 of them across partitions (Figure 3b). Mirrored copies occurred in all four classes, whereas the rotated copies were almost all no-tumor images (10,147 of 10,161 pairs). With these pairs, 1168 of the 1497 non-augmented test images (78%) and 101 of the 103 augmented test images, together 1269 of the 1600 test images (79%), shared a duplicate group with a training image (Figure 3a); only 329 test images were clean under the revised definition, and none of them showed no tumor. The pairs formed 1821 duplicate groups containing 5895 of the 7200 images; the largest group consisted of 83 no-tumor images, all processed copies of one axial slice.

All 130 sampled accepted pairs showed the same image (re-encoded, re-windowed, rescaled, mirrored or rotated) or another slice of the same examination, and none joined unrelated examinations (precision ≥ 97.2%, lower one-sided 97.5% Clopper–Pearson bound; Figure S1). Most of the 40 rejected candidates were also copies or neighboring slices of the same examination, so the detector errs towards missing rather than inventing duplicates; among the sampled pairs in which both images were figshare-derived, all 26 accepted pairs and 23 of 26 rejected candidates belonged to the same patient. Once reflections and rotations were included, the number of linked non-augmented test images was stable across thresholds (1086–1174 for NCC thresholds of 0.95–0.98 and Hamming distances of 4–14 bits; Table S8), whereas the original, non-invariant detector linked 467–692 images over the same range.

The figshare matching explained most of these copies (Figure 3c). Of the 5400 tumor images, 4846 (all 1724 glioma images of 512 × 512 pixels, 1378 meningioma and 1744 pituitary tumor images) are copies of 3038 of the 3064 figshare slices from all 233 figshare patients, and 1755 slices occur two to five times (3563 images), typically once as a grayscale JPEG that is mirrored relative to the original slice and once as an unmirrored RGB JPEG with different quantization tables (Table S9). Consequently, 209 of the 233 patients appear in both partitions, 915 of the 917 figshare-derived test images have at least one image of the same patient in training and 242 test images that the duplicate detector did not link to training share a patient with it. Only 87 test images are linked to the training partition neither by a copy nor by a patient (the strict-clean subset): 74 glioma, 11 meningioma and two pituitary tumor images. Thirteen images that version 2 labels as pituitary tumor, all from two figshare patients, are labeled meningioma in the figshare annotation.

Non-semantic features carried much of the label (Figure 3d). A classifier given only image size, aspect ratio and color mode separated tumor from no-tumor images with 91.8% accuracy (majority-class baseline, 75%), and one given only an 8 × 8-pixel thumbnail reached 78.9% four-class accuracy, against 25% by chance. These probes were hardly affected by leakage: under Protocol 3, they reached 93.0% and 78.1%, respectively. On the standardized images, which no longer carry matrix size, borders or intensity scale, an 8 × 8 thumbnail still gave 69.7% four-class accuracy and six intensity statistics 58.4% under Protocol 3, compared with 73.1% for the intensity statistics of the original images. The acquisition metadata show why (Table 1): 99% of the no-tumor images, but only 0–29% of the tumor images in either partition, are not 512 × 512 pixels.

The glioma class revealed a second, subtler problem. All 1400 training glioma images are 512 × 512 pixels, the format of the figshare collection, but 76 of the 400 test glioma images (19%) come in 61 other matrix sizes. None of these 76 images matches any figshare slice (highest NCC 0.89, against NCC ≥ 0.999 for every in-source glioma image), and all 76 share one JPEG encoding (RGB with full-resolution chroma and a particular quantization table) that occurs in none of the 1400 training glioma images; it occurs otherwise in 156 meningioma and pituitary tumor images and 356 no-tumor images from other sources and in 27 figshare-derived images (18 test gliomas and nine training pituitary tumors; Table S9). These images are visually heterogeneous and include several pulse sequences and imaging planes. The SARTAJ matching identified their source: all 76 are pixel copies (NCC ≥ 0.99999 under the eight rotations and reflections) of images in the *Testing/glioma_tumor* folder of the SARTAJ collection, that is, of the SARTAJ glioma images that the curator describes as replaced because of labeling problems. More generally, every one of the 382 non-augmented tumor images that do not derive from figshare is a copy of a SARTAJ image with the same tumor label, and 1365 of the 1800 no-tumor images match SARTAJ no-tumor images (SARTAJ itself contains many figshare slices). All 76 images are stored in red–green–blue (RGB) mode, and nine of them share a matrix size with training images of other classes. Because the training glioma class contains no image from this source, these 76 test images measure generalization to a new source rather than to new patients; we call them *out-of-source* glioma images. Of the 76, 74 are in the strict-clean subset.

Confident learning flagged 28 of the 6997 non-augmented images as candidate label errors; the most frequent patterns were images labeled glioma that the models assigned to meningioma (8), meningioma assigned to pituitary tumor (6) and pituitary tumor assigned to meningioma (6); one image labeled no tumor appears to be a computed tomography slice (Figure S2). For the 19 candidates that are figshare-derived, the figshare annotation agreed with the given label in 15 cases and supported the suggested label in four, all of them among the 13 pituitary-labeled images that figshare labels meningioma; the remaining nine candidates, including three of the out-of-source glioma images, could not be checked against an independent annotation. These are candidates for radiologist review, not confirmed errors, and they were kept in all analyses.

### 3.2. Frozen Features: The Quantum Head Versus Its Controls

**Figure 4:**
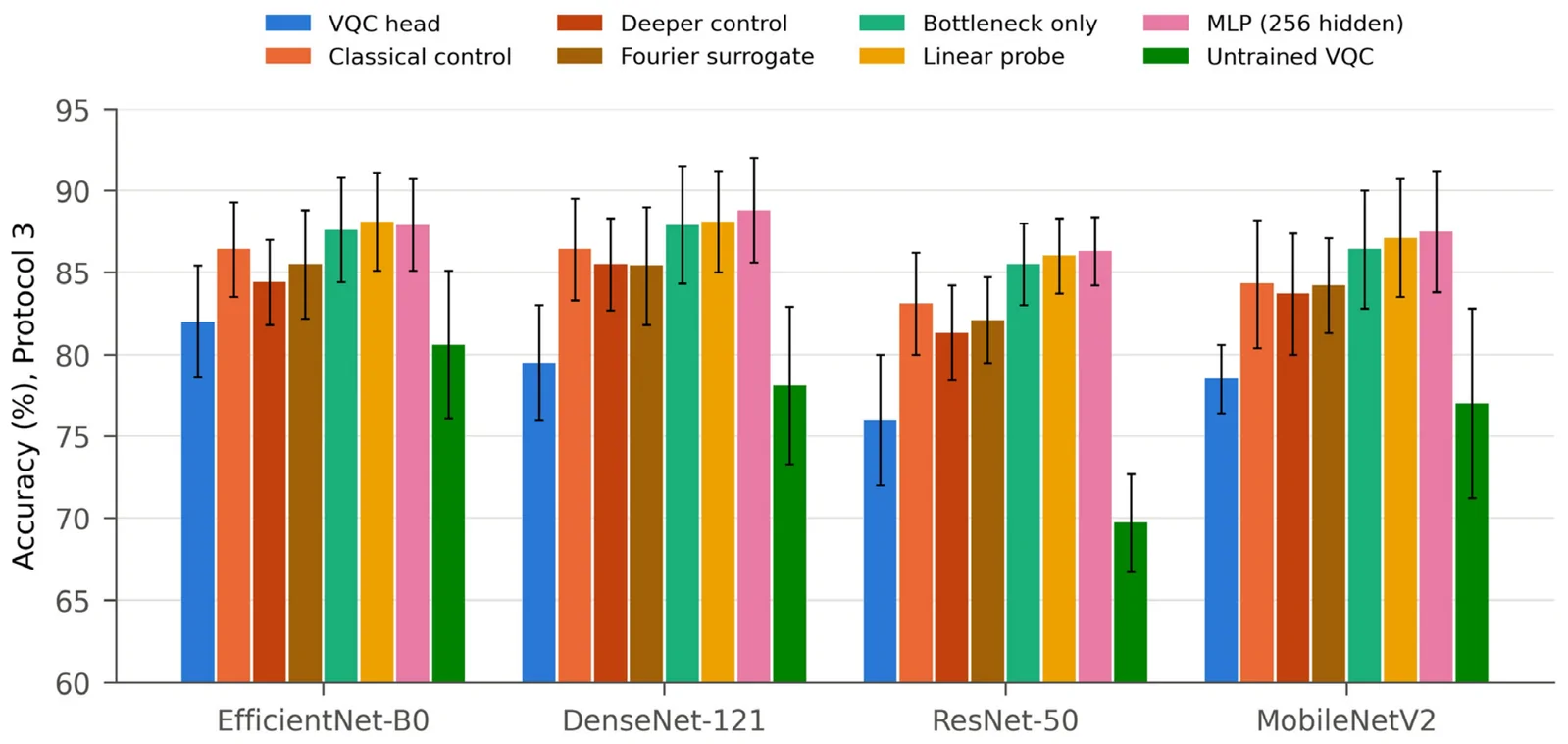
Frozen-feature benchmark: accuracy of eight heads on four ImageNet-pretrained backbones (Protocol 3; bars, mean over folds of the seed-averaged accuracy; error bars, standard deviation over the five folds).

With frozen features, the VQC head was the least accurate trained head on every backbone (Table 2, Figure 4). Across the 20 backbone–fold blocks of Protocol 3, averaged over three seeds, it was 6.0 pp less accurate than the matched classical control (95% CI −6.8 to −5.2; Wilcoxon *p* = 1.9 × 10^−6^) and less accurate in all 20 blocks. Because this *p*-value is the smallest that 20 blocks can produce, it measures the consistency of the sign, not the size of the effect; the size was confirmed by a linear mixed model of the 60 runs with random effects for backbone and fold (−6.0 pp; 95% CI −7.0 to −5.1) and by the image-level cluster bootstrap over patient and duplicate groups (−6.0 pp; −6.6 to −5.5). The variance-corrected resampled *t*-test confirmed the deficit for DenseNet-121, EfficientNet-B0 and ResNet-50 (*p* = 2.9 × 10^−4^ to 5.0 × 10^−3^) but not for MobileNetV2 (−5.8 pp; *p* = 0.075; Table S12). The VQC head was also less accurate than the deeper control (−4.7 pp; −5.6 to −3.7) and than the Fourier surrogate (−5.3 pp; −6.2 to −4.4), whose function class contains that of the circuit, in all 20 blocks in both cases. It was 7.8, 8.3 and 8.6 pp less accurate than the bottleneck-only head, the linear probe and the MLP (each *p* = 1.9 × 10^−6^), respectively. Training the circuit parameters helped only modestly: the trained VQC was 2.7 pp more accurate than the same circuit left at its random initialization (95% CI 1.1 to 4.2; *p* = 0.0083; better in 16 of 20 blocks). The Friedman test rejected equal accuracy of the eight heads (*p* = 9.2 × 10^−25^). The size of the deficit depended on the backbone, from 4.4 pp for EfficientNet-B0 (82.0% versus 86.4%) to 7.1 pp for ResNet-50 (76.0% versus 83.1%). Compared with Protocol 2 of the first submission, every head lost accuracy (Section 3.8), and the deficit of the VQC head relative to its control widened from 4.4 to 6.0 pp. All metrics are listed in Table S1.

### 3.3. Ablations: Qubits, Depth, Entanglement and Re-Uploading

**Figure 5:**
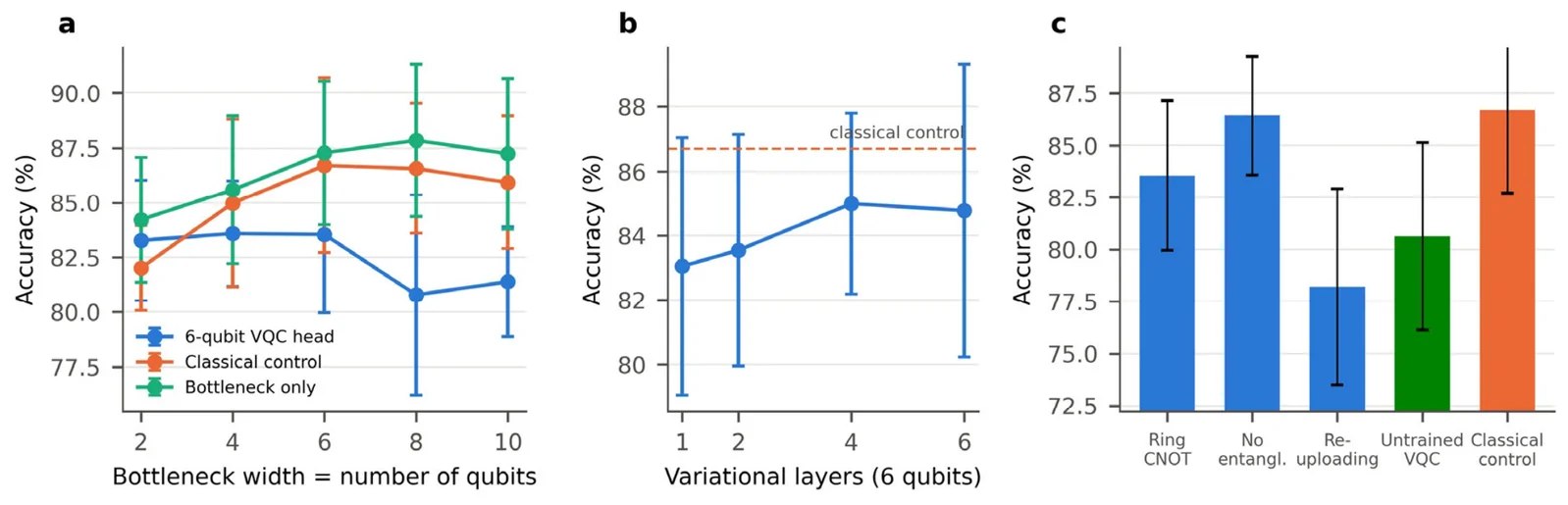
Circuit ablations on frozen EfficientNet-B0 features (Protocol 3; mean ± SD over folds). (**a**) Bottleneck width, which equals the number of qubits for the VQC head. (**b**) Number of variational layers (six qubits); dashed line, classical control. (**c**) Circuit variants and controls (six qubits, two layers).

Ablations on EfficientNet-B0 features did not reveal a configuration in which the circuit helped (Figure 5; Table S2). Increasing the bottleneck width from two to eight improved the classical control from 82.0% to 86.6% and the bottleneck-only head from 84.2% to 87.9%, whereas the VQC head stayed between 83.3% and 83.6% with two to six qubits and fell to 80.8% with eight and 81.4% with ten qubits; it was more accurate than its control only with two qubits (+1.3 pp; better in three of five folds), where the classical control was at its least accurate (82.0%) and both were below the bottleneck-only head (84.2%), and less accurate with every wider bottleneck (−1.4, −3.2, −5.8, −4.5 pp for 4, 6, 8 and 10 qubits). Adding variational layers improved accuracy modestly, from 83.0% with one layer to 85.0% with four (+1.4 pp over two layers; better in four of five folds) and 84.8% with six, all below the 86.7% of the classical control. Removing all CNOT gates raised accuracy from 83.5% to 86.4% (+2.9 pp; better in four of five folds), level with the classical control (−0.3 pp; better in two of five folds), whereas data re-uploading lowered it to 78.2% (−5.3 pp; worse in five of five folds). On the CPU, training a head took 11 s per run with six qubits and 141 s with ten, against 1 s for the six-unit classical control (Table S2).

### 3.4. Data Efficiency and Noise

**Figure 6:**
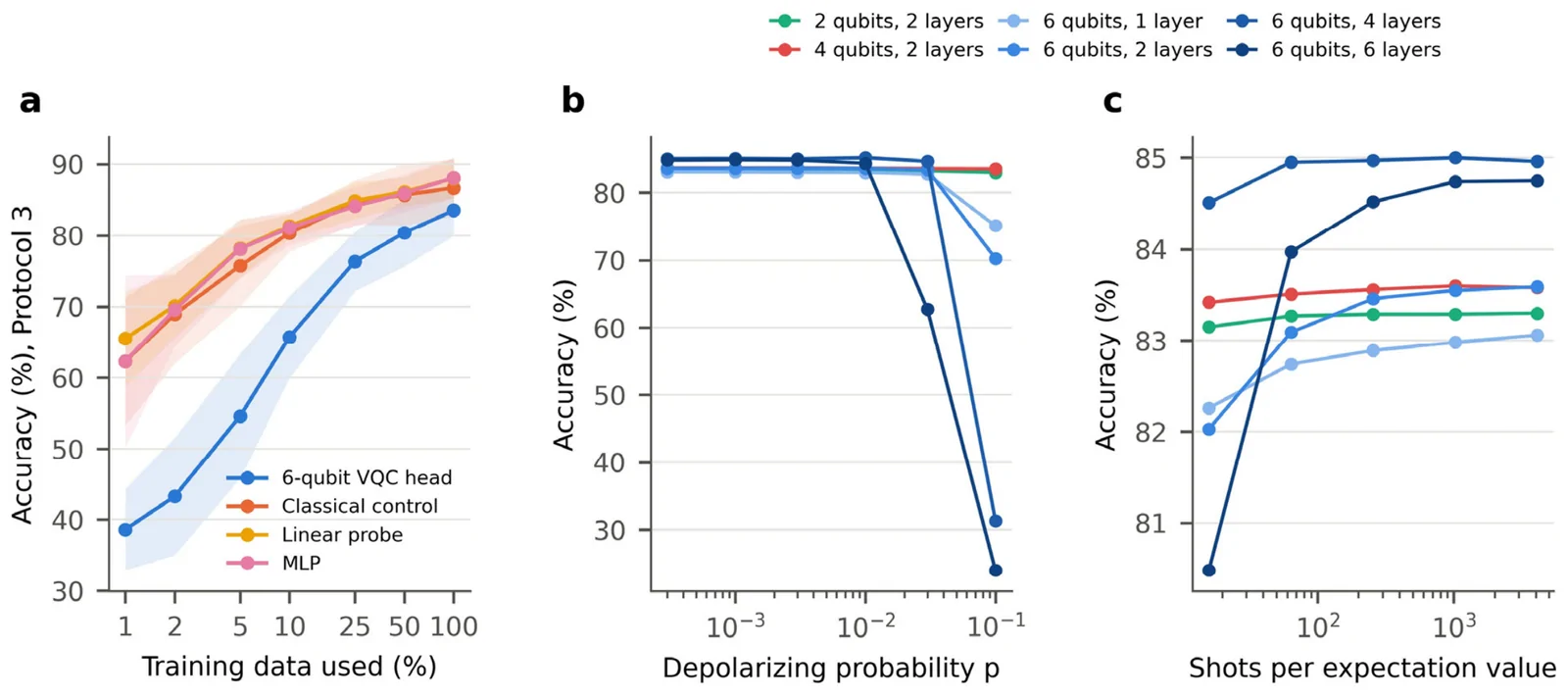
Data efficiency and noise robustness (frozen EfficientNet-B0 features, Protocol 3). (**a**) Accuracy versus the fraction of training data, subsampled by whole patient and duplicate groups (shaded, ±1 SD over folds). (**b**) Trained VQC heads evaluated with a single-qubit depolarizing channel after every gate layer (*p* = 0 is plotted at 3 × 10^−4^). (**c**) Finite-shot estimation of the expectation values. The legend applies to (**b**,**c**), which show means over folds.

The VQC head was least competitive where a compact quantum model might be expected to help most, with few training images (Figure 6a). With a nominal 1% of each training fold (115 images on average, because whole groups were sampled), it reached 38.6%, compared with 62.3% for the classical control, 65.5% for the linear probe and 62.3% for the MLP; the gap to the classical control was 8.3 pp with 25% of the data and 3.2 pp with all of it (Table S3).

The trained circuits tolerated moderate hardware noise (Figure 6b,c). For the six-qubit, two-layer head, accuracy was 83.3% at a depolarizing probability of 0.03 per qubit and layer, against 83.5% without noise and 70.2% at *p* = 0.1. Deeper circuits collapsed at *p* = 0.1 (31.3% with four and 24.0% with six layers; chance, 25%), and the six-layer circuit lost 22.1 pp at *p* = 0.03. With 16 shots per expectation value, the six-qubit, two-layer head lost 1.5 pp, and with 1024 shots, alone or combined with *p* = 0.01, it was within 0.1 pp of the noise-free circuit. Noise therefore does not explain the gap to the classical controls, which is already present in noise-free simulation (Table S4).

### 3.5. Quantum Versus Classical Kernels

**Figure 7:**
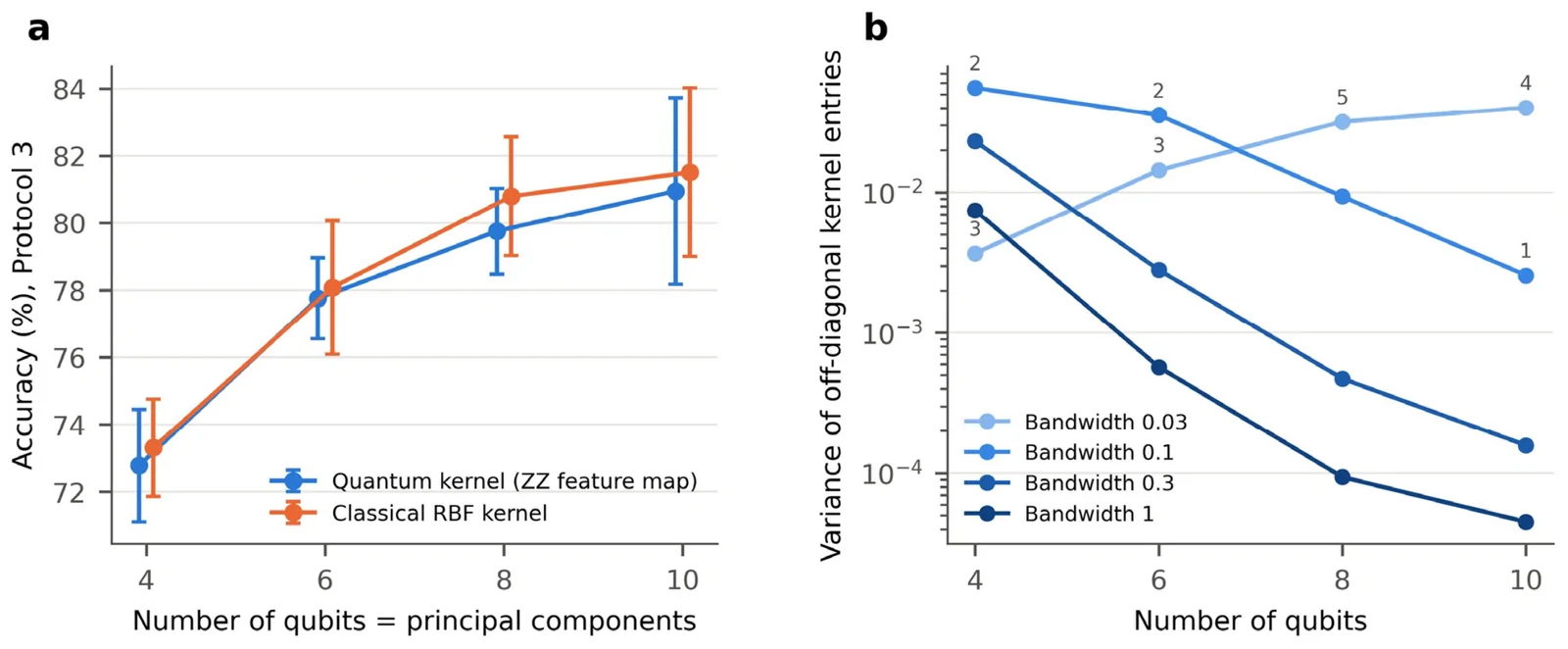
Quantum versus classical kernels on principal components of frozen EfficientNet-B0 features. (**a**) Accuracy of support vector machines with the ZZ feature-map fidelity kernel or the RBF kernel (Protocol 3; mean ± SD). (**b**) Variance of the off-diagonal entries of the quantum training kernel matrix at each fixed bandwidth (mean over folds; 2000 training images per fold). Numbers above the points give how many of the five folds selected that bandwidth on validation accuracy.

The quantum kernel was slightly less accurate than the RBF kernel with every number of principal components (Figure 7a): 72.8% versus 73.3% with four, 77.7% versus 78.1% with six, 79.7% versus 80.8% with eight and 80.9% versus 81.5% with ten components (ZZ kernel better in one, two, zero and three of five folds, respectively). At every fixed bandwidth of 0.1 or more, the variance of the off-diagonal kernel entries fell steeply with the number of qubits, for example from 5.6 × 10^−2^ with four qubits to 2.6 × 10^−3^ with ten at a bandwidth of 0.1 and from 7.4 × 10^−3^ to 4.5 × 10^−5^ at a bandwidth of 1, which is the concentration expected for fidelity kernels [64] (Figure 7b). Tuning the bandwidth is known to counteract this concentration [85], and validation selected the smallest bandwidth, 0.03, in 15 of the 20 fold–width combinations, including all five folds with eight qubits; at this bandwidth, the kernel was nearly constant with few qubits (mean off-diagonal entry 0.92 with four qubits), and its variance rose with the number of qubits. Both kernel machines remained well below the linear probe on the full 1280-dimensional features (88.0%), so the dimensionality reduction required to load features into a small circuit, not the kernel itself, limited accuracy (Table S5).

### 3.6. Shortcut and Source Controls

Neither the shortcut controls nor the single-source task changed the comparison between the circuit and its classical control (Table 3). On the standardized images, from which background, borders, matrix size and intensity scale had been removed, frozen-feature accuracy changed by −1.0 to −0.3 pp depending on the head, and the VQC head remained 6.7 pp less accurate than its control (95% CI −8.7 to −4.8; better in 1 of 20 blocks). After fine-tuning on standardized images (block C), accuracy was within 0.6 pp of that on the original images for all three heads, and the VQC head was 1.1 pp less accurate than its control (95% CI −2.1 to −0.2). Standardization did not remove the source signal, however: an 8 × 8 thumbnail of the standardized image still gave 69.7% four-class accuracy under Protocol 3 (Section 3.1), because coarse anatomy and the imaging plane survive head masking.

Occluding the tumor changed the predictions of the frozen-feature heads little. On the figshare-derived tumor images of the test folds, replacing the tumor with the median head intensity lowered the accuracy of the classical control from 85.6% to 83.0%, that of the linear probe from 87.3% to 84.7% and that of the VQC head from 80.6% to 78.9% (mean over four backbones; drops of 0.0–3.6 pp for all heads and backbones), and the share of tumors classified as no tumor barely changed (0.2–2.2% after occlusion, averaged over backbones, against 0.2–2.1% before). The occluded region still shows the location, size and shape of the lesion, and the mask alone predicted the tumor class with 61.4% accuracy (logistic regression on the 16 × 16 mask; majority class, 36.0%). The heads therefore relied little on the appearance of the lesion: the tumor-versus-no-tumor decision rested mainly on cues outside the lesion, consistent with the source separation of the no-tumor class (Section 3.1), and the tumor type was partly predictable from the location, size and shape of the lesion and from the surrounding anatomy.

Within a single source (the three-class tumor task on figshare-derived images), the VQC head reached 80.8% against 86.4% for its control, 86.2% for the deeper control, 84.5% for the Fourier surrogate and 87.5% for the linear probe (VQC minus control, −5.7 pp; 95% CI −6.9 to −4.5; better in 0 of 20 blocks). Across sources, every head failed: trained on figshare-derived tumors and tested on the SARTAJ tumor images, the heads reached 36.1–38.8% accuracy (chance, 33.3%; balanced accuracy 46.4–53.5%), recognizing most pituitary tumors but few gliomas and meningiomas, and the VQC head did not differ from its control (+2.5 pp; 95% CI −1.4 to +6.4; balanced accuracy 46.4% versus 49.8%). The features that separate tumor types within one source therefore transfer poorly to another source, for quantum and classical heads alike.

### 3.7. End-to-End Fine-Tuning and Training from Scratch

**Figure 8:**
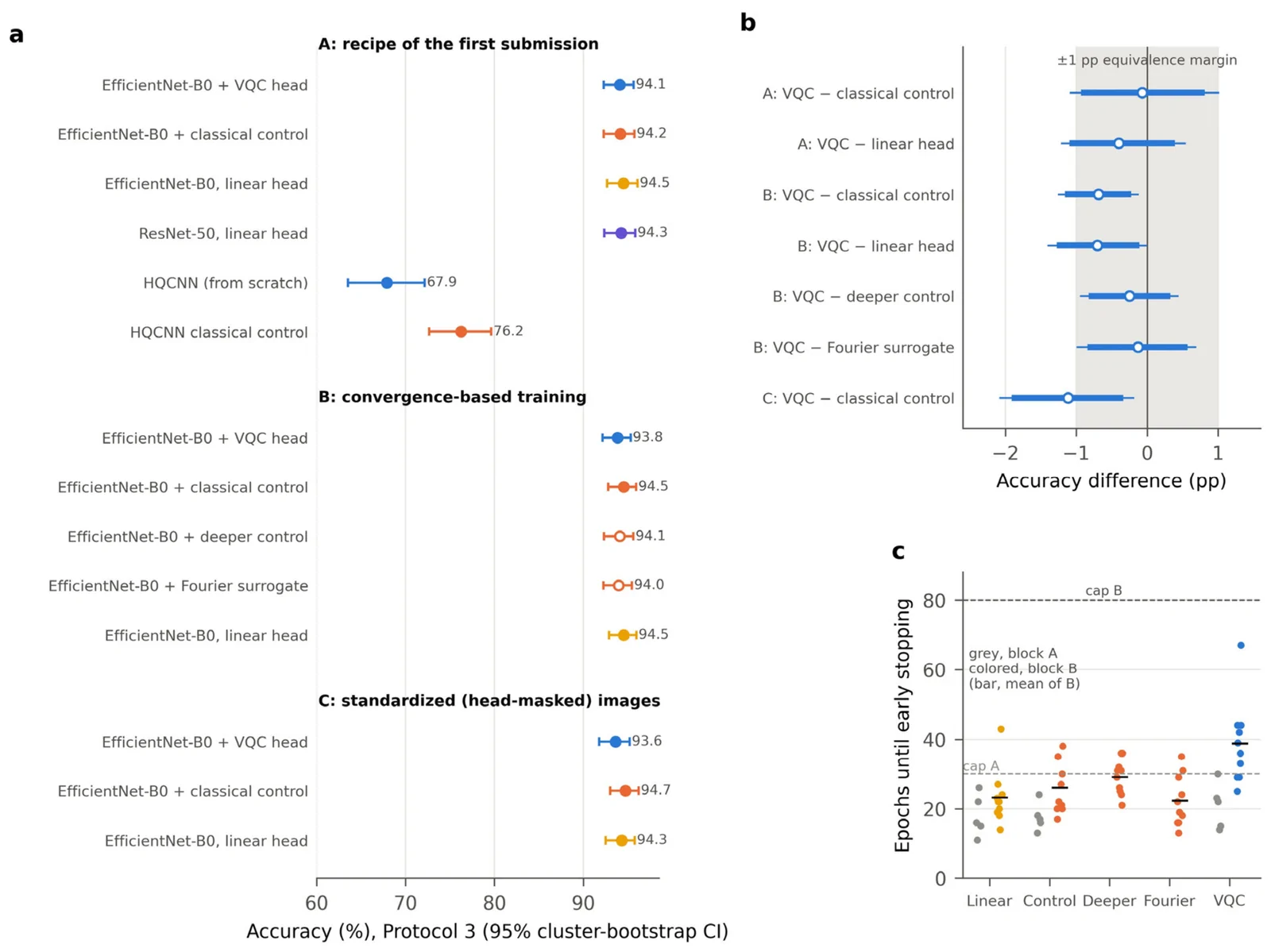
End-to-end models under Protocol 3. (**a**) Accuracy with 95% cluster-bootstrap CIs in blocks A (recipe of the first submission), B (convergence-based training, two seeds) and C (standardized images). (**b**) Accuracy of the EfficientNet-B0 model with the VQC head minus that of each comparator (dot, estimate; thin line, 95% CI; thick line, 90% CI); the shaded band is the ±1 pp equivalence margin. The HQCNN minus its control (−8.3 pp) lies outside the axis. (**c**) Epoch at which each training run stopped. Each dot represents an individual run. Gray dots indicate Block A (maximum 30 epochs). For Block B (maximum 80 epochs), yellow dots represent the linear head, orange dots represent the classical-control variants (standard, deeper, and Fourier surrogate), and blue dots represent the VQC head. Horizontal bars indicate the mean stopping epoch for Block B.

Under Protocol 3, fine-tuning raised the pretrained models to 93.6–94.7% accuracy (Table 4, Figure 8a), about 3.5 pp below the Protocol 2 accuracies of the first submission (Section 3.8). With the recipe of the first submission (block A), EfficientNet-B0 reached 94.1% (95% CI 92.3 to 95.6) with the VQC head, 94.2% with the classical control and 94.5% with the linear head, and ResNet-50 reached 94.3%. The VQC head and its control did not differ (−0.07 pp; 95% CI −1.08 to 1.00; McNemar *p* = 0.83, with 177 images correct only with the VQC head and 182 only with the control), and the 90% CI (−0.93 to 0.82) lay within the ±1 pp margin (Figure 8b).

With convergence-based training (block B), the learning-rate search selected 10^−3^ for all five heads, and no run reached the limit of 80 epochs. The VQC head trained longest: its runs stopped after 38.8 epochs on average (best epoch 28.8), against 22.3–29.1 epochs for the classical heads (Figure 8c, Table 5); with the 30-epoch limit of block A, one of its five runs reached the limit, against six of eight in the first submission. Trained to convergence, the VQC head was 0.69 pp less accurate than its classical control (95% CI −1.25 to −0.13; 93.8% versus 94.5%); the 90% CI (−1.16 to −0.22) crossed the −1 pp margin, so equivalence could not be declared (smallest equivalence margin 1.16 pp). The run-level analysis agreed in direction: the VQC head was less accurate in eight of 10 fold–seed pairs (mixed model, −0.69 pp; 95% CI −1.30 to −0.07), although the variance-corrected resampled *t*-test was not significant (*p* = 0.27), and the McNemar test on the two-seed ensembles found no difference (*p* = 0.39). Against the deeper control (−0.25 pp; 90% CI −0.83 to 0.33) and the Fourier surrogate (−0.13 pp; 90% CI −0.84 to 0.57), the VQC head was equivalent within ±1 pp, and the classical control and the linear head were equivalent within 0.50 pp (−0.01 pp). The VQC head was also 0.70 pp less accurate than the linear head (95% CI −1.39 to −0.01); after Holm correction, none of the secondary McNemar tests in block B was significant (all adjusted *p* ≥ 0.13).

The VQC head came at a cost (Table 5). In block B, a run took 12.8 min on average, against 6.8 min with the classical control and 6.0 min with the linear head, and the single-image latency was 18.8 ms against 11.3 and 10.7 ms, because the circuit is simulated; peak GPU memory was the same for all EfficientNet-B0 heads (1461 MB). Calibration did not differ consistently: the mean expected calibration error was 0.037 with the VQC head and 0.040 with the classical control in block B, but 0.059 versus 0.048 in block A. Averaging the predictions of the two seeds raised the accuracy of every head in block B, raising that of the VQC head the most (to 95.0%, against 95.2% for the control, 95.1% for the linear head, 94.5% for the deeper control and 94.8% for the Fourier surrogate); the ensemble of the VQC head was correct on more of the images on which it disagreed with the deeper control (144 versus 108; McNemar *p* = 0.03, Holm-adjusted 0.14). Fold- and seed-level accuracies and validation curves are given in Table S6 and Figure S3. Class-specific sensitivity, specificity, F1 score and AUC with 95% CIs are given in Table S10 and the confusion matrices in Figure S4; for every head in block B, the sensitivity of the two-seed ensemble was lowest for meningioma (90.2–93.0%) and highest for no tumor (97.8–98.6%).

Trained from scratch, the HQCNN reached 67.9% (95% CI 63.5 to 72.1) and its classical control 76.2% (95% CI 72.7 to 79.6), a difference of −8.33 pp (95% CI −12.72 to −4.06); McNemar *p* = 1.6 × 10^−41^. The HQCNN assigned most meningioma images to glioma (meningioma sensitivity 13.8%, against 52.2% for its control), and both models varied similarly between folds (standard deviation 6.3 and 6.2 pp). Both from-scratch models remained 17–27 pp less accurate than every fine-tuned model.

### 3.8. How Much Leakage Inflated Accuracy

**Figure 9:**
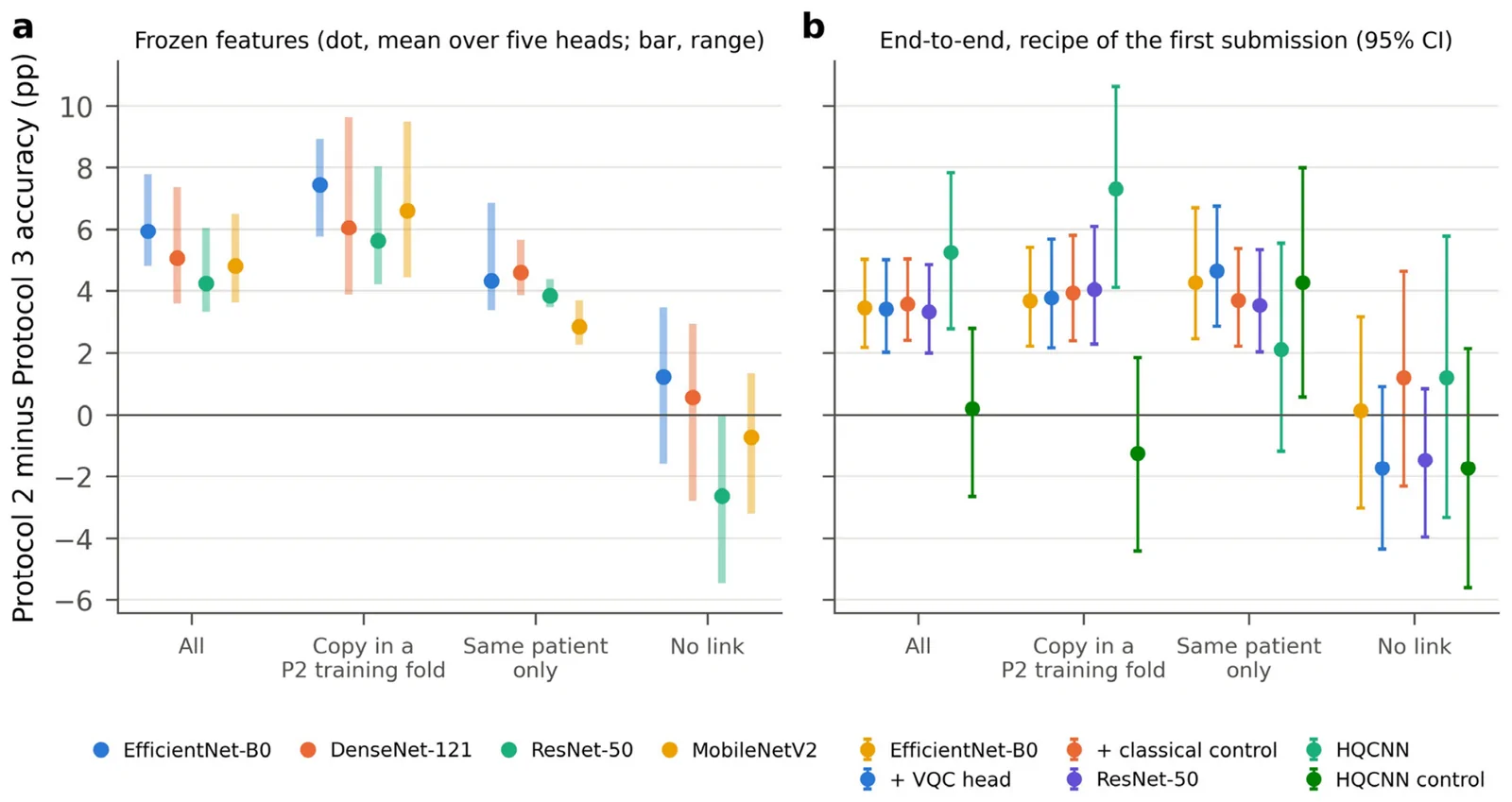
Effect of leakage on accuracy: the same models trained on the same images under Protocol 2 (duplicate groups of the original audit) and Protocol 3 (patients and mirror- and rotation-invariant duplicate groups), by the link that an image had to a Protocol 2 training fold. (**a**) Frozen-feature heads (dot, mean over the five heads; bar, range). (**b**) End-to-end models trained with the recipe of the first submission (95% cluster-bootstrap CI).

Training the same models on the same images under Protocol 2 and Protocol 3 isolates the effect of leakage on accuracy (Figure 9). With frozen features, every head lost 3.3–7.8 pp under Protocol 3, the VQC head the most (6.0–7.8 pp) and the linear probe the least (3.3–4.8 pp). The loss was 3.9–9.6 pp for images with a detected copy in a Protocol 2 training fold and 2.3–6.9 pp for images that shared only a patient with it, but it was small and of inconsistent sign for the 750 unlinked images (−5.5 to 3.5 pp; mean −0.4 pp). The fine-tuned models trained with the recipe of the first submission behaved alike: they lost 3.3–3.6 pp overall, 3.7–4.0 pp on images with a copy and 3.5–4.6 pp on images that shared only a patient, but −1.7 to 1.2 pp on unlinked images, with every 95% CI of the last group including zero. The HQCNN lost 5.2 pp (95% CI 2.8 to 7.8), whereas the accuracy of its classical control did not change overall (0.2 pp; −2.7 to 2.8). Leakage therefore inflated the accuracy of the first submission by 3–8 pp and slightly favored the VQC head, whose deficit relative to its control widened from 4.4 to 6.0 pp with frozen features. The estimate of less than 0.5 pp in the first submission was wrong: it compared leaked with clean test images of the official partition, which differ in source as well as in leakage, and it relied on the incomplete duplicate audit.

Accuracy also depended strongly on the source of an image (Table 4). Under Protocol 3, the fine-tuned models classified 93.6–94.6% of the figshare-derived tumor images and 96.8–98.8% of the no-tumor images correctly, but only 77.0–81.4% of the tumor images from other sources and 46.1–61.8% of the 76 out-of-source glioma images, which were misread mostly as meningioma and otherwise as no tumor; the from-scratch models recognized 2.6–6.6% of them. On the official partition, a re-analysis of the submitted runs with the revised audit showed the same pattern (Table S11): fine-tuned models classified 98.1–99.6% of the test images with a copy in training and 97.0–98.6% of those that shared only a patient correctly, but only 29.5–37.2% of the 87 strict-clean test images, 74 of which are out-of-source glioma images.

### 3.9. Grad-CAM

**Figure 10:**
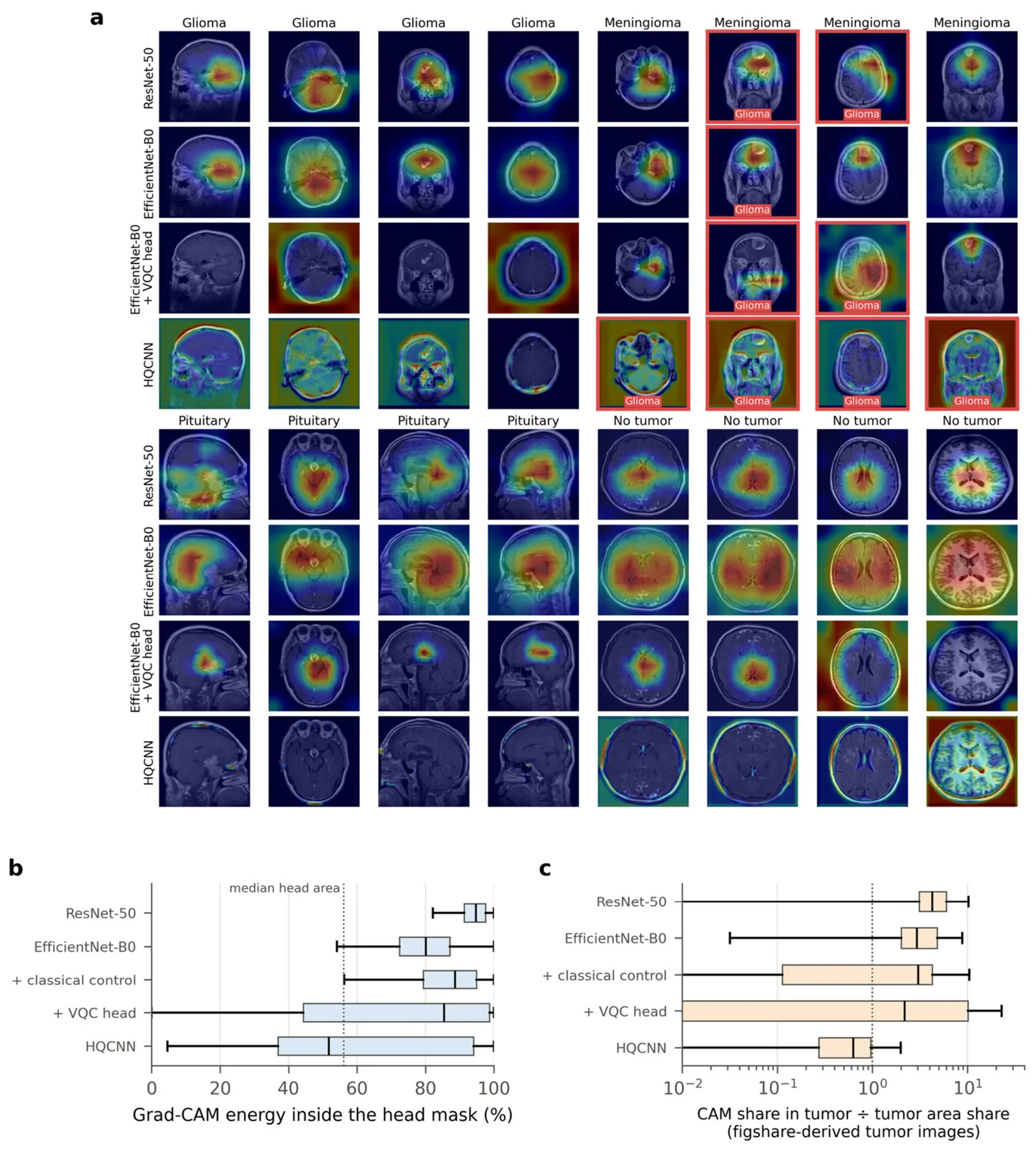
Grad-CAM for the models of block A (Protocol 3, first fold). (**a**) Maps for the predicted class on 16 test images (four per class, selected at random before any prediction was inspected); a red frame and label mark a wrong prediction. (**b**) Share of the Grad-CAM energy inside the head mask for all 1400 test images of the fold (boxes, quartiles; whiskers, 5th–95th percentiles; dotted line, median head area). (**c**) Share of the energy inside the manual tumor mask divided by the share of the image covered by the tumor, for the figshare-derived tumor images (dotted line, no enrichment).

Over all 1400 test images of the first fold (block A), most of the Grad-CAM energy of the fine-tuned models fell inside the head mask (Figure 10b): the median share was 95% for ResNet-50, 80% for EfficientNet-B0 with the linear head, 89% with the classical control and 85% with the VQC head, against a median head area of 56% of the image. With the VQC head, however, less than half of the energy fell inside the head in 28% of the images with a non-zero map (classical control 10%, linear head 0.2%, ResNet-50 none), and for 137 images (classical control, 87), the map was zero because the bounded bottleneck had saturated. The HQCNN placed a median of only 52% of its energy inside the head and less than half in 48% of the images. For the figshare-derived tumor images, the share of the energy inside the manual tumor mask exceeded the share of the image covered by the tumor by a median factor of 4.3 for ResNet-50, 2.9 for EfficientNet-B0, 3.0 with the classical control and 2.2 with the VQC head; for the HQCNN, it was below the tumor area (factor 0.63; Figure 10c). In the 16 examples (Figure 10a), the pretrained models with linear heads highlighted central brain structures and many lesions, the VQC head highlighted the background or the skull outline in two of the four glioma examples and one no-tumor example, and the HQCNN highlighted the skull and the background and labeled all four meningioma examples as glioma.

## 4. Discussion

### 4.1. Does the Quantum Layer Help?

In none of the three regimes did the six-qubit VQC improve on a classical layer of the same width (Table 6), and this held under patient-level separation, with tuned and convergence-based training, on standardized images and within a single source; across sources, every head failed alike. With frozen features, it was clearly worse; with end-to-end fine-tuning, it was within about 1 pp of its classical control (equivalent within ±1 pp with the recipe of the first submission, 0.7 pp less accurate after convergence-based training and 1.1 pp less accurate on standardized images) but slower to train and to run; and inside a small network trained from scratch, it was 8.3 pp less accurate than its control. The ablations point in the same direction. The most accurate circuit was the one without entanglement, a product of single-qubit rotations whose outputs can be computed classically qubit by qubit; more layers narrowed the gap to the classical control but did not close it, and data re-uploading widened it; and the quantum kernel was slightly less accurate than the RBF kernel at every width. Most tellingly, a classical Fourier surrogate that can represent every function the circuit can compute was more accurate than the circuit itself. These results agree with benchmarks in which removing entanglement rarely hurts and simple classical models usually win [30], and they suggest that the parameter efficiency attributed to hybrid models in earlier brain tumor studies [19,20,21,22] comes from their small classical encoders rather than from the circuit: the HQCNN and its control have the same size, and the control was on average more accurate.

### 4.2. Why the Circuit Does Not Help

Several properties of the dressed-circuit design limit what the circuit can add. When every feature is encoded once as a rotation angle, each output ⟨*Z_i_*⟩ is a trigonometric polynomial of degree one in each input angle [49], so a two-layer circuit on six qubits represents a restricted family of smooth functions of six numbers. The angles themselves are produced by a trainable linear map from the backbone features, and a trainable linear map reads out the result, so the classical layers around the circuit can absorb much of what it computes. This is consistent with the end-to-end results: once a four-million-parameter backbone could adapt to the circuit, the deficit seen with frozen features shrank from about 6 pp to about 1 pp or less. The Fourier surrogate makes the point sharper. Its 729 features span every function a circuit of this design can compute, so any accuracy the circuit can reach is also reachable by a linear classifier on classically computed features; the surrogate was in fact more accurate, consistent with the circuit reaching only part of that function class through its 36 parameters and a non-convex training problem. The circuit also appeared harder to optimize than its classical counterpart: it needed more epochs, it lost most accuracy when data were scarce, and wider circuits of eight and ten qubits became less accurate, whereas their classical controls changed by less than 1 pp, which is consistent with, although not proof of, the flattening of loss landscapes known as barren plateaus [86,87,88]. Finally, a learning advantage for quantum models on classical data requires data whose structure is hard for classical models [89], and nothing in brain MRI classification suggests such structure; at bandwidths of 0.1 and above, the quantum kernel concentrated as the number of qubits increased [64]; the smallest bandwidth, which validation selected in most folds, avoided this, although it made the kernel nearly constant with few qubits, and the quantum kernel nonetheless remained slightly less accurate than the RBF kernel.

### 4.3. Implications for Benchmarking Quantum Models in Medical Imaging

Our results suggest minimum standards for studies of hybrid quantum models on medical images. First, every hybrid model should be compared with the same architecture in which the quantum layer is replaced by a classical layer of equal width, trained under the same protocol and ideally with a classical surrogate of the circuit’s function class; comparisons with unrelated CNNs, or with CNNs trained under other protocols, cannot attribute accuracy to the circuit. Second, the test set must not contain duplicates of training images or images of the same patients; when patient identifiers are missing, they should be recovered from the source collections where possible, and duplicate detection must be invariant to reflections and rotations, because public collections contain mirrored and rotated copies. Third, small differences should be tested at the image level with methods that respect the correlation between near-duplicate images, and equivalence rather than the absence of a significant difference should be tested when the claim is that a smaller model is as good as a larger one. Fourth, claims of readiness for quantum hardware should report accuracy under noise and finite shot budgets. Reporting checklists for artificial intelligence in medical imaging, such as the Checklist for Artificial Intelligence in Medical Imaging (CLAIM) [90], cover the data requirements but not the matched control.

Recent hybrid studies of brain MRI illustrate why these standards matter (Table 7). Seven studies published in 2025–2026 report 94–98.6% accuracy for hybrid models on four-class, three-class or binary tasks [23,24,25,26,27,28,29]. Only one of them used the dataset studied here [23], one compared the hybrid model with an explicit parameter-matched classical counterpart (in a single run) [23], one split the data by patient [25], and none reported a check for duplicates; two classical studies of 2026 addressed leakage in this dataset explicitly [41,42]. Because none of these studies used patient-level splits of this dataset, their accuracies cannot be compared with ours, and the gains they attribute to quantum components cannot be separated from those of their classical parts.

### 4.4. Implications for the Dataset

Contrary to its description, version 2 of the Kaggle Brain Tumor MRI Dataset is not free of leakage, and the leakage is far larger than the first submission reported: 79% of its test images have a copy in the training partition, most of them a mirrored or rotated one, and all but two of the 917 figshare-derived test images share a patient with it. The cause is that 1755 figshare slices were imported two to five times, typically once as a mirrored grayscale and once as an unmirrored RGB image, and the no-tumor class contains many processed copies of the same slices. This agrees with independent reports of near-duplicates in both releases [39,40,41,42]. Leakage of this size matters for evaluation: under the patient-grouped protocol, frozen-feature accuracy fell by 3.3–7.8 pp and fine-tuned accuracy by 3.3–3.6 pp, and the effect was concentrated on exactly the images that had a copy or a patient in the training folds. For fine-tuned models, the out-of-source glioma subgroup matters as well. Because the training glioma images come from a single source and the no-tumor images almost entirely from another, a model can separate these classes partly by source, and test gliomas from a different source are then read as meningioma or as no tumor. We cannot exclude that these images also differ in tumor characteristics, such as grade, sequence or plane, and the two explanations are not mutually exclusive. Some of these images may also be mislabeled: all 76 are SARTAJ test images of the glioma class, the set that the curator describes as replaced because of labeling problems [9], so the replacement left these test images in place; three of the 76 were flagged by confident learning, and for figshare-derived images, the original annotation contradicts the version 2 label in 13 cases. The cross-source task makes the same point for frozen features: heads trained on figshare-derived tumors classified the SARTAJ tumors barely above chance. The lesion-occlusion test points the same way: with the tumor removed, the heads still classified most figshare-derived tumors correctly and almost never as no tumor, so on this dataset, the tumor-versus-no-tumor decision depends little on the lesion’s appearance. We recommend that studies using this dataset use patient-grouped splits such as our Protocol 3, report accuracy separately for in-source and out-of-source test images and avoid the official partition for model comparison, and that the out-of-source images and the flagged candidates be reviewed by a neuroradiologist. Our splits and per-image annotations, including the figshare matches and patient groups, are released for this purpose.

### 4.5. Limitations

This study has limitations. First, it uses a single public dataset of two-dimensional slices. Patient identity could be recovered only for the 4846 figshare-derived tumor images; for the remaining tumor images and the no-tumor images, grouping relies on duplicate detection, which may leave other slices of the same examination in different folds, and our conclusions about the quantum layer may not transfer to other tasks or to three-dimensional data. Second, all circuits were simulated, and the noise model (uniform single-qubit depolarizing noise and shot noise) omits crosstalk, readout error, limited connectivity and the cost of loading data on real hardware; the reported training times and latencies are those of the simulation on a GPU and do not predict execution times on a quantum processor. Third, we studied dressed circuits with up to ten qubits and a single encoding scheme; quantum convolutional networks acting on quantum data [15], other encodings and larger circuits were not tested, although larger circuits raise the trainability concerns discussed above. Fourth, the end-to-end comparison used two seeds per fold and a learning-rate search over three values on one fold; a broader hyperparameter search could change small differences. Fifth, head masking removed the background but not the skull, because robust skull stripping of two-dimensional slices in three planes is not available, and coarse anatomy and imaging plane remain informative; the shortcut controls therefore bound, rather than eliminate, the contribution of non-morphological cues. Sixth, Grad-CAM summarizes where the models attend but not why. Seventh, candidate label errors and out-of-source images were not reviewed by a radiologist, so we cannot say how many of the errors on out-of-source glioma images are model errors rather than label errors.

*Clinical scope.* The accuracies reported here measure relative performance under a common protocol on a curated public benchmark; they are not estimates of diagnostic accuracy. A clinical claim would require examination-level rather than slice-level inputs, patient-level separation for all classes, a reference standard such as histopathology or expert consensus, a no-tumor class drawn from the same clinical population as the tumor classes and validation on independent, multi-institutional cohorts. Our conclusions concern whether a quantum layer contributes and how such benchmarks should be built, not the clinical use of any model.

## 5. Conclusions

On a widely used brain tumor MRI benchmark audited for leakage, a dressed variational quantum circuit (angle encoding, hardware-efficient ansatz with a CNOT ring, 2–10 qubits, 1–6 layers, simulated without noise and with depolarizing noise) did not improve on classical layers of the same width in any regime we tested (apart from a small, inconsistent gain with two qubits, where the classical control was at its least accurate): it was worse on frozen ImageNet features, including against a classical surrogate of its own function class; equivalent within ±1 pp with end-to-end fine-tuning of EfficientNet-B0 under the recipe of the first submission and 0.7 pp worse, without equivalence, under tuned, convergence-based training; and 8.3 pp worse in a small network trained from scratch. A quantum kernel was slightly less accurate than a classical one (by 0.3–1.0 pp). These conclusions held under patient-level separation, which we reconstructed from the original figshare collection, on standardized images and within a single source, whereas across sources, every head, quantum or classical, failed alike. The dataset’s second release contains mirrored, rotated and re-encoded copies of many figshare slices, so that 79% of its test images have a copy in training, and 915 of the 917 figshare-derived test images share a patient with it; evaluating on it without patient-level grouping overstates accuracy by several percentage points. Claims of quantum benefit in medical image classification should be tested against matched classical controls on patient-grouped, mirror-invariant leakage-free splits, with test errors broken down by source.

## Figures and Tables

**Table 1:** Composition and audit of the Brain Tumor MRI Dataset, version 2.

Class	Training Images	Test Images	Not 512 × 512, Training (%)	Not 512 × 512, Test (%)	Figshare-Derived Test Images	Test Linked by a Copy, Original Audit ^a^	Test Linked by a Copy, Revised Audit ^b^	Test Linked by Patient Only ^c^	Clean Test, Original	Strict-Clean Test ^d^
Glioma	1400	400	0 (0)	76 (19)	324	48	143	183	352	74
Meningioma	1400	400	133 (10)	117 (29)	193	128 + 92 ^e^	276 + 101 ^e^	10	169	11
Pituitary tumor	1400	400	72 (5)	8 (2)	400	115	349	49	285	2
No tumor	1400	400	1383 (99)	397 (99)	0	374	400	0	26	0
**Total**	**5600**	**1600**	**1588 (28)**	**598 (37)**	**917**	**665 + 92 ^e^**	**1168 + 101 ^e^**	**242**	**832**	**87**

^a^ Non-augmented test images in a duplicate group (exact or near duplicates, no reflections or rotations) that also contain a training image; 114 of the 665 are pixel-identical to a training image. ^b^ Mirror- and rotation-invariant detection (Section 2.2). ^c^ Figshare-derived test images without a detected copy in training but with another image of the same patient in training. ^d^ Test images linked to training neither by a copy (revised audit) nor by a patient. ^e^ Augmented test images linked to a training image. The clean subset of the first submission excludes all test images linked by the original audit and all augmented test images.

**Table 2:** Accuracy (%) of heads trained on frozen ImageNet features (Protocol 3, patient-grouped five-fold cross-validation; mean ± SD over folds of the seed-averaged accuracy).

Head	EfficientNet-B0	DenseNet-121	ResNet-50	MobileNetV2	Parameters ^a^	Mean Rank ^b^
VQC head (6 qubits, 2 layers)	82.0 ± 3.4	79.5 ± 3.5	76.0 ± 4.0	78.5 ± 2.1	7750 (36 q)	7.20
Classical control (Linear 6→6 + tanh)	86.4 ± 2.9	86.4 ± 3.1	83.1 ± 3.1	84.3 ± 3.9	7756	4.45
Deeper control (2 × [Linear 6→6 + tanh])	84.4 ± 2.6	85.5 ± 2.8	81.3 ± 2.9	83.7 ± 3.7	7798	5.55
Fourier surrogate (729 features)	85.5 ± 3.3	85.4 ± 3.6	82.1 ± 2.6	84.2 ± 2.9	12,094	5.00
Bottleneck only	87.6 ± 3.2	87.9 ± 3.6	85.5 ± 2.5	86.4 ± 3.6	7714	2.78
Linear probe	88.1 ± 3.0	88.1 ± 3.1	86.0 ± 2.3	87.1 ± 3.6	5124	1.92
MLP (256 hidden units)	87.9 ± 2.8	88.8 ± 3.2	86.3 ± 2.1	87.5 ± 3.7	328,964	1.35
Untrained VQC	80.6 ± 4.5	78.1 ± 4.8	69.7 ± 3.0	77.0 ± 5.8	7714 ^c^	7.75

^a^ Trainable parameters on EfficientNet-B0 features (*d* = 1280); q, quantum parameters. ^b^ Friedman mean rank over the 20 backbone–fold blocks (1 = best). ^c^ The 36 circuit parameters are fixed at random values. Three seeds per fold, except the untrained VQC (one seed). MLP, multilayer perceptron; SD, standard deviation; VQC, variational quantum circuit.

**Table 3:** Shortcut and source controls (Protocol 3).

Control	VQC Head	Classical Control	Linear Probe	VQC − Control, pp (95% CI)	VQC Better
Frozen features, original images (four backbones) ^a^	79.5	85.8	87.5	—	—
Frozen features, standardized images (four backbones) ^a^	78.6	85.3	86.9	−6.7 (−8.7 to −4.8)	1/20 blocks
Fine-tuned EfficientNet-B0, standardized images (block C)	93.6	94.7	94.3	−1.1 (−2.1 to −0.2)	—
Lesion occlusion: figshare tumors, unoccluded ^b^	80.6	85.6	87.3	—	—
Lesion occlusion: figshare tumors, occluded ^b^	78.9	83.0	84.7	—	—
Lesion occlusion: occluded tumors called no tumor (%) ^b^	2.2	0.5	0.3	—	—
Single source: three tumor classes, figshare only ^c^	80.8	86.4	87.5	−5.7 (−6.9 to −4.5)	0/20 blocks
Cross-source: figshare → SARTAJ tumors (chance 33.3%) ^d^	38.6	36.1	37.0	+2.5 (−1.4 to +6.4)	2/4 backbones

Accuracy (%), mean over backbones (frozen features) or pooled out-of-fold (block C). ^a^ Seed 0, five folds, 20 backbone–fold blocks. ^b^ Heads trained on unoccluded images (seed 0) and evaluated on the figshare-derived tumor images of their test folds, with the tumor (manual mask, dilated by 8 pixels) replaced by the median head intensity; four backbones; the tumor mask alone predicted the tumor class with 61.4% accuracy. ^c^ Patient-grouped five-fold cross-validation, mean over three seeds (block = backbone–fold). ^d^ Trained on all figshare-derived tumor images (patient-grouped validation) and tested on the 382 tumor images from SARTAJ that are not linked to any figshare image; mean over three seeds; CI over the four backbones; balanced accuracy, VQC head 46.4%, classical control 49.8%, linear probe 51.7%. All heads and backbones are given in Table S13.

**Table 4:** Accuracy of fine-tuned and from-scratch models under Protocol 3 (patient-grouped five-fold cross-validation).

Model	Runs	Accuracy, % (95% CI)	Macro-F1, %	Macro-AUC, %	ECE	Figshare-Derived Tumor, % ^a^	Other-Source Tumor, % ^a^	Out-of-Source Glioma, % ^a^	No Tumor, % ^a^
*Block A: recipe of the first submission*									
EfficientNet-B0 + VQC head	5	94.1 (92.3–95.6)	94.0	98.5	0.059	93.7	79.8	46.1	98.1
EfficientNet-B0 + classical control	5	94.2 (92.3–95.8)	94.1	98.1	0.048	93.8	77.2	50.0	98.8
EfficientNet-B0, linear head	5	94.5 (92.7–96.1)	94.4	98.9	0.037	94.4	78.5	55.3	98.1
ResNet-50, linear head	5	94.3 (92.4–95.8)	94.2	98.9	0.027	94.2	78.8	61.8	97.8
HQCNN (from scratch)	5	67.9 (63.5–72.1)	63.6	89.8	0.099	63.2	21.2	6.6	90.4
HQCNN classical control	5	76.2 (72.7–79.6)	75.2	92.0	0.092	74.4	32.7	2.6	90.6
*Block B: convergence-based training*									
EfficientNet-B0 + VQC head	10	93.8 (92.2–95.3)	93.7	98.3	0.037	94.1	77.2	54.6	96.8
EfficientNet-B0 + classical control	10	94.5 (92.8–95.9)	94.4	98.4	0.040	94.5	78.9	50.7	97.8
EfficientNet-B0 + deeper control	10	94.1 (92.3–95.6)	94.0	98.0	0.036	94.1	77.1	55.9	97.8
EfficientNet-B0 + Fourier surrogate	10	94.0 (92.3–95.5)	93.9	98.1	0.034	93.6	78.4	55.9	98.2
EfficientNet-B0, linear head	10	94.5 (92.9–96.0)	94.4	98.8	0.029	94.6	77.6	53.9	98.0
*Block C: standardized images*									
EfficientNet-B0 + VQC head	5	93.6 (91.8–95.2)	93.5	98.1	0.051	93.6	77.0	56.6	97.1
EfficientNet-B0 + classical control	5	94.7 (93.0–96.2)	94.6	98.5	0.044	94.5	81.4	57.9	98.3
EfficientNet-B0, linear head	5	94.3 (92.5–95.8)	94.2	98.7	0.029	94.4	77.0	46.1	97.9

Out-of-fold predictions for the 6997 non-augmented images, averaged over the runs of each model (block B: five folds × two seeds; blocks A and C: five folds); 95% CI from a cluster bootstrap over patient and duplicate groups; macro-F1 and macro-AUC of the pooled out-of-fold predictions of each seed, averaged over seeds; ECE, mean expected calibration error over runs. ^a^ Accuracy (recall) for the figshare-derived tumor images (4815), the tumor images from other sources (382, including the 76 out-of-source glioma images), the 76 out-of-source glioma images of the official test partition and the no-tumor images (1800). AUC, area under the receiver operating characteristic curve; ECE, expected calibration error; HQCNN, hybrid quantum–classical CNN; VQC, variational quantum circuit. Italicized block headings indicate the different experimental training regimes evaluated in this study.

**Table 5:** Training and inference cost of the end-to-end models (Protocol 3).

Model	Parameters	Epochs (Mean)	Best Epoch (Mean)	Runs at the Epoch Limit	Training Time per Run (min)	Latency (ms per Image)	Peak GPU Memory (MB)
*Block A: recipe of the first submission*							
EfficientNet-B0 + VQC head	4,015,298	20.8	14.8	1/5	6.9	18.8	1467
EfficientNet-B0 + classical control	4,015,304	17.6	11.6	0/5	4.6	10.9	1467
EfficientNet-B0, linear head	4,012,672	18.0	12.0	0/5	4.8	10.8	1466
ResNet-50, linear head	23,516,228	17.2	11.2	0/5	3.7	8.9	1795
HQCNN (from scratch)	375,950	18.6	13.4	1/5	4.5	9.3	1272
HQCNN classical control	375,956	19.0	13.0	0/5	1.8	1.4	1216
*Block B: convergence-based training*							
EfficientNet-B0 + VQC head	4,015,298	38.8	28.8	0/10	12.8	18.8	1461
EfficientNet-B0 + classical control	4,015,304	26.0	16.0	0/10	6.8	11.3	1461
EfficientNet-B0 + deeper control	4,015,346	29.1	19.1	0/10	7.7	11.2	1461
EfficientNet-B0 + Fourier surrogate	4,019,642	22.3	12.3	0/10	6.0	11.7	1461
EfficientNet-B0, linear head	4,012,672	23.2	13.2	0/10	6.0	10.7	1461
*Block C: standardized images*							
EfficientNet-B0 + VQC head	4,015,298	24.2	18.2	1/5	7.8	18.8	1461
EfficientNet-B0 + classical control	4,015,304	21.0	15.0	0/5	5.5	11.0	1461
EfficientNet-B0, linear head	4,012,672	14.8	8.8	0/5	3.9	10.7	1461

Means over the runs of each model. Training stopped after six (blocks A and C, at most 30 epochs) or ten (block B, at most 80 epochs) epochs without improvement of the validation loss. Training time includes data loading and augmentation; latency is the per-run median of 50 single-image forward passes (batch size 1) on the GPU after 20 warm-up passes, including the simulation of the circuit; all runs used one NVIDIA GeForce RTX 3090 (Section 2.10). Italicized block headings indicate the different experimental training regimes evaluated in this study.

**Table 6:** Summary of quantum-versus-classical comparisons (Protocol 3).

Regime	Comparison	Unit of Analysis	Accuracy Difference, pp (95% CI)	Test, *p*	Interpretation
Frozen features, four backbones	VQC head − classical control	20 backbone–fold blocks (3 seeds)	−6.03 (−6.78 to −5.20)	Wilcoxon, 1.9 × 10^−6^	Classical better in 20/20 blocks
Frozen features, four backbones	VQC head − deeper control	20 blocks	−4.71 (−5.63 to −3.73)	Wilcoxon, 1.9 × 10^−6^	Classical better in 20/20 blocks
Frozen features, four backbones	VQC head − Fourier surrogate	20 blocks	−5.27 (−6.20 to −4.39)	Wilcoxon, 1.9 × 10^−6^	Surrogate of the circuit’s function class better in 20/20 blocks
Frozen features, four backbones	VQC head − untrained VQC	20 blocks	+2.67 (1.11 to 4.24)	Wilcoxon, 0.0083	Training the circuit helps (16/20 blocks)
Frozen features, EfficientNet-B0	VQC without entanglement − VQC	5 folds	+2.89 (range −0.43 to +5.50)	—	Better in 4/5 folds
Frozen features, EfficientNet-B0	VQC without entanglement − classical control	5 folds	−0.27 (range −2.07 to +1.79)	—	Better in 2/5 folds
Frozen features, EfficientNet-B0	VQC with re-uploading − VQC	5 folds	−5.33 (range −10.07 to −2.57)	—	Worse in 5/5 folds
Kernel SVM, EfficientNet-B0 PCA	ZZ fidelity kernel − RBF kernel, 4/6/8/10 qubits	5 folds	−0.53/−0.33/−1.04/−0.56 (fold means)	—	ZZ better in 1/2/0/3 of 5 folds
End-to-end, recipe of the first submission	EfficientNet-B0: VQC head − classical control	6997 images	−0.07 (−1.08 to 1.00)	McNemar, 0.83	Equivalent within ±1 pp
End-to-end, convergence-based training	EfficientNet-B0: VQC head − classical control	6997 images (2 seeds)	−0.69 (−1.25 to −0.13)	McNemar, 0.39	Not equivalent within ±1 pp (smallest margin 1.16 pp)
End-to-end, standardized images	EfficientNet-B0: VQC head − classical control	6997 images	−1.11 (−2.07 to −0.20)	McNemar, 4.0 × 10^−5^	Not equivalent within ±1 pp (smallest margin 1.91 pp)
End-to-end, convergence-based training	EfficientNet-B0: VQC head − deeper control	6997 images (2 seeds)	−0.25 (−0.94 to 0.43)	McNemar, 0.03 ^a^	Equivalent within ±1 pp
End-to-end, convergence-based training	EfficientNet-B0: VQC head − Fourier surrogate	6997 images (2 seeds)	−0.13 (−0.98 to 0.68)	McNemar, 0.39 ^a^	Equivalent within ±1 pp
From scratch	HQCNN − classical control	6997 images	−8.33 (−12.72 to −4.06)	McNemar, 1.6 × 10^−41^	Classical better

Frozen-feature and kernel comparisons use Protocol 3 with fold means (three seeds averaged for the main heads; one seed for ablations and kernels); end-to-end comparisons use pooled out-of-fold predictions with 95% cluster-bootstrap CIs over patient and duplicate groups; block B averages two seeds per image, and its McNemar tests compare the two-seed ensembles. ^a^ Secondary contrast, unadjusted; all Holm-adjusted *p* ≥ 0.13. PCA, principal component analysis; RBF, radial basis function; SVM, support vector machine; VQC, variational quantum circuit.

**Table 7:** Hybrid quantum–classical studies of brain MRI classification published in 2025–2026, compared with this study.

Study	Data and Task	Quantum Component (Simulated)	Reported Result	Matched Classical Control	Duplicates/Patient Split
Sheu et al. [23]	Kaggle (this dataset), four classes	2-qubit circuit recalibrating class logits	F1 96.2%	Yes (single run)	Not reported
Valarmathi et al. [24]	Kaggle (other dataset), binary	4-qubit VQC after ResNet-50	Accuracy 94.7%	No	Not reported
Harshavardhan et al. [25]	BraTS 2021, high- vs. low-grade glioma	Amplitude-encoded PQC	Accuracy 94.1%	Partial (unmatched size)	Patient-level CV
Rafikov et al. [26]	Br35H, binary	2-qubit VQC at the output	Accuracy up to 98.6%	CNN baseline	Not reported
Ait Haddou and Bennai [27]	figshare + two other sources, four classes	5 × 5-qubit amplitude-encoded circuits	Accuracy 96.5%	Not described	Not reported
Soliman et al. [28]	BraTS 2021	8-qubit VQC, 6 layers, 1024 shots	Accuracy 98.5%	No	Not reported
Diviya et al. [29]	three tumor classes	4-qubit quantum convolution	Accuracy 97%	No	Not reported
This study	Kaggle (this dataset), four classes	2–10-qubit dressed VQC; quantum kernel	VQC never better than its control, apart from a small, inconsistent gain with two qubits	Yes, three controls, multiple seeds	Mirror-invariant audit; patient-grouped CV

Results as reported by the authors; accuracies refer to different datasets, tasks and splits and are not comparable with each other or with ours. PQC, parameterized quantum circuit; VQC, variational quantum circuit.

## Data Availability

Version 2 of the Brain Tumor MRI Dataset analyzed in this study is publicly available on Kaggle [9] (DOI 10.34740/KAGGLE/DSV/2645886; downloaded on 28 September 2026). The code of the dataset audit, the quantum circuit simulator and its self-tests, all experiments and the statistical analyses, together with the leakage-aware splits of Protocols 1–3 (duplicate and patient groups, clean and strict-clean flags, folds), the per-image audit annotations (duplicate pairs, figshare and SARTAJ matches, JPEG fingerprints and tumor masks), a checksum manifest of the 7200 images, the per-image predictions of all models and all result tables, are available at https://github.com/linhduongtuan/brain-tumor-qml (accessed on 15 September 2026). No image of the dataset is redistributed. The version of the repository that accompanies the published article will be archived on Zenodo together with the trained weights.

## Supplementary Materials

The following supporting information can be downloaded at https://www.mdpi.com/article/10.3390/diagnostics16213485/s1, Table S1: Frozen-feature benchmark under Protocol 3, all metrics; Table S2: Circuit ablations on frozen EfficientNet-B0 features (Protocol 3); Table S3: Data efficiency on frozen EfficientNet-B0 features (Protocol 3); Table S4: Accuracy of trained VQC heads under depolarizing noise and finite shots (Protocol 3); Table S5: Quantum versus classical kernel support vector machines (Protocol 3); Table S6: End-to-end models, accuracy in each fold and seed (Protocol 3); Table S7: Frozen-feature benchmark under Protocols 1 and 2 (first submission); Table S8: Sensitivity of the revised duplicate audit to the detection thresholds; Table S9: JPEG encoder fingerprints by source; Table S10: End-to-end models, class-specific sensitivity, specificity, F1 score and AUC (Protocol 3); Table S11: Submitted end-to-end runs re-analyzed by the link of each test image to the training partition; Table S12: Variance-corrected resampled *t*-tests of the frozen-feature heads per backbone; Table S13: Shortcut and source controls, all heads and backbones; Figure S1: Randomly sampled accepted and rejected duplicate pairs; Figure S2: Candidate label errors flagged by confident learning; Figure S3: Validation loss per epoch for the end-to-end EfficientNet-B0 heads (block B); Figure S4: Confusion matrices of the end-to-end models (Protocol 3); Figure S5: Examples of standardized and lesion-occluded images.

## Abbreviations

The following abbreviations are used in this manuscript:

AUC	Area under the receiver operating characteristic curve
CI	Confidence interval
CLAIM	Checklist for Artificial Intelligence in Medical Imaging
CNN	Convolutional neural network
CNOT	Controlled-NOT gate
CPU	Central processing unit
CV	Cross-validation
ECE	Expected calibration error
GPU	Graphics processing unit
Grad-CAM	Gradient-weighted class activation mapping
HQCNN	Hybrid quantum–classical convolutional neural network
MD5	Message-digest algorithm 5
MLP	Multilayer perceptron
MRI	Magnetic resonance imaging
NCC	Normalized cross-correlation
PCA	Principal component analysis
pHash	Perceptual hash
pp	Percentage points
RBF	Radial basis function
ReLU	Rectified linear unit
RGB	Red–green–blue
SD	Standard deviation
SVM	Support vector machine
ViT	Vision transformer
VQC	Variational quantum circuit
