# Supplementary material for: Does the Quantum Layer Help? A Leakage-Aware Benchmark of Hybrid Quantum–Classical Models for Brain Tumor MRI Classification
Source: Diagnostics (Basel). 2026 Oct 24;16(21):3485. doi: 10.3390/diagnostics16213485 (PMC13761405; doi:10.3390/diagnostics16213485)
Supplement: Supplementary file 1 [file diagnostics-16-03485-s001.zip › diagnostics-4644602-supplementary.pdf]

Supplementary Materials

Does the Quantum Layer Help? A Leakage-Aware Benchmark of Hybrid Quantum–Classical Models for Brain Tumor MRI Classification

Duong Tuan Linh, Pande Wikantyasa Maswita, Tony Sumaryada, Sitti Yani, Duong Thanh Tai *, Hiba Omer, Nissren Tamam and Abdelmoneim Sulieman. * Correspondence: dttai@ntt.edu.vn

Diagnostics **2026**, *16*, 3485. Revised version (first revision). Unless stated otherwise, all values are accuracies in percent under Protocol 3 (patient- and duplicate-grouped five-fold cross-validation; mean ± SD over folds). Every number can be recomputed from the code and per-image predictions at https://github.com/linhduongtuan/brain-tumor-qml.

Table S1. Frozen-feature benchmark under Protocol 3, all metrics.

Backbone	Head	Parameters	Quantum parameters	Accuracy	Balanced accuracy	Macro-F1	Macro-AUC	ECE
EfficientNet-B0	VQC head	7,750	36	82.0 ± 3.4	81.6 ± 3.4	81.6 ± 3.4	93.1 ± 2.0	0.058 ± 0.010
EfficientNet-B0	Classical control	7,756	0	86.4 ± 2.9	86.1 ± 2.9	86.0 ± 2.9	95.9 ± 1.9	0.053 ± 0.020
EfficientNet-B0	Deeper control	7,798	0	84.4 ± 2.6	84.1 ± 2.6	84.1 ± 2.6	95.5 ± 1.8	0.072 ± 0.030
EfficientNet-B0	Fourier surrogate	12,094	0	85.5 ± 3.3	85.2 ± 3.4	85.2 ± 3.4	95.9 ± 1.6	0.050 ± 0.029
EfficientNet-B0	Bottleneck only	7,714	0	87.6 ± 3.2	87.3 ± 3.3	87.3 ± 3.3	96.9 ± 1.9	0.047 ± 0.019
EfficientNet-B0	Linear probe	5,124	0	88.1 ± 3.0	87.8 ± 3.1	87.8 ± 3.1	97.3 ± 1.6	0.032 ± 0.022
EfficientNet-B0	MLP	328,964	0	87.9 ± 2.8	87.6 ± 2.9	87.6 ± 2.9	97.6 ± 1.5	0.041 ± 0.031
EfficientNet-B0	Untrained VQC	7,714	0	80.6 ± 4.5	80.1 ± 4.6	80.1 ± 4.6	91.6 ± 2.1	0.062 ± 0.012
DenseNet-121	VQC head	6,214	36	79.5 ± 3.5	79.2 ± 3.6	79.1 ± 3.6	92.2 ± 2.2	0.067 ± 0.025
DenseNet-121	Classical control	6,220	0	86.4 ± 3.1	86.1 ± 3.2	86.1 ± 3.2	96.2 ± 2.0	0.054 ± 0.017
DenseNet-121	Deeper control	6,262	0	85.5 ± 2.8	85.2 ± 2.8	85.2 ± 2.8	95.8 ± 1.5	0.065 ± 0.023
DenseNet-121	Fourier surrogate	10,558	0	85.4 ± 3.6	85.1 ± 3.6	85.1 ± 3.5	95.5 ± 2.2	0.050 ± 0.033
DenseNet-121	Bottleneck only	6,178	0	87.9 ± 3.6	87.6 ± 3.6	87.6 ± 3.6	97.0 ± 2.1	0.053 ± 0.018
DenseNet-121	Linear probe	4,100	0	88.1 ± 3.1	87.8 ± 3.1	87.7 ± 3.1	97.4 ± 1.6	0.036 ± 0.016
DenseNet-121	MLP	263,428	0	88.8 ± 3.2	88.6 ± 3.2	88.5 ± 3.2	97.8 ± 1.6	0.035 ± 0.028
DenseNet-121	Untrained VQC	6,178	0	78.1 ± 4.8	77.8 ± 4.6	77.9 ± 4.4	90.1 ± 3.6	0.094 ± 0.030
ResNet-50	VQC head	12,358	36	76.0 ± 4.0	75.6 ± 4.1	75.6 ± 4.1	90.3 ± 2.3	0.077 ± 0.023
ResNet-50	Classical control	12,364	0	83.1 ± 3.1	82.7 ± 3.1	82.8 ± 3.0	94.9 ± 1.8	0.071 ± 0.018
ResNet-50	Deeper control	12,406	0	81.3 ± 2.9	80.9 ± 2.9	80.9 ± 2.9	94.2 ± 1.7	0.085 ± 0.043
ResNet-50	Fourier surrogate	16,702	0	82.1 ± 2.6	81.7 ± 2.7	81.7 ± 2.6	94.4 ± 1.5	0.056 ± 0.033
ResNet-50	Bottleneck only	12,322	0	85.5 ± 2.5	85.1 ± 2.6	85.1 ± 2.5	96.3 ± 1.6	0.047 ± 0.016
ResNet-50	Linear probe	8,196	0	86.0 ± 2.3	85.7 ± 2.4	85.6 ± 2.4	96.6 ± 1.5	0.029 ± 0.013
ResNet-50	MLP	525,572	0	86.3 ± 2.1	86.0 ± 2.2	85.9 ± 2.2	96.9 ± 1.3	0.038 ± 0.018

Backbone	Head	Parameters	Quantum parameters	Accuracy	Balanced accuracy	Macro-F1	Macro-AUC	ECE
ResNet-50	Untrained VQC	12,322	0	69.7 ± 3.0	69.3 ± 2.8	69.1 ± 2.9	86.9 ± 2.0	0.094 ± 0.011
MobileNetV2	VQC head	7,750	36	78.5 ± 2.1	78.1 ± 2.1	77.9 ± 2.4	91.6 ± 1.3	0.064 ± 0.009
MobileNetV2	Classical control	7,756	0	84.3 ± 3.9	84.0 ± 4.0	84.0 ± 3.9	95.1 ± 2.2	0.067 ± 0.018
MobileNetV2	Deeper control	7,798	0	83.7 ± 3.7	83.3 ± 3.8	83.4 ± 3.8	95.2 ± 1.7	0.083 ± 0.024
MobileNetV2	Fourier surrogate	12,094	0	84.2 ± 2.9	84.0 ± 3.0	84.0 ± 2.9	95.3 ± 2.2	0.054 ± 0.027
MobileNetV2	Bottleneck only	7,714	0	86.4 ± 3.6	86.1 ± 3.7	86.1 ± 3.6	96.7 ± 1.9	0.051 ± 0.015
MobileNetV2	Linear probe	5,124	0	87.1 ± 3.6	86.8 ± 3.7	86.7 ± 3.7	97.1 ± 1.9	0.035 ± 0.024
MobileNetV2	MLP	328,964	0	87.5 ± 3.7	87.2 ± 3.8	87.2 ± 3.8	97.4 ± 1.7	0.040 ± 0.032
MobileNetV2	Untrained VQC	7,714	0	77.0 ± 5.8	76.6 ± 5.8	76.5 ± 6.0	90.2 ± 2.6	0.070 ± 0.039

Mean ± SD over the five folds of the per-fold mean over three seeds (one seed for the untrained VQC). AUC, area under the receiver operating characteristic curve; ECE, expected calibration error (15 bins); MLP, multilayer perceptron; VQC, variational quantum circuit.

Table S2. Circuit ablations on frozen EfficientNet-B0 features (Protocol 3, seed 0).

Head	Width (qubits)	Layers	Entanglement	Re-uploading	Accuracy	Balanced accuracy	Training time (s)[a]	Epochs	Folds
VQC	2	2	ring	no	83.3 ± 2.7	83.0 ± 2.8	15	39.8	5
VQC	4	2	ring	no	83.6 ± 2.4	83.3 ± 2.5	19	27.4	5
VQC	6	2	ring	no	83.5 ± 3.6	83.1 ± 3.6	11	25.8	5
VQC	8	2	ring	no	80.8 ± 4.6	80.4 ± 4.6	19	24.8	5
VQC	10	2	ring	no	81.4 ± 2.5	81.0 ± 2.6	141	26.2	5
Classical control	2	–	–	–	82.0 ± 2.0	81.7 ± 2.1	4	31.8	5
Classical control	4	–	–	–	85.0 ± 3.8	84.6 ± 3.8	1	20.4	5
Classical control	6	–	–	–	86.7 ± 4.0	86.3 ± 4.1	1	20.4	5
Classical control	8	–	–	–	86.6 ± 3.0	86.2 ± 3.0	1	16.2	5
Classical control	10	–	–	–	85.9 ± 3.0	85.6 ± 3.1	1	14.4	5
Bottleneck only	2	–	–	–	84.2 ± 2.9	83.9 ± 2.9	2	19.8	5
Bottleneck only	4	–	–	–	85.6 ± 3.4	85.2 ± 3.4	1	15.8	5
Bottleneck only	6	–	–	–	87.3 ± 3.3	87.0 ± 3.3	1	18.2	5
Bottleneck only	8	–	–	–	87.9 ± 3.5	87.5 ± 3.6	1	16.2	5
Bottleneck only	10	–	–	–	87.2 ± 3.4	86.9 ± 3.5	1	14.2	5
VQC	6	1	ring	no	83.0 ± 4.0	82.7 ± 4.0	6	28.6	5
VQC	6	4	ring	no	85.0 ± 2.8	84.7 ± 2.8	15	23.8	5
VQC	6	6	ring	no	84.8 ± 4.6	84.5 ± 4.6	42	46.4	5
VQC	6	2	no	no	86.4 ± 2.8	86.1 ± 2.9	8	22.0	5
VQC	6	2	ring	yes	78.2 ± 4.7	77.9 ± 4.7	17	34.6	5
Deeper control	6	–	–	–	83.7 ± 3.4	83.4 ± 3.4	1	16.6	5
Fourier surrogate	6	–	–	–	85.4 ± 3.0	85.1 ± 3.1	2	12.6	5

Head	Width (qubits)	Layers	Entanglement	Re-uploading	Accuracy	Balanced accuracy	Training time (s)[a]	Epochs	Folds
Linear probe	–	–	–	–	88.0 ± 2.8	87.7 ± 2.9	1	14.4	5
MLP	–	–	–	–	88.1 ± 2.8	87.8 ± 2.8	2	11.8	5
VQC (untrained)	6	2	ring	no	80.6 ± 4.5	80.1 ± 4.6	7	38.0	5

Mean ± SD over the five folds. [a] Mean CPU time of one training run at the selected learning rate (one CPU thread per run; two runs in parallel on the two cores).

Table S3. Data efficiency on frozen EfficientNet-B0 features (Protocol 3, seed 0).

Training data (%)	Training images (mean)	VQC head	Classical control	Linear probe	MLP
1	115	38.6 ± 5.7	62.3 ± 9.0	65.5 ± 6.7	62.3 ± 12.1
2	160	43.3 ± 8.2	68.9 ± 6.9	70.1 ± 4.4	69.6 ± 5.0
5	302	54.6 ± 8.7	75.7 ± 5.7	78.2 ± 4.1	78.1 ± 4.1
10	557	65.7 ± 5.8	80.4 ± 2.6	81.3 ± 1.8	81.1 ± 2.4
25	1254	76.3 ± 4.2	84.6 ± 3.1	84.9 ± 2.5	84.1 ± 2.8
50	2523	80.4 ± 4.7	85.7 ± 4.3	86.2 ± 2.0	85.9 ± 2.6
100	4937	83.5 ± 3.6	86.7 ± 4.0	88.0 ± 2.8	88.1 ± 2.8

Accuracy, mean ± SD over the five folds. Training folds were subsampled within each class by whole patient and duplicate groups; validation sets were unchanged.

Table S4. Accuracy of trained VQC heads under depolarizing noise and finite shots (frozen EfficientNet-B0 features, Protocol 3).

Condition	2 qubits, 2 layers	4 qubits, 2 layers	6 qubits, 1 layer	6 qubits, 2 layers	6 qubits, 4 layers	6 qubits, 6 layers
Depolarizing, p = 0	83.3	83.6	83.0	83.5	85.0	84.8
Depolarizing, p = 0.001	83.3	83.6	83.0	83.6	85.0	84.8
Depolarizing, p = 0.003	83.3	83.6	83.0	83.6	85.0	84.8
Depolarizing, p = 0.01	83.3	83.6	83.0	83.5	85.1	84.3
Depolarizing, p = 0.03	83.2	83.5	82.7	83.3	84.6	62.7
Depolarizing, p = 0.1	83.0	83.5	75.1	70.2	31.3	24.0
16 shots	83.1	83.4	82.3	82.0	84.5	80.5
64 shots	83.3	83.5	82.7	83.1	84.9	84.0
256 shots	83.3	83.6	82.9	83.5	85.0	84.5
1024 shots	83.3	83.6	83.0	83.6	85.0	84.7
4096 shots	83.3	83.6	83.1	83.6	85.0	84.7
p = 0.01, 1024 shots	83.2	83.5	82.9	83.6	85.3	84.3

Mean accuracy over the five folds; heads were trained without noise and evaluated on their test folds.

Table S5. Quantum versus classical kernel support vector machines on principal components of frozen EfficientNet-B0 features (Protocol 3).

Components (qubits)	ZZ fidelity kernel	RBF kernel	Difference, pp (range over folds)	Folds ZZ better	Selected ZZ bandwidth (folds)	Off-diagonal kernel variance (ZZ, selected)	Time (s)
4	72.8 ± 1.7	73.3 ± 1.4	−0.53 (−1.14 to +0.50)	1/5	0.03 (3), 0.1 (2)	0.0247	14
6	77.7 ± 1.2	78.1 ± 2.0	−0.33 (−1.86 to +0.57)	2/5	0.03 (3), 0.1 (2)	0.0237	9
8	79.7 ± 1.3	80.8 ± 1.8	−1.04 (−1.50 to −0.14)	0/5	0.03 (5)	0.0316	13
10	80.9 ± 2.8	81.5 ± 2.5	−0.56 (−2.93 to +0.86)	3/5	0.03 (4), 0.1 (1)	0.0318	40

Accuracy, mean ± SD over the five folds. The ZZ bandwidth and C, and the RBF bandwidth and C, were selected on validation accuracy in each fold; the off-diagonal variance at every fixed bandwidth is shown in Figure 7b. RBF, radial basis function.

Table S6. End-to-end models under Protocol 3: accuracy in each fold and seed.

Block	Model	Seed	Fold 1	Fold 2	Fold 3	Fold 4	Fold 5	Mean ± SD	Epochs (mean)
A	EfficientNet-B0, linear head	0	94.9	94.9	88.5	97.8	96.4	94.5 ± 3.6	18.0
A	ResNet-50	0	95.1	94.1	88.6	97.6	95.9	94.3 ± 3.4	17.2
A	EfficientNet-B0 + classical control	0	95.9	94.4	89.5	95.6	95.6	94.2 ± 2.7	17.6
A	EfficientNet-B0 + VQC head	0	95.4	93.8	88.6	98.4	94.4	94.1 ± 3.6	20.8
A	HQCNN	0	67.7	63.2	60.3	75.4	73.0	67.9 ± 6.3	18.6
A	HQCNN classical control	0	71.6	80.6	77.7	68.3	83.1	76.2 ± 6.2	19.0
B	EfficientNet-B0, linear head	0	95.4	95.1	89.9	98.1	95.5	94.8 ± 3.0	26.2
B	EfficientNet-B0, linear head	1	95.2	94.9	90.2	95.8	95.4	94.3 ± 2.3	20.2
B	EfficientNet-B0 + classical control	0	94.6	95.0	90.0	96.6	95.0	94.2 ± 2.5	22.6
B	EfficientNet-B0 + classical control	1	95.5	96.3	89.6	97.0	95.8	94.8 ± 3.0	29.4
B	EfficientNet-B0 + VQC head	0	91.6	94.9	89.5	97.1	93.6	93.4 ± 2.9	33.0
B	EfficientNet-B0 + VQC head	1	94.9	95.4	89.8	95.9	95.8	94.3 ± 2.5	44.6
B	EfficientNet-B0 + deeper control	0	95.1	95.1	90.1	96.7	95.4	94.5 ± 2.6	28.8
B	EfficientNet-B0 + deeper control	1	94.1	95.2	88.9	95.4	94.8	93.7 ± 2.7	29.4
B	EfficientNet-B0 + Fourier surrogate	0	95.2	94.2	87.8	96.1	96.2	93.9 ± 3.5	24.8
B	EfficientNet-B0 + Fourier surrogate	1	93.7	94.8	89.3	96.6	95.8	94.0 ± 2.9	19.8
C	EfficientNet-B0, linear head	0	94.6	94.9	88.8	98.1	95.3	94.3 ± 3.4	14.8
C	EfficientNet-B0 + classical control	0	94.9	95.4	91.1	97.8	94.6	94.7 ± 2.4	21.0
C	EfficientNet-B0 + VQC head	0	94.6	93.1	88.4	97.2	94.7	93.6 ± 3.3	24.2

Block A, recipe of the first submission; block B, convergence-based training (learning rate 10^{-3} selected for every head); block C, standardized images.

Table S7. Frozen-feature benchmark of the first submission (Protocol 2, grouped five-fold cross-validation; official partition).

Backbone	Head	Parameters	Quantum parameters	Accuracy	Balanced accuracy	Macro F1	Macro AUC	ECE	Official test accuracy	Clean-subset balanced accuracy	Inflation, pp
EfficientNet-B0	6-qubit VQC head	7750	36	91.3 ± 0.9	91.1 ± 1.0	91.1 ± 0.9	98.2 ± 0.2	3.1 ± 0.6	88.7 ± 1.7	87.9 ± 1.9	+0.7
EfficientNet-B0	Classical control (6→6 tanh)	7756	0	92.7 ± 0.9	92.6 ± 1.0	92.6 ± 0.9	98.3 ± 0.4	2.5 ± 0.7	92.0 ± 0.8	91.9 ± 0.8	+0.1
EfficientNet-B0	Bottleneck only (no middle layer)	7714	0	92.6 ± 0.6	92.5 ± 0.6	92.5 ± 0.6	98.7 ± 0.1	2.7 ± 0.6	92.5 ± 0.3	92.5 ± 0.2	+0.1
EfficientNet-B0	Linear probe	5124	0	92.8 ± 0.4	92.7 ± 0.4	92.7 ± 0.4	98.8 ± 0.2	2.5 ± 0.8	92.3 ± 0.2	92.1 ± 0.2	+0.3
EfficientNet-B0	MLP (256 hidden)	328964	0	93.8 ± 0.7	93.7 ± 0.8	93.7 ± 0.8	99.2 ± 0.2	2.8 ± 0.5	92.9 ± 0.1	92.5 ± 0.3	+0.4
EfficientNet-B0	Untrained (frozen) VQC	7714	0	88.7 ± 0.8	88.6 ± 0.8	88.6 ± 0.8	97.2 ± 0.2	9.3 ± 2.4	89.7	88.7	+1.0
DenseNet-121	6-qubit VQC head	6214	36	86.4 ± 2.0	86.1 ± 2.0	86.1 ± 2.0	95.4 ± 1.2	3.9 ± 0.5	86.4 ± 1.7	85.2 ± 1.4	+1.2
DenseNet-121	Classical control (6→6 tanh)	6220	0	91.5 ± 0.5	91.3 ± 0.5	91.3 ± 0.5	98.2 ± 0.1	3.1 ± 0.8	90.7 ± 0.7	89.5 ± 0.6	+1.2
DenseNet-121	Bottleneck only (no middle layer)	6178	0	92.1 ± 0.3	92.0 ± 0.3	91.9 ± 0.3	98.5 ± 0.3	1.9 ± 0.7	90.8 ± 0.1	89.7 ± 0.2	+1.0
DenseNet-121	Linear probe	4100	0	92.1 ± 0.7	91.8 ± 0.7	91.8 ± 0.7	98.8 ± 0.1	1.7 ± 0.1	90.7 ± 0.2	89.8 ± 0.2	+0.9
DenseNet-121	MLP (256 hidden)	263428	0	93.5 ± 0.4	93.4 ± 0.4	93.4 ± 0.4	99.2 ± 0.0	3.0 ± 0.6	92.2 ± 0.3	91.4 ± 0.2	+0.8
DenseNet-121	Untrained (frozen) VQC	6178	0	84.5 ± 1.6	84.2 ± 1.6	84.2 ± 1.6	93.8 ± 0.6	4.9 ± 0.9	77.3	80.7	−3.4
ResNet-50	6-qubit VQC head	12358	36	80.2 ± 2.2	79.7 ± 2.2	79.6 ± 2.3	93.0 ± 0.9	5.0 ± 1.4	77.2 ± 4.7	78.2 ± 4.4	−0.9
ResNet-50	Classical control (6→6 tanh)	12364	0	88.5 ± 1.2	88.3 ± 1.2	88.3 ± 1.2	97.3 ± 0.6	3.7 ± 0.8	86.2 ± 2.3	84.5 ± 2.7	+1.7
ResNet-50	Bottleneck only (no middle layer)	12322	0	89.6 ± 0.4	89.3 ± 0.4	89.3 ± 0.4	98.0 ± 0.4	3.0 ± 0.9	89.1 ± 0.4	87.9 ± 1.0	+1.3
ResNet-50	Linear probe	8196	0	89.5 ± 0.7	89.2 ± 0.7	89.2 ± 0.8	98.2 ± 0.3	2.8 ± 0.5	89.6 ± 0.3	88.6 ± 0.4	+1.0
ResNet-50	MLP (256 hidden)	525572	0	90.8 ± 0.9	90.7 ± 1.0	90.6 ± 1.0	98.5 ± 0.3	3.2 ± 0.4	89.3 ± 0.4	87.8 ± 1.3	+1.5
ResNet-50	Untrained (frozen) VQC	12322	0	79.1 ± 2.4	78.6 ± 2.3	78.3 ± 2.3	91.4 ± 0.5	6.2 ± 2.1	78.4	76.2	+2.2
MobileNetV2	6-qubit VQC head	7750	36	87.7 ± 1.5	87.4 ± 1.6	87.4 ± 1.6	97.0 ± 0.3	3.9 ± 0.7	85.6 ± 2.7	83.7 ± 2.0	+1.9
MobileNetV2	Classical control (6→6 tanh)	7756	0	90.5 ± 0.8	90.3 ± 0.8	90.3 ± 0.8	97.9 ± 0.4	3.0 ± 1.2	89.5 ± 1.2	87.8 ± 1.8	+1.7
MobileNetV2	Bottleneck only (no middle layer)	7714	0	90.5 ± 1.2	90.3 ± 1.3	90.3 ± 1.3	98.3 ± 0.2	2.6 ± 0.7	91.1 ± 0.7	89.5 ± 1.3	+1.6
MobileNetV2	Linear probe	5124	0	91.0 ± 1.7	90.8 ± 1.7	90.8 ± 1.7	98.5 ± 0.2	2.2 ± 1.1	91.1 ± 0.3	89.5 ± 0.3	+1.6
MobileNetV2	MLP (256 hidden)	328964	0	92.4 ± 1.2	92.2 ± 1.2	92.2 ± 1.2	98.9 ± 0.2	3.3 ± 1.0	92.2 ± 0.4	91.7 ± 0.5	+0.5
MobileNetV2	Untrained (frozen) VQC	7714	0	83.3 ± 1.8	82.9 ± 1.8	82.9 ± 1.9	94.7 ± 1.0	11.8 ± 2.5	84.3	80.3	+4.0

As reported in the first submission (Protocol 2 mean ± SD over folds, in percent; ECE in percent; inflation = official test accuracy minus balanced accuracy on the clean subset of the first-submission audit, in pp). Protocol 2 groups only the duplicates of the original audit, so its accuracies are inflated by leakage (Section 3.8).

Table S8. Sensitivity of the duplicate audit to the detection thresholds.

Invariant to reflections and rotations	Hamming distance ≤	NCC ≥	Pairs	Training–test pairs	Linked non-augmented test images	Clean test images	Groups with ≥ 2 images	Largest group	Links across Protocol 2 folds	Links across Protocol 3 folds
no	4	0.90	5,497	1,918	656	841	1114	28	366	4
no	4	0.95	4,952	1,764	555	942	864	28	0	0
no	4	0.98	4,216	1,547	467	1030	691	28	0	0
no	6	0.90	7,099	2,478	811	686	1286	28	792	26
no	6	0.95	5,932	2,147	626	871	935	28	0	0
no	6	0.98	4,547	1,677	477	1020	685	28	0	0
no	8	0.90	8,409	2,875	928	569	1348	35	1,254	87
no	8	0.95	6,547	2,347	665	832	960	35	0	0
no	8	0.98	4,697	1,725	486	1011	683	28	0	0
no	10	0.90	9,255	3,132	1000	497	1335	55	1,800	140
no	10	0.95	6,778	2,425	688	809	964	35	96	0
no	10	0.98	4,728	1,734	487	1010	684	28	9	0
no	12	0.90	9,828	3,262	1029	468	1293	170	2,111	202
no	12	0.95	6,849	2,438	691	806	960	35	134	1
no	12	0.98	4,732	1,734	487	1010	684	28	9	0
no	14	0.90	10,193	3,374	1045	452	1254	372	2,339	305
no	14	0.95	6,865	2,444	692	805	955	35	143	1
no	14	0.98	4,732	1,734	487	1010	684	28	9	0
yes	4	0.90	20,393	8,149	1173	324	1764	81	12,651	5
yes	4	0.95	19,330	7,794	1131	366	1943	81	11,907	0
yes	4	0.98	17,217	7,021	1086	411	2076	81	10,698	0
yes	6	0.90	24,915	9,780	1224	273	1533	81	15,477	38
yes	6	0.95	22,623	9,014	1163	334	1869	81	13,823	0
yes	6	0.98	18,403	7,433	1087	410	2070	81	11,387	0
yes	8	0.90	28,350	10,909	1264	233	1341	83	17,594	154
yes	8	0.95	24,623	9,699	1168	329	1821	83	14,919	0
yes	8	0.98	18,941	7,619	1087	410	2065	81	11,684	0
yes	10	0.90	30,354	11,554	1282	215	1196	144	19,077	256
yes	10	0.95	25,281	9,908	1173	324	1788	83	15,358	0
yes	10	0.98	19,058	7,655	1088	409	2066	81	11,762	0
yes	12	0.90	31,666	11,924	1297	200	1107	453	19,996	395
yes	12	0.95	25,471	9,949	1173	324	1773	83	15,494	2
yes	12	0.98	19,069	7,657	1088	409	2066	81	11,768	0
yes	14	0.90	32,572	12,241	1299	198	1066	601	20,683	624
yes	14	0.95	25,508	9,960	1174	323	1771	83	15,520	2
yes	14	0.98	19,069	7,657	1088	409	2066	81	11,768	0

The thresholds used in the paper are a Hamming distance of 8 bits and NCC ≥ 0.95 with invariance. Augmented copies are linked to their source image in every setting. Links across Protocol 3 folds count pairs of non-augmented images in different Protocol 3 folds; under the thresholds used, there are none by construction. NCC, normalized cross-correlation.

Table S9. JPEG encoder fingerprints by source (12 most frequent of all 7200 images).

Color mode, chroma subsampling, quantization table	Figshare-derived tumor	SARTAJ tumor (other)	Out-of-source glioma	No tumor
L, n/a, table 2df19496	3038	0	0	24
RGB, 4:2:0, table 92e341e6	1762	145	0	92
RGB, 4:2:0, table 9f8a9109	19	172	0	1260
RGB, 4:4:4, table 92e341e6	27	156	76	356
RGB, 4:2:0, table 9158c468	0	5	0	15
RGBA, n/a, table none	0	0	0	3
RGB, 4:2:0, table b871b72b	0	0	0	3
RGB, 4:2:0, table 3541f993	0	0	0	2
L, n/a, table a5268808	0	0	0	1
L, n/a, table 959d096a	0	0	0	1
RGB, 4:2:0, table 171b3d31	0	0	0	1
RGB, 4:2:0, table 1a1346ed	0	0	0	1

Counts of images. Quantization tables are identified by the first eight hexadecimal digits of the MD5 hash of the tables. SARTAJ tumor (other): non-figshare tumor images other than the 76 out-of-source glioma images; all non-augmented ones match SARTAJ images.

Table S10. End-to-end models under Protocol 3: class-specific sensitivity, specificity, F1 score and AUC.

Block	Model	Class	Sensitivity, % (95% CI)	Specificity, % (95% CI)	F1, %	AUC, %
A	EfficientNet-B0, linear head	Glioma	93.7 (90.5–96.0)	97.9 (96.6–99.0)	93.8	98.7
A	EfficientNet-B0, linear head	Meningioma	91.9 (86.6–95.7)	96.6 (95.1–97.8)	90.4	98.0
A	EfficientNet-B0, linear head	Pituitary tumor	94.1 (89.8–97.4)	98.9 (97.3–99.7)	95.4	98.9
A	EfficientNet-B0, linear head	No tumor	98.1 (96.0–99.4)	99.3 (98.9–99.6)	98.0	99.8
A	ResNet-50	Glioma	94.6 (91.8–96.5)	97.5 (96.0–98.7)	93.8	98.7
A	ResNet-50	Meningioma	91.1 (85.2–95.5)	96.8 (95.5–97.8)	90.2	97.8
A	ResNet-50	Pituitary tumor	93.3 (89.5–96.3)	99.2 (97.7–99.9)	95.3	99.2
A	ResNet-50	No tumor	97.8 (95.2–99.5)	98.9 (98.4–99.4)	97.4	99.7
A	EfficientNet-B0 + classical control	Glioma	93.6 (90.8–95.6)	97.5 (96.0–98.6)	93.2	98.4
A	EfficientNet-B0 + classical control	Meningioma	90.9 (85.4–95.0)	96.5 (95.1–97.5)	89.6	96.6
A	EfficientNet-B0 + classical control	Pituitary tumor	93.1 (88.6–96.6)	99.0 (97.5–99.9)	95.0	97.6
A	EfficientNet-B0 + classical control	No tumor	98.8 (97.7–99.6)	99.3 (98.9–99.6)	98.4	99.9
A	HQCNN	Glioma	88.7 (85.0–91.7)	74.3 (68.8–79.4)	67.5	88.4
A	HQCNN	Meningioma	13.8 (10.4–17.5)	91.7 (89.6–93.6)	19.4	77.2
A	HQCNN	Pituitary tumor	72.6 (64.9–79.7)	96.7 (95.5–97.8)	79.7	96.1
A	HQCNN	No tumor	90.4 (86.5–93.5)	94.4 (93.0–95.6)	87.6	97.7
A	HQCNN classical control	Glioma	71.7 (65.6–77.3)	91.2 (88.1–94.1)	72.8	91.8

Block	Model	Class	Sensitivity, % (95% CI)	Specificity, % (95% CI)	F1, %	AUC, %
A	HQCNN classical control	Meningioma	52.2 (42.3–61.9)	89.6 (86.9–91.8)	55.6	82.9
A	HQCNN classical control	Pituitary tumor	87.8 (82.6–92.5)	92.5 (90.0–94.7)	83.9	95.6
A	HQCNN classical control	No tumor	90.6 (86.4–93.8)	95.2 (94.0–96.2)	88.5	97.7
A	EfficientNet-B0 + VQC head	Glioma	92.5 (89.2–94.9)	98.0 (97.1–98.8)	93.4	98.5
A	EfficientNet-B0 + VQC head	Meningioma	91.6 (86.1–95.6)	96.4 (94.9–97.6)	89.9	96.9
A	EfficientNet-B0 + VQC head	Pituitary tumor	93.9 (90.1–96.9)	98.8 (97.2–99.7)	95.2	98.9
A	EfficientNet-B0 + VQC head	No tumor	98.1 (96.1–99.4)	99.0 (98.5–99.3)	97.5	99.6
B	EfficientNet-B0, linear head	Glioma	94.7 (91.8–97.1)	97.9 (96.6–98.9)	94.3	99.2
B	EfficientNet-B0, linear head	Meningioma	92.3 (86.6–96.3)	97.3 (96.3–98.3)	91.7	98.6
B	EfficientNet-B0, linear head	Pituitary tumor	95.4 (92.3–97.8)	99.0 (97.5–99.8)	96.3	99.5
B	EfficientNet-B0, linear head	No tumor	97.8 (95.6–99.3)	99.3 (98.8–99.6)	97.8	99.9
B	EfficientNet-B0 + deeper control	Glioma	94.0 (91.1–96.4)	97.3 (95.9–98.4)	93.2	98.7
B	EfficientNet-B0 + deeper control	Meningioma	91.0 (85.2–95.4)	97.1 (95.7–98.3)	90.7	97.0
B	EfficientNet-B0 + deeper control	Pituitary tumor	94.3 (90.1–97.5)	99.2 (97.6–99.9)	95.8	98.4
B	EfficientNet-B0 + deeper control	No tumor	98.3 (97.0–99.2)	99.1 (98.6–99.5)	97.8	99.9
B	EfficientNet-B0 + classical control	Glioma	94.8 (92.4–96.7)	98.5 (97.3–99.3)	95.2	99.5
B	EfficientNet-B0 + classical control	Meningioma	93.0 (87.7–96.7)	96.9 (95.5–98.0)	91.4	97.6
B	EfficientNet-B0 + classical control	Pituitary tumor	94.5 (90.3–97.6)	99.1 (97.6–99.9)	95.8	98.5
B	EfficientNet-B0 + classical control	No tumor	98.2 (96.4–99.4)	99.2 (98.8–99.6)	98.0	99.9
B	EfficientNet-B0 + Fourier surrogate	Glioma	94.8 (92.2–96.8)	97.5 (96.0–98.5)	93.9	98.8
B	EfficientNet-B0 + Fourier surrogate	Meningioma	90.2 (84.4–94.6)	97.4 (96.2–98.4)	90.6	96.8
B	EfficientNet-B0 + Fourier surrogate	Pituitary tumor	95.2 (91.3–98.2)	99.0 (97.5–99.8)	96.1	99.0
B	EfficientNet-B0 + Fourier surrogate	No tumor	98.6 (97.0–99.6)	99.2 (98.8–99.6)	98.2	99.9
B	EfficientNet-B0 + VQC head	Glioma	95.0 (92.8–96.7)	98.0 (96.9–98.8)	94.6	99.3
B	EfficientNet-B0 + VQC head	Meningioma	92.2 (86.6–96.3)	96.9 (95.7–97.9)	91.0	98.1
B	EfficientNet-B0 + VQC head	Pituitary tumor	94.6 (91.5–97.3)	99.1 (97.7–99.9)	96.0	98.9
B	EfficientNet-B0 + VQC head	No tumor	97.9 (95.7–99.3)	99.5 (99.1–99.7)	98.2	99.7
C	EfficientNet-B0, linear head	Glioma	91.3 (87.5–94.4)	98.3 (97.3–99.1)	93.1	98.3
C	EfficientNet-B0, linear head	Meningioma	92.0 (86.4–95.9)	96.4 (95.0–97.6)	90.2	97.8
C	EfficientNet-B0, linear head	Pituitary tumor	95.8 (93.1–97.7)	98.7 (97.0–99.6)	96.0	99.1
C	EfficientNet-B0, linear head	No tumor	97.9 (95.7–99.5)	99.1 (98.6–99.4)	97.6	99.8
C	EfficientNet-B0 + classical control	Glioma	93.9 (90.9–96.2)	97.8 (96.6–98.8)	93.9	98.8
C	EfficientNet-B0 + classical control	Meningioma	91.6 (86.0–95.8)	97.1 (95.9–98.1)	90.9	96.5
C	EfficientNet-B0 + classical control	Pituitary tumor	94.7 (91.4–97.4)	98.7 (97.1–99.6)	95.5	98.9
C	EfficientNet-B0 + classical control	No tumor	98.3 (96.9–99.3)	99.4 (99.0–99.7)	98.3	99.7
C	EfficientNet-B0 + VQC head	Glioma	93.4 (90.7–95.6)	97.4 (96.0–98.6)	93.0	98.4
C	EfficientNet-B0 + VQC head	Meningioma	90.3 (84.4–94.6)	96.3 (94.9–97.5)	89.1	96.3
C	EfficientNet-B0 + VQC head	Pituitary tumor	93.3 (89.1–96.5)	98.5 (97.0–99.5)	94.4	98.4
C	EfficientNet-B0 + VQC head	No tumor	97.1 (94.5–98.8)	99.3 (98.9–99.6)	97.5	99.1

Out-of-fold predictions for the 6997 non-augmented images, averaged over the runs of each model; 95% CIs from a cluster bootstrap over patient and duplicate groups (2000 resamples). Confusion matrices are shown in Figure S4.

Table S11. Submitted end-to-end runs on the official partition, re-analyzed with the revised audit.

(a) Accuracy (%, 95% CI) and class sensitivity (%) on the official test set and its subsets.

Model	Test set	Accuracy, % (95% CI)	Glioma	Meningioma	Pituitary tumor	No tumor
EfficientNet-B0	official test (1600)	95.5 (94.3–96.7)	83.6	98.9	99.7	100.0
EfficientNet-B0	clean subset, first submission (832)	92.0 (89.5–94.1)	81.9	99.0	99.5	100.0
EfficientNet-B0	strict-clean subset (87)	29.5 (19.3–39.9)	18.0	93.9	100.0	–
ResNet-50	official test (1600)	95.4 (94.2–96.5)	83.8	99.0	98.8	99.9
ResNet-50	clean subset, first submission (832)	92.0 (89.6–94.1)	82.2	99.6	98.8	100.0
ResNet-50	strict-clean subset (87)	33.7 (22.6–44.4)	22.1	100.0	100.0	–
DenseNet-121	official test (1600)	95.6 (94.3–96.7)	84.4	98.4	99.4	100.0
DenseNet-121	clean subset, first submission (832)	92.4 (90.2–94.4)	82.9	99.0	99.5	100.0
DenseNet-121	strict-clean subset (87)	32.6 (22.0–44.4)	21.2	97.0	100.0	–
MobileNetV2	official test (1600)	95.8 (94.5–96.8)	84.9	98.8	99.6	100.0
MobileNetV2	clean subset, first submission (832)	92.7 (90.4–94.6)	83.4	99.2	99.5	100.0
MobileNetV2	strict-clean subset (87)	37.2 (25.8–49.4)	26.1	100.0	100.0	–
ConvNeXt-T	official test (1600)	95.4 (94.2–96.4)	84.8	97.9	99.0	99.9
ConvNeXt-T	clean subset, first submission (832)	92.1 (89.9–94.1)	83.2	97.6	99.2	100.0
ConvNeXt-T	strict-clean subset (87)	35.2 (25.2–45.3)	23.9	100.0	100.0	–
ViT-S/16	official test (1600)	94.4 (93.1–95.6)	83.0	97.2	97.9	99.5
ViT-S/16	clean subset, first submission (832)	90.8 (88.5–92.9)	81.4	97.2	97.9	98.7
ViT-S/16	strict-clean subset (87)	32.6 (22.7–42.8)	21.2	97.0	100.0	–
EfficientNet-B0 + VQC head	official test (1600)	95.5 (94.2–96.6)	84.2	98.2	99.5	99.8
EfficientNet-B0 + VQC head	clean subset, first submission (832)	92.2 (90.0–94.2)	82.7	99.0	99.3	100.0
EfficientNet-B0 + VQC head	strict-clean subset (87)	33.7 (23.4–44.1)	23.0	93.9	100.0	–
EfficientNet-B0 + classical control	official test (1600)	95.6 (94.3–96.7)	84.5	98.1	99.8	100.0
EfficientNet-B0 + classical control	clean subset, first submission (832)	92.3 (90.1–94.2)	83.0	98.2	99.6	100.0
EfficientNet-B0 + classical control	strict-clean subset (87)	31.0 (21.1–40.7)	21.6	81.8	100.0	–
HQCNN	official test (1600)	78.4 (74.8–81.7)	60.7	67.0	88.2	97.8
HQCNN	clean subset, first submission (832)	74.2 (68.8–79.0)	57.9	85.4	86.0	93.6
HQCNN	strict-clean subset (87)	7.3 (2.7–13.3)	4.1	12.1	100.0	–
HQCNN classical control	official test (1600)	80.5 (77.1–83.7)	63.2	67.2	92.7	98.8
HQCNN classical control	clean subset, first submission (832)	77.0 (72.3–81.2)	60.4	84.4	91.3	96.2
HQCNN classical control	strict-clean subset (87)	6.5 (1.9–12.6)	4.5	3.0	100.0	–

(b) Accuracy (%) by the link of each test image to the training partition (three seeds pooled).

Model	All test images	Copy of the image in training	Same patient in training, no copy	No link to training	of which out-of-source glioma
EfficientNet-B0	95.5	99.6	98.2	29.5	18.0
ResNet-50	95.4	99.3	97.1	33.7	22.1
DenseNet-121	95.6	99.3	98.6	32.6	21.2
MobileNetV2	95.8	99.4	97.8	37.2	26.1
ConvNeXt-T	95.4	99.0	98.0	35.2	23.9
ViT-S/16	94.4	98.1	97.0	32.6	21.2
EffNet-B0 + VQC	95.5	99.2	98.0	33.7	23.0
EffNet-B0 + classical	95.6	99.5	98.4	31.0	21.6
HQCNN	78.4	83.6	76.6	7.3	4.1
HQCNN control	80.5	85.4	81.0	6.5	4.5

Three seeds per model, trained on the official training partition (Protocol 1, first submission). Class columns give sensitivity (%). The strict-clean subset contains 74 glioma (all out-of-source), 11 meningioma and 2 pituitary tumor images and no no-tumor image; the clean subset of the first submission contains 26 no-tumor images. 95% CIs from a cluster bootstrap over patient and duplicate groups.

Table S12. Variance-corrected resampled t-tests of the frozen-feature VQC head against each classical head, per backbone (Protocol 3).

Comparator	Backbone	VQC minus comparator (pp)	Corrected t	p	Fold–seed pairs
Classical control	DenseNet-121	−6.87	−4.79	0.00029	15
Classical control	EfficientNet-B0	−4.44	−3.52	0.0034	15
Classical control	MobileNetV2	−5.75	−1.93	0.075	15
Classical control	ResNet-50	−7.05	−3.33	0.005	15
Deeper control	DenseNet-121	−6.02	−3.27	0.0056	15
Deeper control	EfficientNet-B0	−2.46	−1.57	0.14	15
Deeper control	MobileNetV2	−5.13	−1.72	0.11	15
Deeper control	ResNet-50	−5.22	−2.34	0.035	15
Fourier surrogate	DenseNet-121	−5.84	−4.56	0.00045	15
Fourier surrogate	EfficientNet-B0	−3.53	−1.94	0.072	15
Fourier surrogate	MobileNetV2	−5.71	−1.99	0.066	15
Fourier surrogate	ResNet-50	−6.02	−3.09	0.008	15
Bottleneck only	DenseNet-121	−8.38	−5.58	6.8e-05	15
Bottleneck only	EfficientNet-B0	−5.67	−4.06	0.0012	15
Bottleneck only	MobileNetV2	−7.86	−2.75	0.016	15
Bottleneck only	ResNet-50	−9.44	−4.43	0.00057	15
Linear probe	DenseNet-121	−8.58	−5.99	3.3e-05	15
Linear probe	EfficientNet-B0	−6.15	−4.58	0.00043	15
Linear probe	MobileNetV2	−8.56	−2.99	0.0098	15
Linear probe	ResNet-50	−10.00	−4.94	0.00022	15
MLP	DenseNet-121	−9.29	−7.47	3e-06	15
MLP	EfficientNet-B0	−5.96	−4.42	0.00059	15
MLP	MobileNetV2	−8.94	−3.01	0.0093	15
MLP	ResNet-50	−10.26	−5.47	8.3e-05	15

Nadeau–Bengio correction of the variance for overlapping training sets, factor $1/k + n\_test/n\_train$ with $n\_test/n\_train = 1/4$; two-sided p from the t distribution with $k - 1$ degrees of freedom.

Table S13. Shortcut and source controls on frozen features (Protocol 3), all heads and backbones.

Backbone	Head	Original images	Standardized images	Figshare tumors, unoccluded	Figshare tumors, occluded	Occluded tumors called no tumor	Single source, three tumor classes	Cross-source accuracy	Cross-source balanced accuracy
EfficientNet-B0	VQC head	83.5	83.3	83.8	81.1	1.0	82.4	34.3	41.7
EfficientNet-B0	Classical control	86.7	86.0	86.4	82.8	0.2	87.5	37.0	51.3
EfficientNet-B0	Deeper control	83.7	84.1	84.3	80.9	0.8	87.1	35.9	51.0
EfficientNet-B0	Fourier surrogate	85.4	85.0	85.3	81.9	0.4	85.3	38.3	51.2
EfficientNet-B0	Bottleneck only	87.3	86.3	87.3	83.9	0.4	88.5	37.4	50.8
EfficientNet-B0	Linear probe	88.0	87.7	87.9	84.8	0.2	88.2	35.6	51.3
EfficientNet-B0	MLP	88.1	87.4	88.0	84.4	0.0	88.6	36.6	52.6
DenseNet-121	VQC head	79.0	75.9	82.2	79.8	1.9	83.9	42.9	50.4
DenseNet-121	Classical control	86.6	87.1	85.9	83.2	0.6	87.5	36.4	48.8
DenseNet-121	Deeper control	85.3	84.8	84.9	81.7	1.0	87.8	40.5	52.3
DenseNet-121	Fourier surrogate	85.4	84.6	84.1	83.0	0.3	86.2	36.3	49.3
DenseNet-121	Bottleneck only	87.5	86.3	87.0	84.5	0.5	88.5	35.9	48.4
DenseNet-121	Linear probe	88.5	87.7	88.3	85.7	0.3	88.6	35.4	49.4
DenseNet-121	MLP	88.8	88.3	88.7	86.2	0.3	88.9	34.8	50.3
ResNet-50	VQC head	74.2	74.8	74.0	73.9	4.3	74.9	33.2	42.1
ResNet-50	Classical control	84.6	83.4	84.2	81.8	0.4	84.2	33.4	46.9
ResNet-50	Deeper control	82.4	81.1	81.6	79.2	1.0	83.4	32.8	46.0
ResNet-50	Fourier surrogate	82.2	81.7	81.7	79.1	0.8	81.5	39.5	50.3
ResNet-50	Bottleneck only	86.3	85.1	85.6	82.6	0.2	83.8	37.2	49.2
ResNet-50	Linear probe	86.2	85.1	85.5	82.6	0.4	85.8	35.7	50.0
ResNet-50	MLP	86.2	85.9	85.5	82.9	0.2	85.6	36.4	52.8
MobileNetV2	VQC head	81.2	80.5	82.6	80.7	1.4	81.8	44.1	51.1
MobileNetV2	Classical control	85.2	84.8	86.0	84.2	0.7	86.6	37.8	52.2
MobileNetV2	Deeper control	82.7	82.9	83.4	81.9	0.9	86.6	40.6	55.2
MobileNetV2	Fourier surrogate	84.6	82.6	85.7	83.5	0.5	84.9	41.2	52.8
MobileNetV2	Bottleneck only	86.8	86.2	87.4	85.2	0.4	87.9	39.5	54.7
MobileNetV2	Linear probe	87.4	87.1	87.4	85.6	0.4	87.6	41.4	56.2
MobileNetV2	MLP	87.6	87.7	88.1	85.8	0.3	88.3	42.4	58.1

Accuracy (%). Original and standardized images: four classes, seed 0, mean over five folds. Occlusion: heads trained on unoccluded images (seed 0), evaluated on the figshare-derived tumor images of their test folds with the tumor replaced by the median head intensity. Single source: three-class tumor task restricted to figshare-derived images, patient-grouped five-fold cross-validation, mean over seeds. Cross-source: trained on all figshare-derived tumor images, tested on the 382 SARTAJ tumor images not linked to figshare (chance, 33.3%).

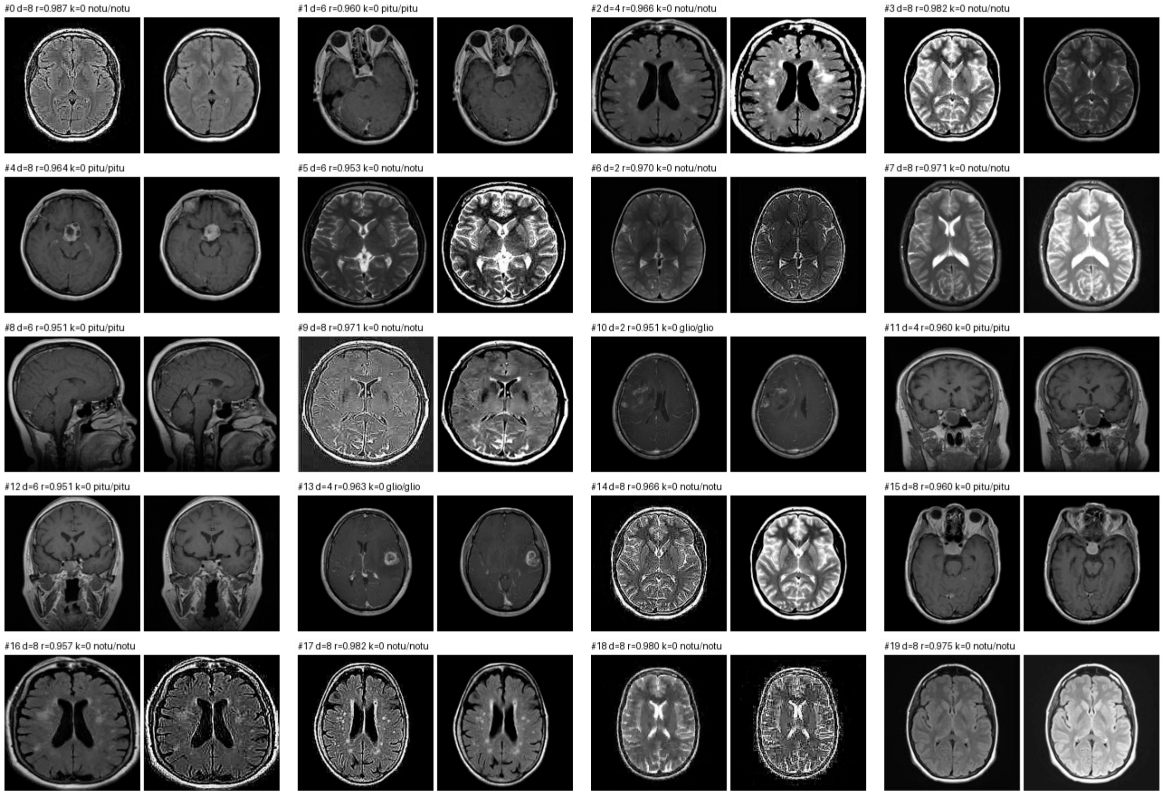
#0 d=8 r=0.987 k=0 notu/notu
#1 d=6 r=0.960 k=0 pitu/pitu
#2 d=4 r=0.966 k=0 notu/notu
#3 d=8 r=0.982 k=0 notu/notu
#4 d=8 r=0.964 k=0 pitu/pitu
#5 d=6 r=0.953 k=0 notu/notu
#6 d=2 r=0.970 k=0 notu/notu
#7 d=8 r=0.971 k=0 notu/notu
#8 d=6 r=0.951 k=0 pitu/pitu
#9 d=8 r=0.971 k=0 notu/notu
#10 d=2 r=0.951 k=0 glio/glio
#11 d=4 r=0.960 k=0 pitu/pitu
#12 d=6 r=0.951 k=0 pitu/pitu
#13 d=4 r=0.963 k=0 glio/glio
#14 d=8 r=0.966 k=0 notu/notu
#15 d=8 r=0.960 k=0 pitu/pitu
#16 d=8 r=0.957 k=0 notu/notu
#17 d=8 r=0.982 k=0 notu/notu
#18 d=8 r=0.980 k=0 notu/notu
#19 d=8 r=0.975 k=0 notu/notu

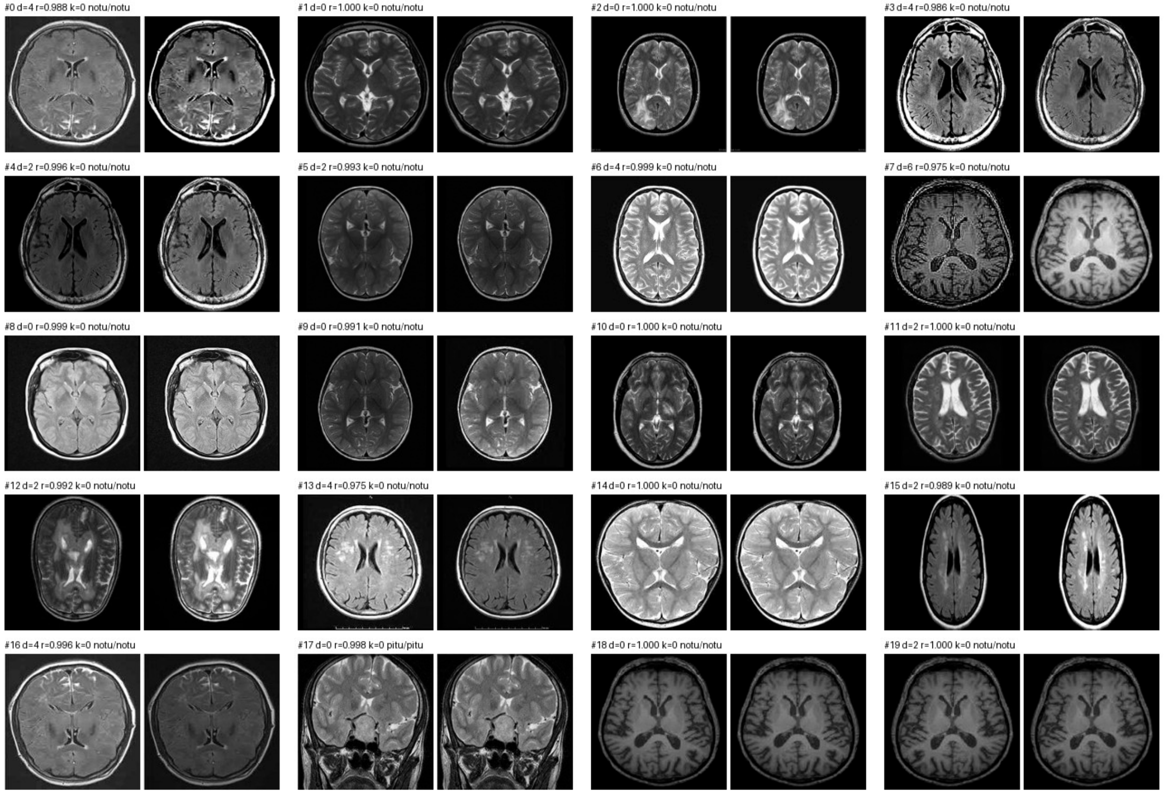
#0 d=4 r=0.988 k=0 notu/notu
#1 d=0 r=1.000 k=0 notu/notu
#2 d=0 r=1.000 k=0 notu/notu
#3 d=4 r=0.986 k=0 notu/notu
#4 d=2 r=0.996 k=0 notu/notu
#5 d=2 r=0.993 k=0 notu/notu
#6 d=4 r=0.999 k=0 notu/notu
#7 d=6 r=0.975 k=0 notu/notu
#8 d=0 r=0.999 k=0 notu/notu
#9 d=0 r=0.991 k=0 notu/notu
#10 d=0 r=1.000 k=0 notu/notu
#11 d=2 r=1.000 k=0 notu/notu
#12 d=2 r=0.992 k=0 notu/notu
#13 d=4 r=0.975 k=0 notu/notu
#14 d=0 r=1.000 k=0 notu/notu
#15 d=2 r=0.989 k=0 notu/notu
#16 d=4 r=0.996 k=0 notu/notu
#17 d=0 r=0.998 k=0 pitu/pitu
#18 d=0 r=1.000 k=0 notu/notu
#19 d=2 r=1.000 k=0 notu/notu

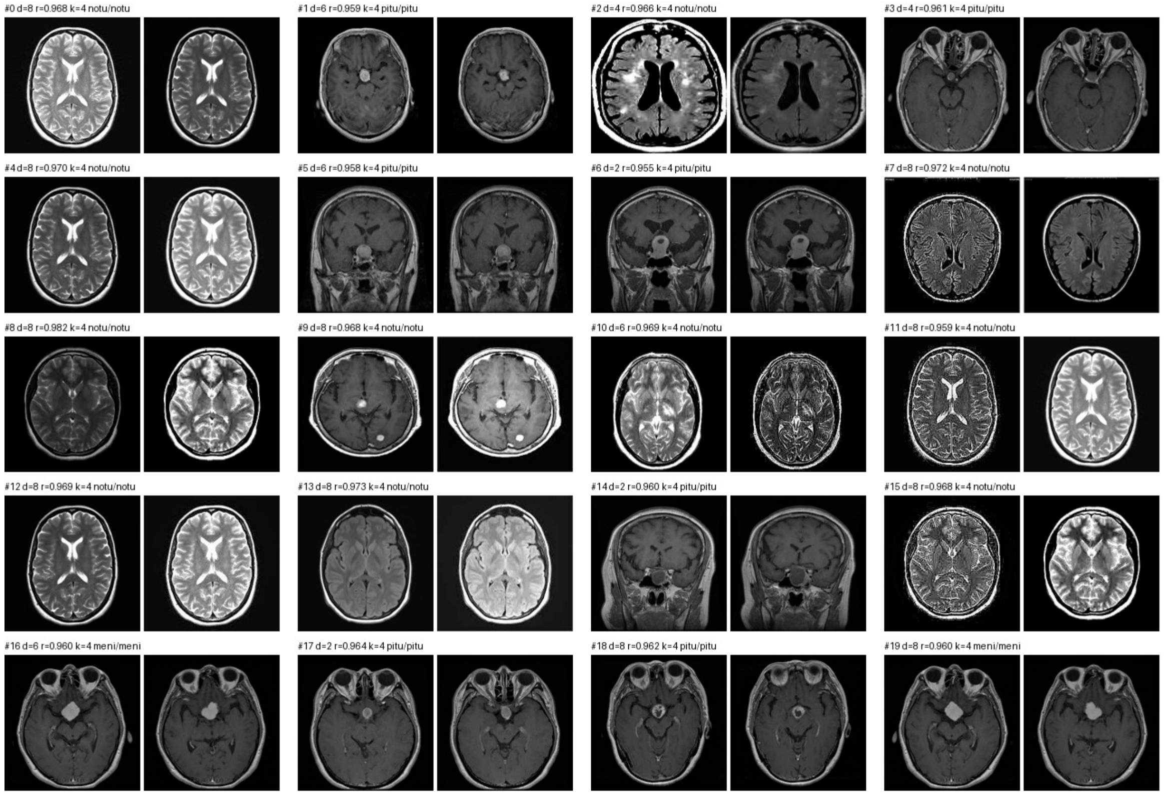
#0 d=8 r=0.968 k=4 notu/notu
#1 d=6 r=0.959 k=4 pitu/pitu
#2 d=4 r=0.966 k=4 notu/notu
#3 d=4 r=0.961 k=4 pitu/pitu
#4 d=8 r=0.970 k=4 notu/notu
#5 d=6 r=0.958 k=4 pitu/pitu
#6 d=2 r=0.955 k=4 pitu/pitu
#7 d=8 r=0.972 k=4 notu/notu
#8 d=8 r=0.982 k=4 notu/notu
#9 d=8 r=0.968 k=4 notu/notu
#10 d=6 r=0.969 k=4 notu/notu
#11 d=8 r=0.959 k=4 notu/notu
#12 d=8 r=0.969 k=4 notu/notu
#13 d=8 r=0.973 k=4 notu/notu
#14 d=2 r=0.960 k=4 pitu/pitu
#15 d=8 r=0.968 k=4 notu/notu
#16 d=6 r=0.960 k=4 meni/meni
#17 d=2 r=0.964 k=4 pitu/pitu
#18 d=8 r=0.962 k=4 pitu/pitu
#19 d=8 r=0.960 k=4 meni/meni

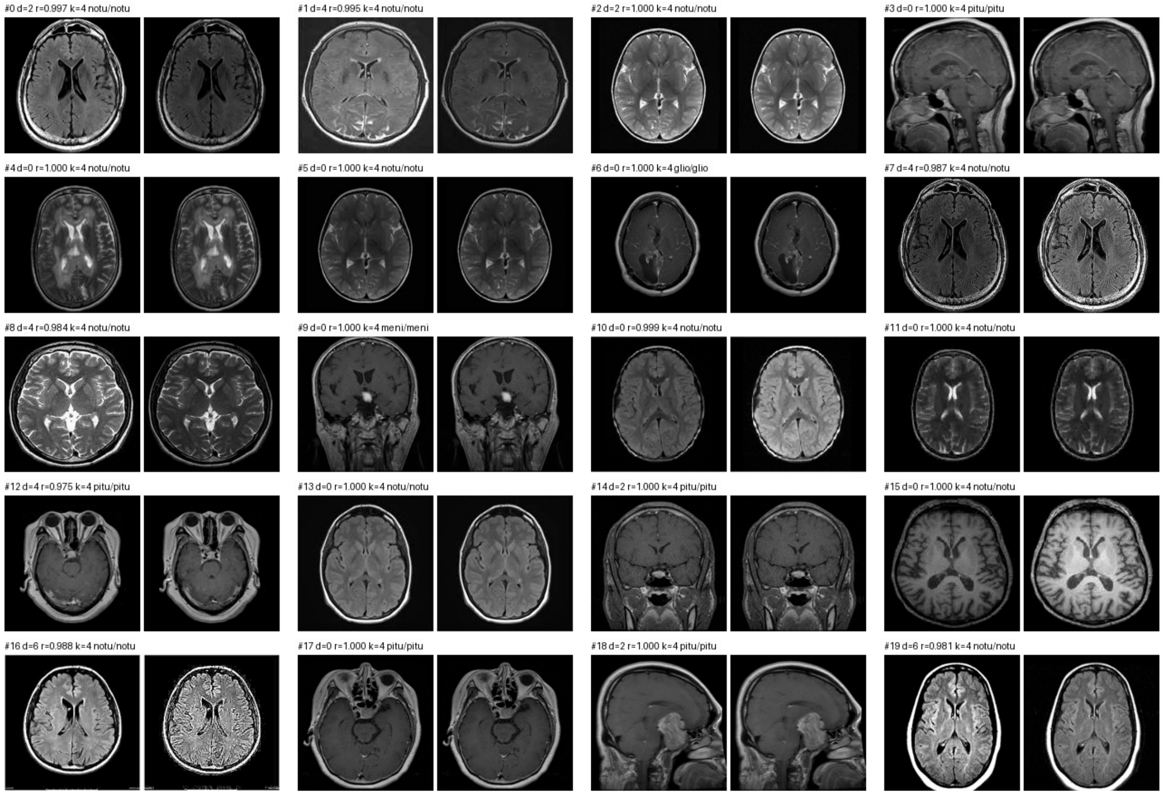

#0 d=2 r=0.997 k=4 notu/notu
#1 d=4 r=0.995 k=4 notu/notu
#2 d=2 r=1.000 k=4 notu/notu
#3 d=0 r=1.000 k=4 pitu/pitu
#4 d=0 r=1.000 k=4 notu/notu
#5 d=0 r=1.000 k=4 notu/notu
#6 d=0 r=1.000 k=4 glio/glio
#7 d=4 r=0.987 k=4 notu/notu
#8 d=4 r=0.984 k=4 notu/notu
#9 d=0 r=1.000 k=4 meni/meni
#10 d=0 r=0.999 k=4 notu/notu
#11 d=0 r=1.000 k=4 notu/notu
#12 d=4 r=0.975 k=4 pitu/pitu
#13 d=0 r=1.000 k=4 notu/notu
#14 d=2 r=1.000 k=4 pitu/pitu
#15 d=0 r=1.000 k=4 notu/notu
#16 d=6 r=0.988 k=4 notu/notu
#17 d=0 r=1.000 k=4 pitu/pitu
#18 d=2 r=1.000 k=4 pitu/pitu
#19 d=6 r=0.981 k=4 notu/notu

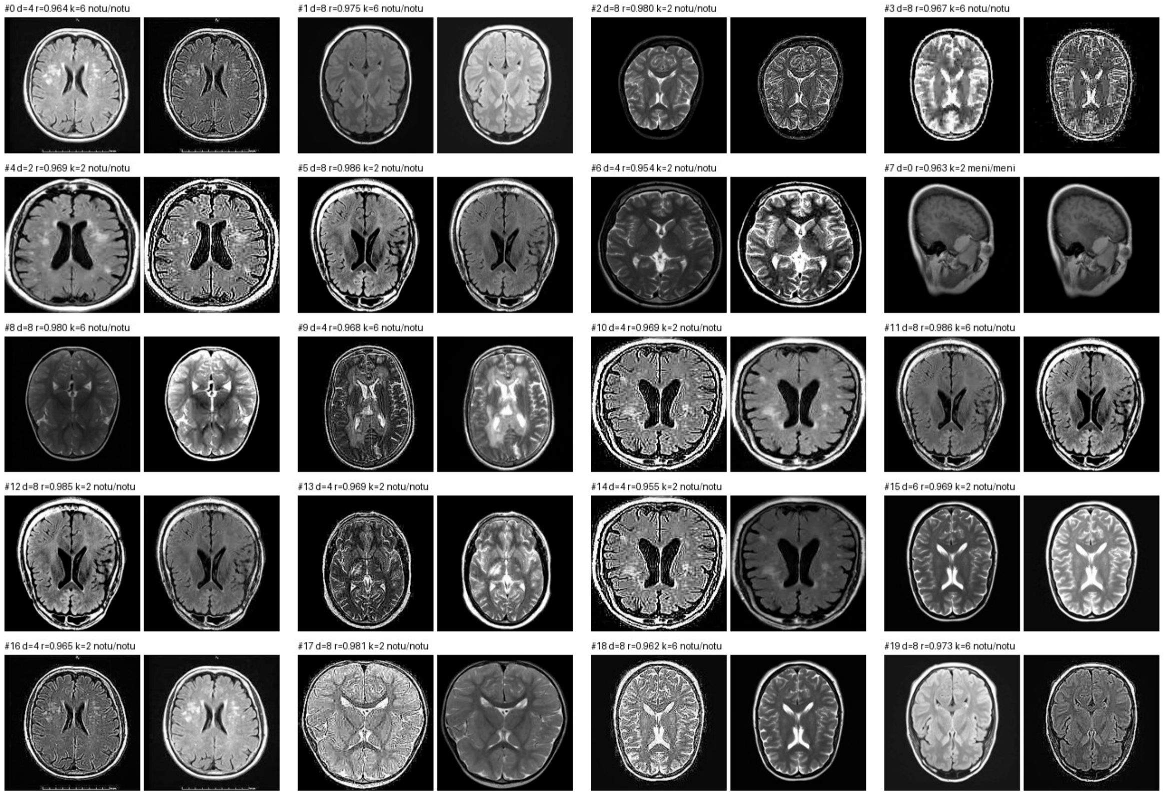
#0 d=4 r=0.964 k=6 notu/notu
#1 d=8 r=0.975 k=6 notu/notu
#2 d=8 r=0.980 k=2 notu/notu
#3 d=8 r=0.967 k=6 notu/notu
#4 d=2 r=0.969 k=2 notu/notu
#5 d=8 r=0.986 k=2 notu/notu
#6 d=4 r=0.954 k=2 notu/notu
#7 d=0 r=0.963 k=2 meni/meni
#8 d=8 r=0.980 k=6 notu/notu
#9 d=4 r=0.968 k=6 notu/notu
#10 d=4 r=0.969 k=2 notu/notu
#11 d=8 r=0.986 k=6 notu/notu
#12 d=8 r=0.985 k=2 notu/notu
#13 d=4 r=0.969 k=2 notu/notu
#14 d=4 r=0.955 k=2 notu/notu
#15 d=6 r=0.969 k=2 notu/notu
#16 d=4 r=0.965 k=2 notu/notu
#17 d=8 r=0.981 k=2 notu/notu
#18 d=8 r=0.962 k=6 notu/notu
#19 d=8 r=0.973 k=6 notu/notu

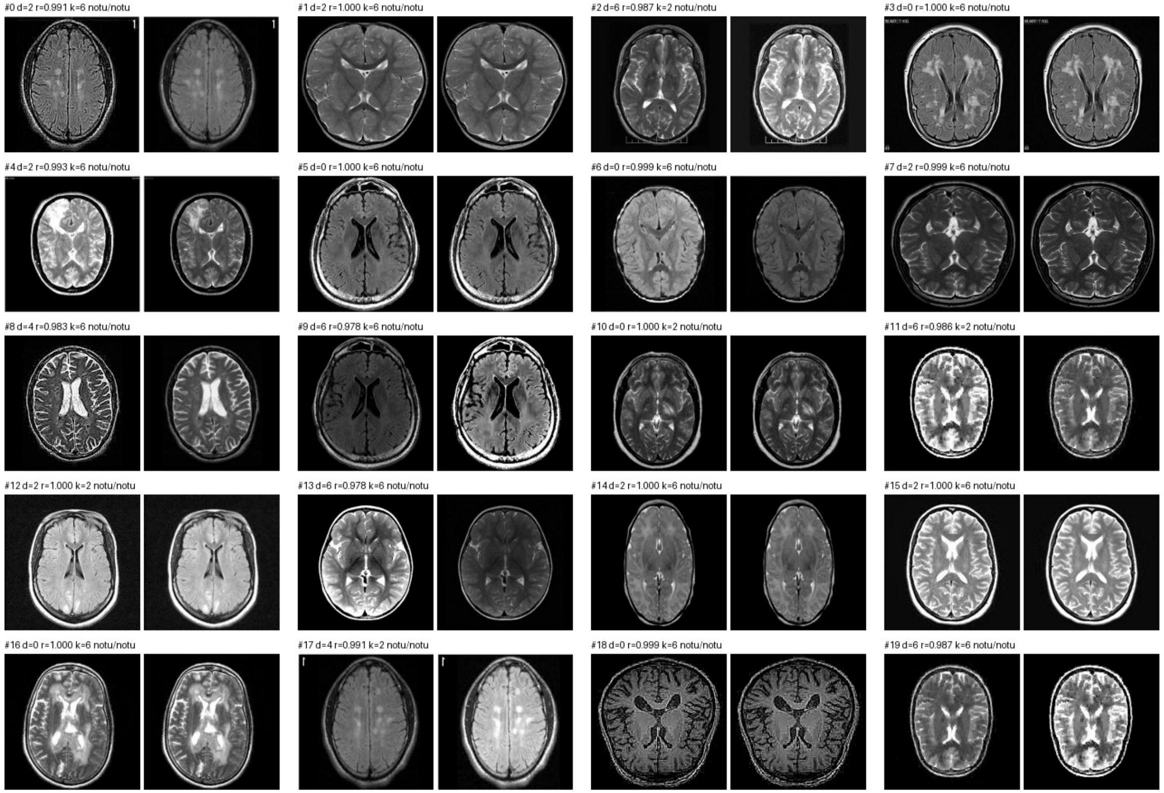
#0 d=2 r=0.991 k=6 notu/notu
#1 d=2 r=1.000 k=6 notu/notu
#2 d=6 r=0.987 k=2 notu/notu
#3 d=0 r=1.000 k=6 notu/notu
#4 d=2 r=0.993 k=6 notu/notu
#5 d=0 r=1.000 k=6 notu/notu
#6 d=0 r=0.999 k=6 notu/notu
#7 d=2 r=0.999 k=6 notu/notu
#8 d=4 r=0.983 k=6 notu/notu
#9 d=6 r=0.978 k=6 notu/notu
#10 d=0 r=1.000 k=2 notu/notu
#11 d=6 r=0.986 k=2 notu/notu
#12 d=2 r=1.000 k=2 notu/notu
#13 d=6 r=0.978 k=6 notu/notu
#14 d=2 r=1.000 k=6 notu/notu
#15 d=2 r=1.000 k=6 notu/notu
#16 d=0 r=1.000 k=6 notu/notu
#17 d=4 r=0.991 k=2 notu/notu
#18 d=0 r=0.999 k=6 notu/notu
#19 d=6 r=0.987 k=6 notu/notu

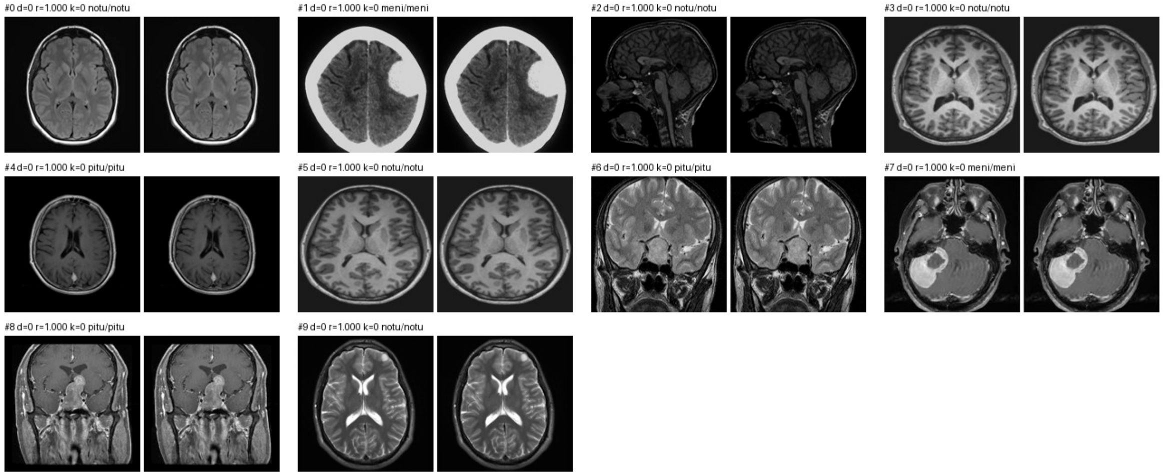
#0 d=0 r=1.000 k=0 notu/notu
#1 d=0 r=1.000 k=0 meni/meni
#2 d=0 r=1.000 k=0 notu/notu
#3 d=0 r=1.000 k=0 notu/notu
#4 d=0 r=1.000 k=0 pitu/pitu
#5 d=0 r=1.000 k=0 notu/notu
#6 d=0 r=1.000 k=0 pitu/pitu
#7 d=0 r=1.000 k=0 meni/meni
#8 d=0 r=1.000 k=0 pitu/pitu
#9 d=0 r=1.000 k=0 notu/notu

Figure S1. Randomly sampled duplicate pairs accepted by the revised detector (stratified by type: near, mirrored and rotated pairs, half of them close to a threshold, and exact pairs; 130 pairs on seven sheets). In each pair the second image is shown after the rotation or reflection that matched it; d, Hamming distance of the perceptual hashes; r, normalized cross-correlation of the thumbnails; k, index of the transformation.

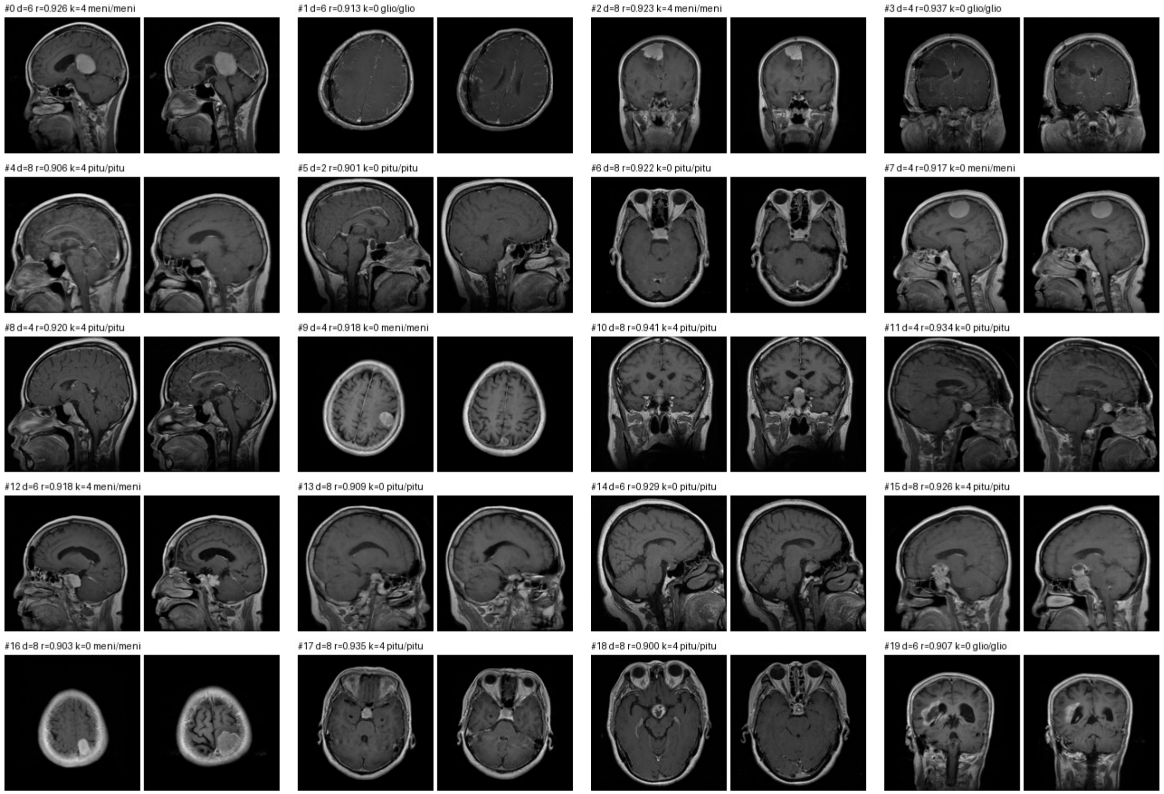
#0 d=6 r=0.926 k=4 meni/meni
#1 d=6 r=0.913 k=0 glio/glio
#2 d=8 r=0.923 k=4 meni/meni
#3 d=4 r=0.937 k=0 glio/glio
#4 d=8 r=0.906 k=4 pitu/pitu
#5 d=2 r=0.901 k=0 pitu/pitu
#6 d=8 r=0.922 k=0 pitu/pitu
#7 d=4 r=0.917 k=0 meni/meni
#8 d=4 r=0.920 k=4 pitu/pitu
#9 d=4 r=0.918 k=0 meni/meni
#10 d=8 r=0.941 k=4 pitu/pitu
#11 d=4 r=0.934 k=0 pitu/pitu
#12 d=6 r=0.918 k=4 meni/meni
#13 d=8 r=0.909 k=0 pitu/pitu
#14 d=6 r=0.929 k=0 pitu/pitu
#15 d=8 r=0.926 k=4 pitu/pitu
#16 d=8 r=0.903 k=0 meni/meni
#17 d=8 r=0.935 k=4 pitu/pitu
#18 d=8 r=0.900 k=4 pitu/pitu
#19 d=6 r=0.907 k=0 glio/glio

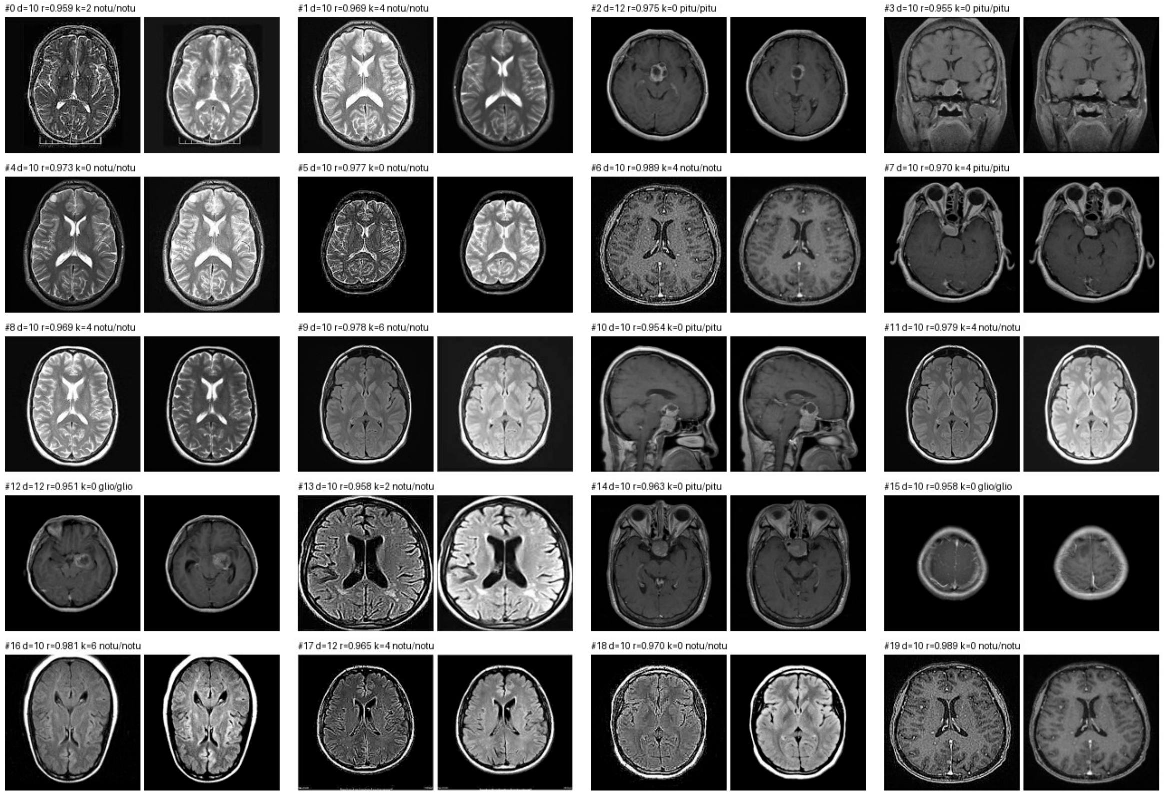

Figure S1 (continued). Randomly sampled rejected candidates (NCC 0.90–0.95, or Hamming distance 9–12 bits with NCC ≥ 0.95; 40 pairs on two sheets).

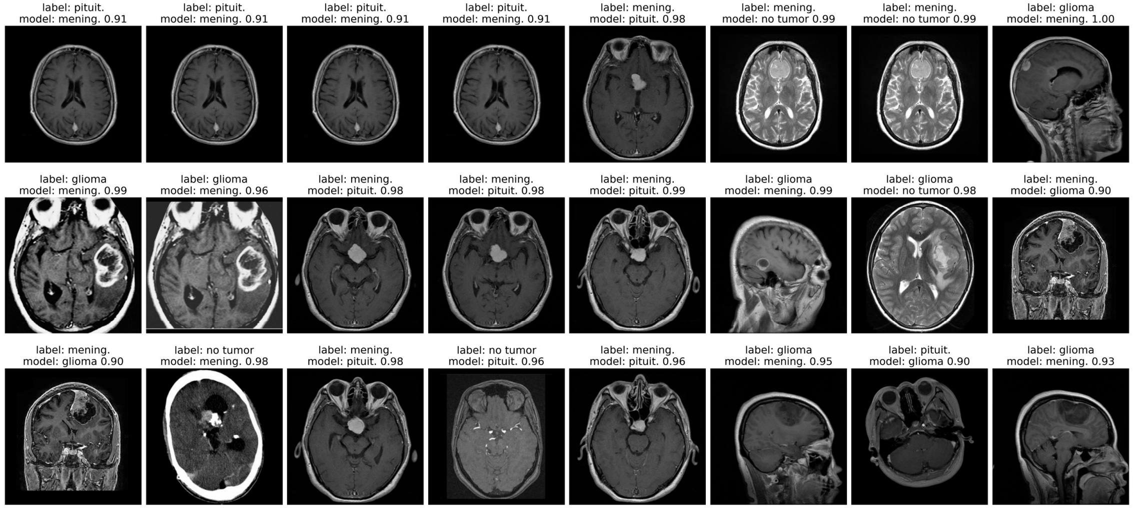

Figure S2. Candidate label errors flagged by confident learning (out-of-fold probabilities of the MLP heads, ensemble over four frozen backbones): given label, suggested label and its probability. These are candidates for expert review, not confirmed errors.

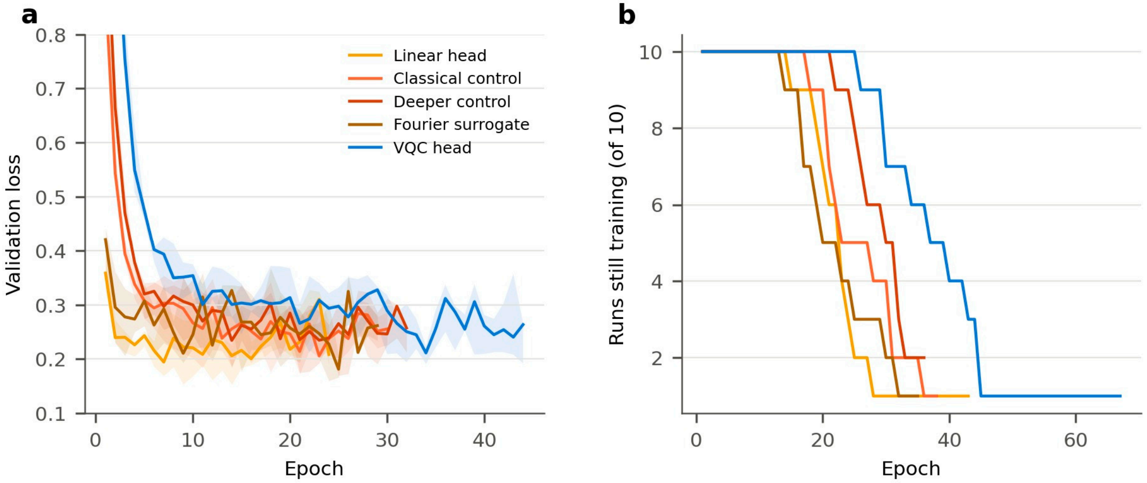

Figure S3. End-to-end EfficientNet-B0 heads trained with the convergence-based recipe (block B, Protocol 3; ten runs per head). **a** Validation loss per epoch (median; shaded, interquartile range; shown while at least three runs were training). **b** Number of runs still training.

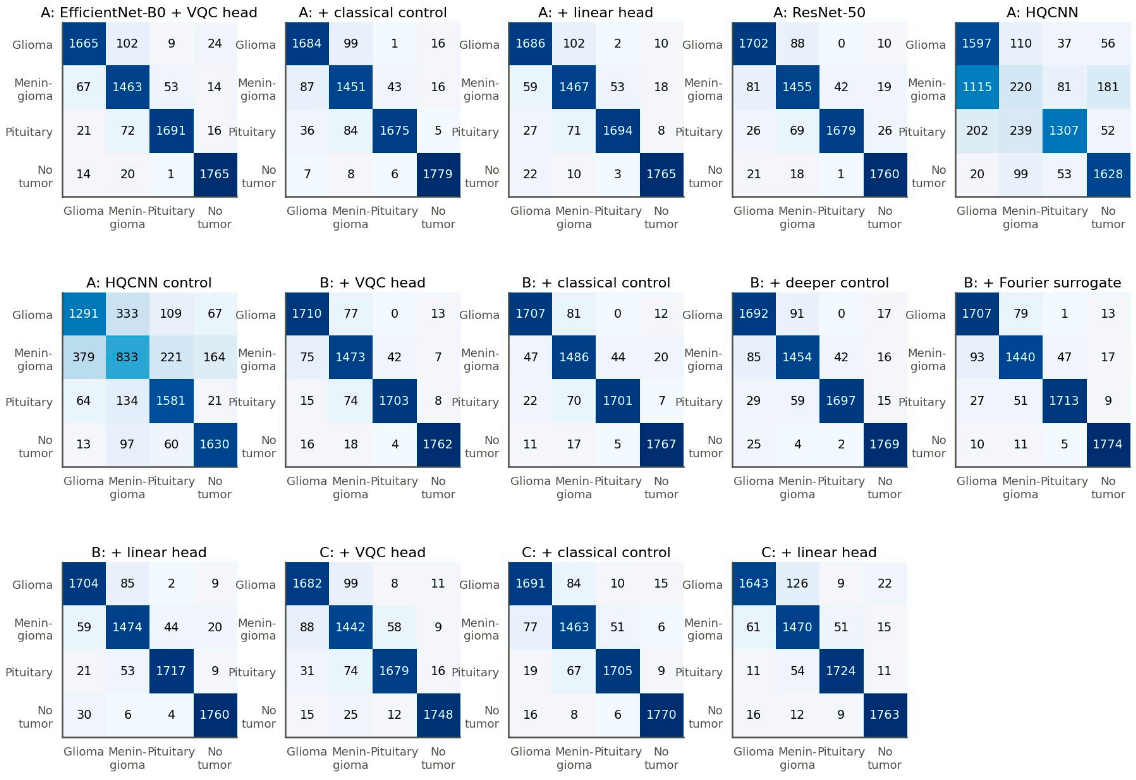

Figure S4. Confusion matrices of the end-to-end models under Protocol 3 (out-of-fold predictions for the 6997 non-augmented images; block B: ensemble of two seeds); cell shading, row-normalized frequency.

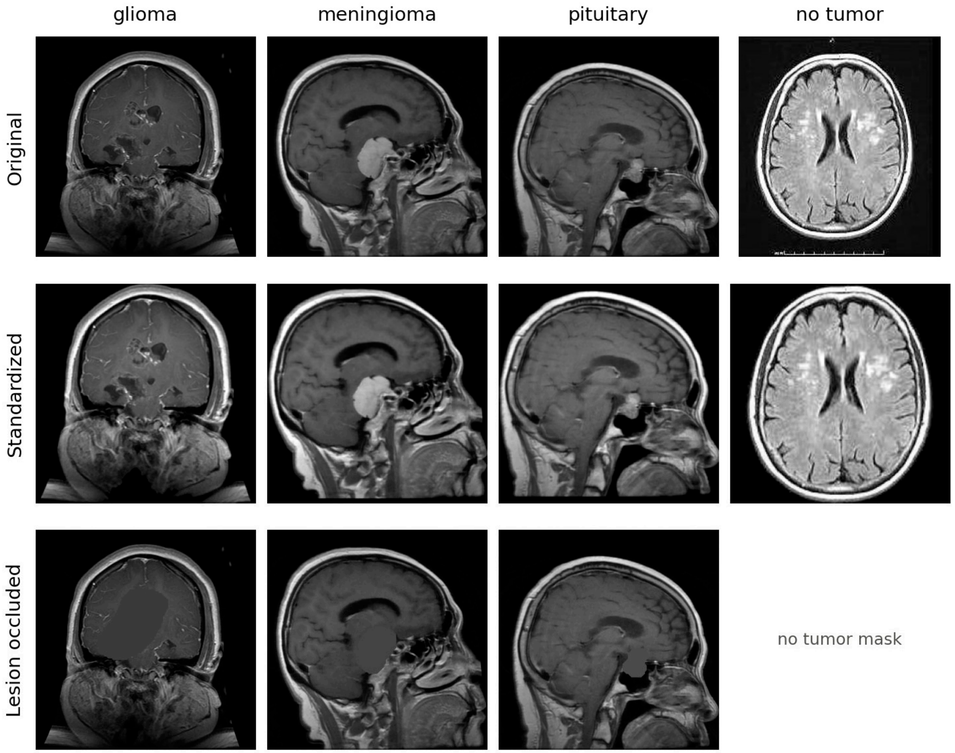

Figure S5. Image variants used in the shortcut and source controls (Section 2.9): original image, standardized image (head mask, crop, intensity rescaling to the 1st–99th percentile inside the mask, 256 × 256 pixels) and, for figshare-derived tumor images, the lesion-occluded image (manual tumor mask dilated by 8 pixels and filled with the median head intensity).
